# Dicer dependent tRNA derived small RNAs promote nascent RNA silencing

**DOI:** 10.1093/nar/gkac022

**Published:** 2022-01-20

**Authors:** Arianna Di Fazio, Margarita Schlackow, Sheng Kai Pong, Adele Alagia, Monika Gullerova

**Affiliations:** Sir William Dunn School of Pathology, University of Oxford, South Parks Road, Oxford OX1 3RE, UK; Sir William Dunn School of Pathology, University of Oxford, South Parks Road, Oxford OX1 3RE, UK; Sir William Dunn School of Pathology, University of Oxford, South Parks Road, Oxford OX1 3RE, UK; Sir William Dunn School of Pathology, University of Oxford, South Parks Road, Oxford OX1 3RE, UK; Sir William Dunn School of Pathology, University of Oxford, South Parks Road, Oxford OX1 3RE, UK

## Abstract

In mammalian cells, small non-coding RNAs (sncRNAs) negatively regulate gene expression in a pathway known as RNA interference (RNAi). RNAi can be categorized into post-transcriptional gene silencing (PTGS), which involves the cleavage of target messenger RNA (mRNA) or inhibition of translation in the cytoplasm, and transcriptional gene silencing (TGS), which is mediated by the establishment of repressive epigenetic marks at target loci. Transfer RNAs (tRNAs), which are essential for translation, can be processed into small ncRNAs, termed tRNA-derived small RNAs (tsRNAs). The biogenesis of tsRNAs and their role in gene expression regulation has not yet been fully understood. Here, we show that Dicer dependent tsRNAs promote gene silencing through a mechanism distinct from PTGS and TGS. tsRNAs can lead to downregulation of target genes by targeting introns via nascent RNA silencing (NRS) in nuclei. Furthermore, we show that Ago2 slicer activity is required for this mechanism. Synthetic tsRNAs can significantly reduce expression of a target gene at both RNA and protein levels. Target genes regulated by NRS are associated with various diseases, which further underpins its biological significance. Finally, we show that NRS is evolutionarily conserved and has the potential to be explored as a novel synthetic sRNA based therapeutic.

## INTRODUCTION

In the RNA interference (RNAi) pathway, regulatory small non-coding RNAs (sncRNAs) guide RNA-induced silencing complex (RISC) to silence gene expression by direct cleavage of target messenger RNAs (mRNAs) (1) or translational repression in the cytoplasm (2). This mechanism is known as posttranscriptional gene silencing (PTGS). In transcriptional gene silencing (TGS), another mode of RNAi, gene expression is suppressed at the transcriptional level via chromatin modifications. The RNA-induced transcriptional silencing complex (RITSC) recruits chromatin-modifying proteins to form heterochromatin locally (3,4).

Increasing depth of RNA sequencing has enabled the discovery of a highly evolutionarily conserved group of sncRNAs, tRNA-derived small RNAs (tsRNAs) or tRNA-derived fragments (tRFs), in various species including mouse and humans (5–7). Depending on their precursors and original positions within the tRNA, tRFs can be subdivided into four categories: tRF-5, tRF-3, tRF-1 and internal tRF (itRF) (8). tRF-5s are derived from the 5′ end of tRNA, starting from the anticodon loop or the D loop till the 5′ terminal. tRF-3s originate from the 3′ end of its mature precursor while tRF-1 sequences correspond to the tRNA 3′ trailer sequence removed by RNase Z, its 3′ end matches the termination signal for RNA pol III (9,10). itRFs represent a group of tRFs corresponding to the region around the anticodon loop of mature tRNAs.

The detail of tRF biogenesis is unclear. Cole *et al.* showed that a cytoplasmic tRF-5 derived from tRNA^Gln^ is Dicer-dependent based on the evidence that the tRF is destabilized upon Dicer depletion in HeLa and HEK293 cells and Dicer alone is sufficient to generate the tRF from tRNA^Gln^ precursor (11). Haussecker *et al.* described a group of Drosha-independent, Dicer-dependent tRF-3s (Type I) which associate with Ago proteins (12). Evidence for Dicer-dependent tRFs are also found in other organisms. In zebrafish, tRF-5s derived from tRNA^Glu-CTC^ and tRNA^Pro-CGG^ are shown to be cleaved specifically by Dicer *in vitro*. Using an RNA secondary prediction software RNAShapes, Soares *et al.* predicted that 5% of tRNA^Glu^ and 50% tRNA^Pro^ are prone to form long hairpin structures, which can be recognized by Dicer (13). In *Arabidopsis thaliana*, tRF-5 accumulation can be induced by the knockout of the chromatin remodelling protein Decrease in DNA Methylation 1 (DDM1); in *dcr1*/*ddm1* double mutants, the accumulation is reduced, implying that Dicer-like1 (DCL1) has a role in the biogenesis of tRF-5s (14). On the other hand, several studies have claimed that Dicer is not necessary for the generation of most tRFs (15–17). Meta-analysis of small RNA sequencing datasets from mouse, *S. pombe* and *Drosophila* showed that mutation of Dicer does not influence the levels of tRFs in these organisms (15). A subsequent report by the same group claimed that tRF-3 generation is intact in Dicer knockout HEK293T cells (18).

Previously considered to be random degradation products, tsRNAs have gradually been recognized as *bona fide* regulatory sncRNAs. A tRF-5 from tRNA^Gln^ was shown to inhibit protein translation without affecting mRNA abundance or polyadenylation in a sequence-independent manner. The exact mechanism, however, is unclear (19). Furthermore, tRFs play a role in regulating ribosome biogenesis. tRF-3 from tRNA^Leu-CAG^ binds to the mRNAs of two ribosomal proteins, RPS28 and RPS15, promoting their translation. This in turn enhances processing of 18S ribosome and increases production of 40S ribosomal subunits (20). The association of tRFs with Ago proteins implies that they may be involved in RNAi. Dicer-dependent (or Type I) tRFs co-immunoprecipitate with all four FLAG-tagged Ago proteins while RNaseZ-dependent, Dicer-independent tRFs show a preference for FLAG-tagged Ago3 and 4 (12). Meta-analysis of Ago PAR-CLIP data by Kumar *et al.* revealed that tRF-3s and tRF5-s strongly associate with Ago1, 3 and 4 but not Ago2^15^. In B cells, Dicer-dependent CU1276, a tRF-3, can downregulate endogenous RPA1 by interacting with its 3′ UTR in a miRNA-like manner (21). Additionally, a recent study by Kuscu *et al.* has shown that overexpression of tRNA can lead to increased levels of tRF-3s resulting in global silencing of mRNA targets whose 3′ UTR sequences are complementary to at least the 6-mer sequence of the corresponding tRF-3. They also showed that tRF-3 associates with Trinucleotide repeat-containing gene 6 (TNRC6; also known as GW182) proteins, indicating the gene silencing is achieved by mRNA destabilization (18).

Production of functional, mature mRNA is regulated at many levels. Splicing occurs co-transcriptionally, as soon as the first splice site appears on nascent RNA. Splicing efficiency then feeds back to the progression of transcription elongation and correct termination. However, the kinetic coupling between splicing and transcription is not well understood. Only recently, direct nascent RNA sequencing had shown that there is a delay between transcription and splicing in human cells (22). Furthermore, large number of mRNAs contain introns that were not spliced out (23). Therefore, introns have been implied as a platform for gene expression regulation.

Here, we provide evidence for a novel gene silencing pathway mediated by Dicer dependent tsRNAs which is distinct from PTGS or TGS. Notably, tsRNAs guide Ago2 proteins to target protein coding transcripts and long non-coding RNA in the nucleus leading to nascent RNA silencing (NRS). Elevated expression of target genes regulated by this pathway is associated with various diseases, further substantiating the biological significance of NRS.

## MATERIALS AND METHODS

### Cell lines and tissue culture

The cell lines used in this study were wild type human embryonic kidney 293T (HEK293T cells), recombinant HEK293T-REx clone 1.3 cells with integrated doxycycline-inducible expression cassettes containing TAP-tagged Dicer alongside short hairpin RNA (shRNA) against *DICER* mRNA (shDicer), recombinant HEK293T-REx cell lines with integrated doxycycline-inducible expression cassettes containing shDicer and shRNA against *AGO2* mRNA (shAgo2) respectively and recombinant HEK293T-REx cell lines with integrated expression cassettes for FLAG-Ago2 wt and FLAG-Ago2^D669A^ mutant, cancer cell lines BT-549, MCF7 andA549, and Ago2^–/–^ mouse embryonic fibroblasts. All cells were cultured in Dulbecco's modified Eagle's medium (DMEM) (Thermo Fisher Scientific) with 10% foetal bovine serum (FBS), 1% l-glutamine (Gibco) and 1% penicillin–streptomycin (Gibco) at 5% CO_2_ and 37°C. Dicer and Ago2 knockdown were achieved by incubating the inducible cell lines with doxycycline (3 μg/ml) in DMEM for 72 h (replaced with fresh media with doxycycline every 24 h) at 37°C.

### Transfection of plasmids and sRNA

Transfection of shRNA against *DROSHA* mRNA (shDrosha)-containing plasmids (10 μg for 6 h twice) and synthetic tsRNA (50 μM for 48 h) in wild type HEK293T-and recombinant HEK293T-Rex cell lines was performed using Lipofectamine 2000 reagent (Invitrogen). Transfection of synthetic tsRNA (100 pmol for 24 h) in BT549, A549 and MCF7 cells and HEK293T (500 nM for 48 h) was performed using Lipofectamine RNAiMAX (Invitrogen). Wild type HEK293T cells were incubated with α-Amanitin (2 μg/ml, Sigma) for 24 h to inhibit transcription. Transfection of plasmids (1 μg per well in 6-well plates) expressing wild type and mutant human Ago2 into HEK293 cells was performed using Lipofectamine LTX (9 μl/well in six-well plates) with PLUS reagent (1 μl/μg of plasmid). Transfection of plasmid expressing FLAG-Dicer (10 μg per 10 cm dish) was performed using Lipofectamine 2000 and collected 48 h later.

### sRNA-seq and mRNA-seq sample preparation

For sRNA-seq, total RNA was isolated from cells treated with scrambled shRNA (as control) and shDicer cells for 7 days using the miRVana miRNA Isolation Kit (Thermo Fisher Scientific). Quality of purified RNA was confirmed with RNA 6000 Pico Kit (Agilent) on the Agilent 2100 Bioanalyzer. Sequencing libraries were prepared using the NEBNext® Multiplex Small RNA Library Prep Set (New England BioLabs) and sequenced on a HiSeq2000 (Illumina).

For mRNA-seq, RNA was purified using the miRNEasy Kit (Qiagen) and treated with DNase (Thermo Fisher Scientific) at 37°C for 30 min followed by acidic phenol-chloroform extraction. Sample integrity was verified with a 1.25% formaldehyde gel. RNA samples were ribo-depleted and sequencing libraries preparation was performed with the TruSeq Stranded Total RNA Sample Preparation Kit (Illumina) followed by paired-end sequencing on HiSeq2000 (Illumina).

### Northern blot

For detection of tsRNAs, 30 μg of total RNA in 2× Novex TBE–urea sample buffer (2×) (Thermo Fisher Scientific) and separated on 12% denaturing (50% g/v urea) polyacrylamide gel in 1× TBE. RNA was transferred onto hybond-N + nylon membrane at 5 V for 1 h followed by UV-crosslinking and pre-hybridization in ULTRAhyb-Oligo buffer (Thermo Fisher Scientific) at 42°C for at least 30 min. tRNA/tsRNA-specific oligonucleotide probes were radiolabelled with ^32^P-ATP by poly nucleotide kinase (PNK) (PerkinElmer) at 37°C for 1 h. Radio-labelled probes were purified in G-25 Sephadex columns (GE Healthcare) and hybridized onto the membrane at 42°C followed by washes with 0.1× SCC washing buffer (0.05% SDS) and subjected to autoradiography.

### T7 transcription

DNA template for *in vitro* transcription were purchased as ssDNA oligonucleotides from IDT. Equimolar amounts of antisense and sense DNA strands were annealed using annealing buffer (10 mM Tris–HCl pH 7.6, 50 mM NaCl and 1 mM EDTA pH 8). After incubation at 95°C for 3 min, dsDNA template was allowed to slowly cool down to room temperature.

Following *in vitro* transcription with HiScribe T7 High Yield RNA Synthesis Kit (New England Biolabs) at 37°C for 16hr, RNA was treated with DNAse I RNAse free (1 U, Roche) for 1 h at 37°C and then extracted with TRIzol LS reagent (Invitrogen).

Isolated RNA was resuspended in Novex TBE-Urea loading Buffer (Invitrogen) and separated on a denaturing gel (10% 19:1 polyacrylamide/bisacrylamide, 1x TBE, 8 M urea). RNA was visualized under UV light using a transilluminator (UVP) and bands corresponding to full length RNA were excised. Gel slices were crushed and RNA was extracted in crush soak Buffer (500 mM NH_4_CH_3_CO_2_, 100 mM EDTA pH 8, 0.1% SDS) for 18 h followed by ethanol-precipitation.

### *In vitro* Dicer cleavage assays

TAP-tagged Dicer protein expression was induced with 3 μg/mL doxycycline for 1 week in HEK 293T cells.

1 × 10^8^ cells were harvested, washed twice in PBS and lysed for 30 min at 4°C in lysis buffer (20mM Tris–HCl pH 7.6, 137 mM NaCl, 3 mM MgCl_2_, 0.5% NP-40, 5% glycerol and proteases inhibitor). Insoluble cellular debris were pelleted by centrifugation and the clarified lysate was incubated with 50 μl IgG Sepharose 6 Fast Flow affinity resin (GE Healthcare) for 1.5 h at 4°C. After binding reaction, Sepharose beads were extensively washed before proceeding with the elution.

TAP-tagged Dicer was eluted by using AcTEV protease (Invitrogen) following manufacturer's instructions, then purified Dicer protein was resuspended in Dicer reaction buffer (50 mM Tris–HCl pH 8, 20 mM HEPES, 5 mM MgCl_2_, 5% glycerol, proteases inhibitor). 500 ng of purified RNA was incubated with Dicer at 37°C.

At appropriated times, aliquots of the reaction mixtures were separated, added to a Novex TBE-Urea loading Buffer and immediately frozen at −80°C. Then, all samples were heated for 3 min at 95°C immediately before gel electrophoresis. Cleavage products were separated on a 10% denaturing polyacrylamide gel and visualized by SYBR Gold (Invitrogen) staining or blotted onto hybond-N + nylon membrane and detected with specific probes visualized with autoradiography.

14 × 10^6^ HEK293T cells were transfected with 20 μg FLAG-Dicer plasmid and collected 48 hours later. Cells were lysed with lysis buffer (50 mM Tris–HCl pH 7.6, 150 mM NaCl, 3 mM MgCl_2_, 0.5% Triton X-100, 5% glycerol and 1× protease inhibitor) for 45 min at 4°C and sonicated for 10 s at 10 μm. FLAG-Dicer was pulled down using 100 μl of pre-equilibrated Anti-FLAG M2 magnetic beds (Sigma-Aldrich) for 1.5 h at 4°C. The beads were washed twice in TBS and FLAG-Dicer was eluted for 1 h at room temperature in Dicer reaction buffer (50 mM Tris–HCl pH 8, 20 mM HEPES, 5 mM MgCl_2_, 5% glycerol, proteases inhibitor) with 125 ng/μl of 3xFLAG peptide (Sigma-Aldrich). The eluate was used directly for Dicer cleavage *in vitro* reaction by adding 2 μl of RNase inhibitor (Ribolock) and 1 μg of tRNA and incubated at 37°C for up to 4 h. Aliquots of the reaction were collected and quenched in 2× TBE–urea and instantaneously frozen at −80°C. Cleavages products were separated on a 10% denaturing urea (50% w/v) polyacrylamide gel, the RNA was transferred onto hybond-N+ nylon membrane and detected with specific probes followed by autoradiography.

### qRT-PCR and stem loop qRT-PCR

RNA was isolated from whole-cell, cytoplasmic, nuclear or chromatin extracts with TRIzol LS Reagent (Invitrogen) as per manufacturer's instructions and treated with DNase I (1 U, Roche) for 30 min at 37 ºC. 250–500 ng (for steady-state mRNA detection), 5 μg of RNA (for nascent RNA detection) and 2 μg (for sRNA detection) were used for preparing cDNA template using SuperScript™ Reverse Transcriptase (Thermo Fisher Scientific) with specific reverse or stem loop primers. Real-time PCR was performed on Rotor-Gene RG3000 machine (Corbett Research) with SensiMix™ SYBR No-Rox Mastermix (Bioline Reagents) with specific primer pairs. Relative fold change was computed using the comparative Ct method.

### Subcellular fractionation

3 × 10^6^ cells were trypsinized, washed twice in ice-cold PBS and lysed in five volumes of hypotonic lysis buffer (10 mM HEPES pH 7.9, 60 mM KCl, 1.5 mM MgCl_2_, 1 mM EDTA, 1 mM DTT, 0.075% NP-40, 1× protease inhibitor cocktail (Roche)) for 10 min. Lysates were centrifuged at 500 × g for 5 min and resulting supernatant was collected as the cytoplasmic fraction. The nuclear pellet was washed gently for 3 times in 800 μl hypotonic lysis buffer without NP-40. Nuclei were lysed in one volume of nuclear lysis buffer (20 mM HEPES pH 7.9, 400 mM NaCl, 1.5 mM MgCl_2_, 0.2 mM EDTA, 1 mM DTT, 5% glycerol, 1× protease inhibitor cocktail (Roche)) and dilute with two volumes of dilution buffer (20 mM HEPES pH 7.9, 1.6% Triton-X-100, 0.2% sodium deoxycholate, 1× protease inhibitor cocktail (Roche)).

### Chromatin immunoprecipitation

Approximately 4 × 10^6^ cells were incubated with 1% formaldehyde in DMEM for 10 min at 37 ºC followed by quenching with 0.125 M glycine in DMEM for 10 min at 37 ºC. Cells were washed with ice-cold PBS and lysed in 800 μl cell lysis buffer (0.5% NP-40, 85 mM KCl, 5 mM PIPES, 1× protease inhibitor cocktail (Roche)). Nuclei were pelleted at 500 g for 5 min and lysed in 400 μl nuclear lysis buffer (50 mM Tris–HCl, 1% SDS, 10 mM EDTA, 1× protease inhibitor cocktail (Roche)) and sonicated at high power settings (30 s on/30 s off) for 15 min at 4ºC using Bioruptor sonicator (Diagenode). The cell debris were pelleted at 13 000 g for 10 min and the fragmented chromatin lysate was pre-cleared with protein A and G agarose beads (1:1 ratio) (50 μl per sample, Millipore) for 1 h, divided equally into input, IP and beads only samples and diluted in dilution buffer (16.7 mM Tris–HCl, 0.01% SDS, 1.1% Triton X-100, 500 mM EDTA, 167 mM NaCl, 1× protease inhibitor cocktail (Roche)). Immunoprecipitation with antibodies was performed overnight and samples were incubated with protein A/G agarose beads (60 μl) for 1h. Beads were washed with washing buffers A (20 mM Tris–HCl, 2 mM EDTA, 0.1% SDS, 1% Triton X-100, 150 mM NaCl), B (20 mM Tris–HCl, 2 mM EDTA, 0.1% SDS, 1% Triton X-100, 500 mM NaCl), C (10 mM Tris–HCl, 1 mM EDTA, 1% NP-40, 1% sodium deoxycholate, 0.25 M LiCl) and D (10 mM Tris–HCl, 1 mM EDTA). Protein–DNA complexes were eluted with elution buffer (1% SDS, 0.1 M NaHCO3) for 15 min twice at room temperature and treated with RNase A (10 μg) and proteinase K (20 μg) at 65ºC overnight. DNA was extracted with phenol-chloroform-isoamyl alcohol (25:24:1) mix and used followed by qPCR. For Ago2 immunoprecipitation, protein–RNA complexes were directly subjected to TRIzol treatment followed by northern blotting.

### Western blot

Whole cell, cytoplasmic, nuclear or chromatin extracts were treated directly with 4x Laemmli buffer (0.2 M Tris–HCl, 8% (w/v) SDS, 40% glycerol, 20% (v/v) β-mercaptoethanol, 0.005% bromophenol blue), incubated at 95ºC for 5 min and sonicated. Samples were separated on mini-PROTEAN^®^ TGX™ gels (Bio-Rad Laboratories) followed by transfer onto nitrocellulose membrane (Protran, GE Healthcare) and probed with antibodies.

### ChrRNA-seq sample preparation

Chromatin fraction was extracted using approximately 6.72 × 10^6^ cells according to a published protocol (21) and treated with 40 μg of proteinase K in 1% SDS and 1 μl of Turbo DNase (2 U/μl) (Thermo Fisher Scientific), which was followed by TRIzol (Invitrogen) extraction. Incompletely dissolved chromatin pellet was dissolved by heating the samples at 55ºC for 10 min on a heat block in safe lock tubes (Eppendorf).

### Labelling of sRNA with fluorescent dye

tsRNA and siRNA against *EGFR* were labelled with fluorescent dye by FISH Tag RNA Multicolor Kit (Invitrogen) according to manufacturer's instructions. Cells were fixed by paraformaldehyde (3%) 24 h post-transfection followed by confocal imaging. All images were captured under the same settings.

### CRISPR-Cas9 knockout of SPINT1 predicted target site

#### RNP formation

For RNP formation, 1 μg of each IVT sgRNA was added to 5 ug Cas9 HiFi protein (IDT) in a total volume of 2 μl IDT Duplex Buffer (IDT, cat. #11-05-01-12), resuspended 5 times and incubated at 37ºC for 5 min. Resulting RNP mixes were added directly to cells prior to nucleofection.

#### RNP nucleofection

Cells (2 × 10^5^) were washed twice with PBS and nucleofected with 5 μg Cas9 protein (IDT) and 1 μg sgRNA in a total volume of 10 μl ‘buffer R’ using the Neon Transfection System (Invitrogen, cat. #MPK5000).

#### Materials

Neon Transfection System (Invitrogen, cat. #MPK5000) and consumables Neon Transfection System 10 μl Kit (Invitrogen, cat. #MPK1096).

Buffers etc for nucleofection buffer R from Neon Transfection System 10 μl Kit (Invitrogen, cat. #MPK1096).

#### In vitro transcription (IVT) of sgRNAs

Single guide RNAs (sgRNAs) were in vitro transcribed from double-stranded DNA templates as previously described by Gagnon *et al.* (PMID: 24873830). In brief, 60-mer DNA oligos harbouring an 18–20-mer protospacer sequence flanked 5′ (upstream) by a T7 promoter sequence (TAATACGACTCACTATAGG) and 3′ (downstream) by part of the conserved tracrRNA domain sequence (GTTTTAGAGCTAGAAATAGCAA) was annealed to a universal 80-mer oligo (AAAAGCACCGACTCGGTGCCACTTTTTCAAGTTGATAACGGACTAGCCTTATTTTAACTTGCTATTTCTAGCTCTAAAAC) harbouring the remainder of the tracrRNA and gap-filled using T4 DNA polymerase (NEB). The resulting double-stranded DNA template was column purified using a DNA Clean & Concentrator kit (Zymo Research, cat. #D4013) and 500 ng used in a 30 μl IVT reaction as per manufacturer's protocol (HiScribe T7 High Yield RNA Synthesis Kit, NEB, cat. #E2040S). After 4 h incubation at 37ºC, remaining DNA template was degraded by adding 2 μl DNAseI and 18 μl water to the IVT reaction. Finally, sgRNAs were purified using a MEGAclear Transcription Clean-Up Kit (Thermo Fisher Scientific, cat. # AM1908) according to the manufacturer's instructions and eluted in 25 ul nuclease-free water. Guide RNA concentration and purity was measured using a NanoDrop 1000 spectrophotometer (Thermo Fisher Scientific).

#### Generation of knockout cell lines

Cells (2 × 10^5^) were nucleofected with 5 μg Cas9 protein (IDT) and 1 μg sgRNA in a total volume of 10 μl ‘buffer R’ using the Neon Transfection System (Invitrogen, cat. #MPK5000) recommended protocols for RKO and SH-SY5Y cell lines.

For each gene target, multiple guide sequences were used to generate the sgRNAs as follows:

gRNA_SPINT1_in1 TAATACGACTCACTATAGGACCTTCTGTGAGTCCCCCATGTTTTAGAGCTAGAAATAGCAA

gRNA_SPINT1_in2 TAATACGACTCACTATAGGAACTCTCTAGAGAGCCTCTGGTTTTAGAGCTAGAAATAGCAA

gRNA_BCL2 left AGCCTGATACCATGGTCCAAATGGGCTCAGGATGGGAGGC, gRNA BCL2 right TAAAAAATCCCTGGCTCCACTGAAGACTACAGTTGGACGT

gRNA_EGFR left CAGGGCCCAGCATCATGGGT

gRNA EGFR right GACGGAGTTCAGGGCTGCTC

#### Screening of mutant cell line

Cells subjected to nucleofection were sorted into single cells onto 96-well plates for recovery. Genomic DNA was extracted from single cell pellets by phenol:chloroform:isoamy alcohol (25:24:1) extraction followed by PCR using Phusion High-Fidelity PCR Master Mix (NEB) (SPINT1 forward primer: CAGCCACTTTTCGTTCCTGC; SPINT1 reverse primer: AACCATGCTACCTTAGGGTTTAT; BCL2 forward primer TTCCTGTCCCTCCAAGGTAAC; BCL2 reverse primer CAGGATTATTTCCCTGAACGCTT; EGFR forward primer AGGCAGCAATGGAGTCCTTC; EGFR reverse primer TGACTCACCGTAGCTCCAGA). PCR products were subjected to agarose gel electrophoresis and visualized by UV transilluminator. BCL2 KO cells were further screened by restriction enzyme BtsCI digestion by incubating the The PCR product produced using the primers listed above was incubated with 20 U of the enzyme at 37°C for 2 h and then subjected to agarose gel electrophoresis.

### Luciferase assay

#### psiCheck2 EGFR target reporters

To construct EGFR and SPINT1 target reporters, DNA sequences corresponding to tsEGFR/SPINT1 target site (EGFR: hg38, chr7:55192375–55192624; SPINT1: hg38, chr15:40,848,088–40,848,337); Intron (EGFR: hg38, chr7:55077451–55077701; SPINT1: hg38, chr15:40854087–40854396); Exon (EGFR: hg38, chr7:55208864–55209135; SPINT1: hg38, chr15:40853711–40853929) and 3′-UTR (EGFR: hg38, chr7: 55207090–55207339; SPINT1: hg38, chr15:40857894–40858143) were synthesized and inserted into the XhoI and NotI sites of the psiCHECK2 plasmid (Promega). Correct insertion of the sequences was confirmed by Sanger sequencing.

#### Transfection and Luciferase Assay

tsRNA-mediated silencing has been assessed by Dual-Luciferase Reporter Assay System (Promega). HeLa cells were plated in 24-well tissue culture plates at density of 1 × 10^5^ cells per well 24 h before transfection. 1 μg of psiCHECK2 (tsEGFR target site, Intron, Exon and 3′-UTR) has been transfected using Lipofectamine 2000 (Invitrogen) in accordance with the manufacturer's instructions. After 4h media was changed and 1, 0.75 and 0.5 μM of synthetic 5′-phosphorylated ssRNA tsRNA, ssRNA fully complementary (ssRNAfc) and ssRNA scrambled (scr) have been transfected using Lipofectamine 2000. The inhibitory effect of 5′phosphorylated ssRNAs on Renilla luciferase protein expression was measured on lysates collected 24 h after transfection using PHERAstar microplate reader. The ratios of Renilla luciferase (hRluc) to Photinus luciferase (hluc+) protein activities were normalized to mock transfection and the mock activity was set as 100%.

#### *In vitro* Ago2 cleavage assay

SPINT1 RNA (hg38, chr15:40847819–40848367) was prepared using HiScribe™ T7 High Yield RNA Synthesis Kit (NEB) followed by DNAse I RNAse free (NEB) treatment, purified by phenol chloroform extraction and ethanol precipitation. 50 ng of recombinant Ago2 or Ago3 protein (Active Motif) were pre-incubated with 500 nM single-stranded tsRNA in cleavage buffer (HEPES–KOH 25 mM pH 7.5; MgCl_2_ 5 mM; KCl 50 mM; DTT 0.5 mM; EDTA 0.2 mM; ATP 0.1 mM; GTP 0.02 mM; BSA 0.05 μg/μl; Ribolock 20 U; protease inhibitors EDTA free 1×) for 2 h at 37°C and followed by adding 400 ng of spint1 RNA. Cleavage reactions were quenched by TBE–urea loading buffer after 0, 3, 6 and 24 h incubation at 37°C, respectively. Cleavage products were resolved by 4% polyacrylamide 7 M urea denaturing gel at 400 V in 1× TBE buffer. For blotting cleavage products were transferred onto Hybond N+ positive charged nylon membranes (GE Healthcare) by semidry electroblotting in 0.5× TBE buffer at 300 mA for 60 min. After UV crosslinking at 120 mJ/cm^2^ in a Stratalinker UV crosslinker, the membrane was blocked with ULTRAhyb Hybridization Buffer (Invitrogen) for 30 min at 45°C and then incubated with 5′-32P labelled probe (5′-TGATAGATATTGCCCAACTACC) overnight at 45°C. The blot was washed twice with 0.1× SSC at 45°C for 15 min. The blot was exposed to Hyperfilm (GE Healthcare) for 2 days at −70°C.

#### *In vitro* qPCR rAgo2 cleavage assay

SPINT1 cleavage reaction was performed as described above with 200 ng of recombinant Ago2, 400 ng of SPINT1 and 500 nM tsSPINT1 in reaction buffer (HEPES-KOH 25 mM pH 7.5; MgCl_2_ 5 mM; KCl 50 mM; DTT 0.5 mM; EDTA 0.2 mM; ATP 0.1 mM; GTP 0.02mM; BSA 0.05 μg/μl; Ribolock 20 U; protease inhibitors EDTA free 1×) and incubated for 18 h at 37°C. 2 μl of the reaction were used as reverse transcriptase (Superscript III) template using primer R1 (GATATTGCCCAACTACCCTCC). qPCR was performed using primers F1 (TAGCCTGTCTGTCTGCTAGG) and R1.

#### *In vitro* qPCR FLAG-Ago2 cleavage assay

Approximately 8 × 10^6^ recombinant HEK293T-REx cell lines with integrated expression cassettes for FLAG-Ago2 wt and FLAg-Ago2^D669A^ mutant were lysed with lysis buffer (50 mM Tris–HCl pH 7.6, 150 mM NaCl, 3 mM MgCl_2_, 0.5% Triton X-100, 5% glycerol and 1× protease inhibitor) for 45 min at 4°C and sonicated for 10 s at 10 μm and the lysate was cleared with centrifugation. Cleared lysate was incubated with pre-equilibrated Anti-FLAG M2 beads (Sigma) for at least 2 h at 4°C. Beads were washed twice with 1× TBS and pre-incubated with 1 μM tsSPINT1 for 2 hours, 1 μg SPINT1 in reaction buffer and incubated at 37°C and shaken at 1200 rpm for 18 h. RNA was isolated with phenol/chloroform extraction and equimolar amounts of RNA were used as template for reverse transcription with primer R1. qRT-PCR was performed using primers F1 and R1.

#### Statistical analysis of qPCR data

qPCR data were analysed using raw Ct values. When comparing two conditions, data were subjected to Shapiro–Wilk test and F test to assess for normality and equal variance; if they follow a normal distribution and have the same variance, unpaired *t*-test (one-tailed) was performed to test for significant difference (*P*-value < 0.05 is considered as significant). If data do not follow a normal distribution, unpaired Mann Whitney test (one-tailed) was used instead. For comparison of two or more conditions, one-way ANOVA was performed followed by Tukey multiple comparisons test.

### Bioinformatic analysis

#### sRNA-seq and PAR-CLIP

sRNA-seq data for Dicer knockdown and scrambled shRNA control (3 reps) and PAR-CLIP data for AGO 1, 2, 3 and 4 (24) and Dicer (25) (three reps) were adapter trimmed using cutadapt 1.8.3 with –minimum-length 18. Further sequences consisting of partial adapters were removed using a custom written perl script. We have then used the pipeline SPORTS1.1 (26) to map the reads with up to three mismatches. Read abundances were extracted from the SPORTS1.1 ‘summary.txt’-files. For sRNA data, log_2_ fold changes were computed by using DESeq2 (27), where each unique tsRNA sequence was defined as a feature, whilst only including features supported by more than one read.

PAR-CLIP data was mapped to tRNAs ±7 nt and tRNA + CCA using bowtie –S –v3 –all –best –strata (24). For PAR-CLIP, only sequences between 18 nt and 22 nt in length were considered for analysis. Reads were equalized to their genomic sequence.

For PAR-CLIP reads occurring 25 times in all AGO sets and 323, 41 or 19 times in either Dicer rep1, rep2 or rep3 sets respectively were considered further (cut-offs were due to different library sizes and distribution of read occurrence). These were considered to be tsRNAs.

#### chromRNA-seq

cDNA and ncRNA sequence data was downloaded from Ensembl version 89 (28) and a kallisto (v0.43.1) index was built. Read counts for RNA-seq data were generated using kallisto with the following options: *–rf-stranded -b 100 -t 5*.

#### Differential gene expression

Differentially expressed genes were determined with DESeq2 (27). For mRNA-seq genes with the FDR adjusted *P* <0.001 were considered as significantly differentially expressed. For chrRNA-seq, genes with the adjusted *P* <0.005 for shDicer and shAgo2 were considered as significantly differentially expressed. Due to the low number of changing genes, a less stringent criterion of adjusted *P* <0.05 was used in shDrosha samples.

#### Target gene prediction

tsRNA targets were predicted by running miRanda 3.3a (29) with the parameters -sc 150 -en -30-quiet against significantly upregulated genes determined from mRNA-Seq. The same analysis was repeated for genes significantly upregulated in shDicer and shAgo2 in chrRNA-seq.

#### Disease association heatmap

We downloaded the table for all gene-disease associations as well as a curated annotation of disease and disease classes from DisGeNET (30). DisGeNET uses NCBI annotation as reference. We extracted the gene symbols and all synonyms for NCBI genes. We also extracted gene symbols for Ensembl 89 using BioMart. The gene universe was taken as the overlap of NCBI gene symbols and Ensembl 89 gene symbols and only disease and target genes with symbols in this universe were considered further. A contingency table was thus constructed and a one-sided Fisher exact test was employed to assess the significance of the observation.

Heatmaps were plotted in R using the p heatmap package by constructing a binary matrix for target-gene–disease/disease class associations. This matrix was ordered first by column sums (genes), then by row sums (diseases).

#### Conservation analysis

multiz7way scores for all target sites were extracted from UCSC. Target sequence scores were identified from sequence chr, start_coordinate and length. If a target sequence was not identifiable from these metrics, it was discarded from the conservation analysis.

Mouse homologs for the 1758 human tsRNA target genes were extracted using the getLDS() function of biomaRt (31) in R. 1263 homologous mouse genes were identified.

The tsRNAs targeting a human gene were target mapped with miRanda against the mouse homologs with default parameters, implying that no minimal binding energy was imposed and the miRanda alignment score threshold was set at 140. Of note, the tsRNA target prediction for human genes had been done with maximal energy threshold of –30 kcal/mol and miRanda score of 150 (see above).

For each human target gene, only the tsRNAs targeting it were considered for target prediction to the mouse homolog. In turn that means that each tsRNA targeting a set of human genes was run only against the mouse homologs of that particular set of genes.

Each human gene h_g_ is targeted by a set (t_1_,…,t_k_)_g_ of tsRNAs. We considered how many of these tsRNAs targets the mouse homolog m as a fraction of the set size |(t_1_,…,t_k_)_g_ |. Of 1263 considered human-mouse homologs, 1012 mouse genes are targeted by 90% of the same tsRNAs. In other terms, of 1263 hg–mg pairs, there are 1012 mg targeted by 0.9*|(t_1_,…,t_k_)_g_ | of the elements in (t_1_,…,t_k_)_g_.

## RESULTS

### tRNAs are processed by Dicer into small RNAs

**Figure 1.**
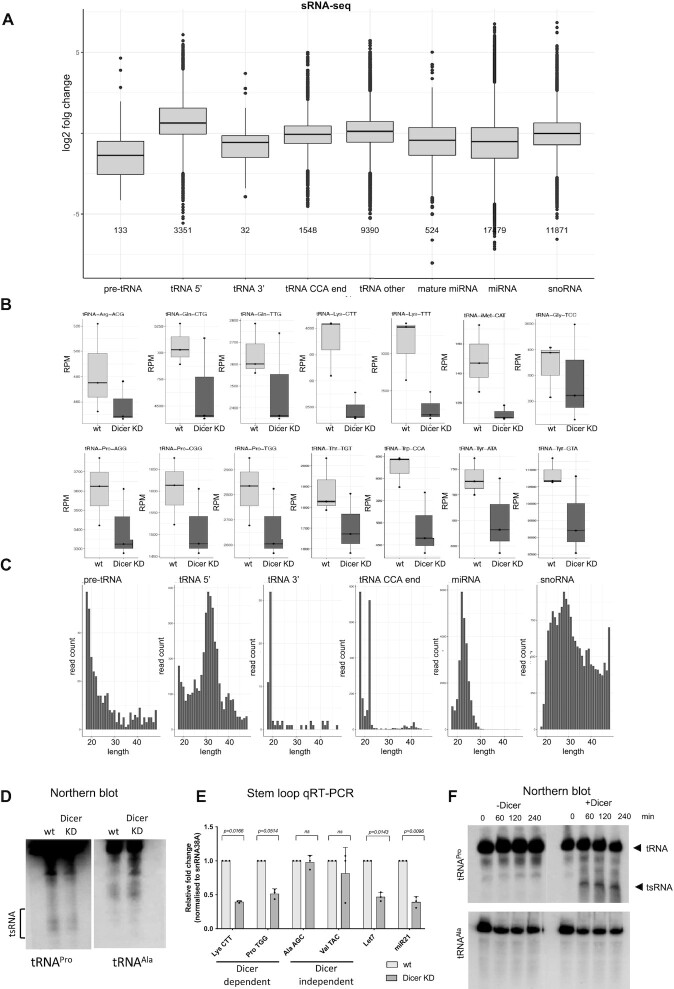
tRNAs are processed by Dicer into tsRNA. (**A**) Box plots showing the log2 fold change of various tsRNA types, miRNA and snoRNA upon Dicer knockdown. (**B**) Box plots showing Dicer dependent tsRNA derived from various tRNA isotypes (**C**). Bar charts representing the length distribution of various tsRNA types based on sequence length, in nucleotides (nt). (**D**). Images of northern blot showing signals of tRNA and corresponding tRFs for Pro, and Ala in wild type HEK293T and an inducible Dicer knockdown (shDicer) cell line. (**E**) Stem loop qRT-PCR showing levels of selected tsRNAs and miRNAs fragments isolated from wt and Dicer KD cells. The values are derived from three biological repeats and each in the bar chart corresponds to average value of two technical repeats. (**F**) Northern blot images showing signals of tRNA^Pro^ and its corresponding tsRNA (tRF-3^Pro^), and tRNA^Ala^ at different time points after the addition of FLAG-tagged Dicer or TAP-tagged Dicer.

We first examined whether the depletion of Dicer affects cellular levels of tsRNAs. We employed a HEK293T cell line which contains a stably integrated doxycycline-inducible cassette expressing short hairpin (sh)RNA against *DICER* mRNA (shDicer). Small RNA sequencing (sRNA-seq) comparing levels of tsRNAs, micro(mi)RNAs and small nucleolar (sno)RNAs between wild type (wt) and shDicer cells revealed that tsRNA and miRNA, but not snoRNA levels were significantly reduced upon Dicer knockdown (Figure 1A). We used pipeline SPORTS1.1 (26), specifically designed for analysing sRNA data mapping to tRNAs that considers mature tRNAs, pre-tRNAs (this category does not includes tRNA 3′end reads, but includes reads that may be mapping to tRNA introns), and tRNAs with their CCA ends as potential origins for tsRNA biogenesis. We show that tsRNA derived from pre-tRNA, tRNA 3′ends, mature miRNA and miRNA are particularly Dicer dependent. However, we detected Dicer dependent small fragments also derived from tRNA 5′ends, tRNA CCA ends and middle parts of tRNA (tRNA other: this category includes reads that do not map any of the previous categories but still map to tRNA, for example mature tRNA body) (Figure 1A). The most abundant short tsRNA fragments (length 18–22nt) were derived from tRNA CCA ends, whilst longer tsRNAs (length 22nt+) originated from middle parts of tRNA (Supplementary Figure S1A–C). Next, we found that certain tRNA isotypes serve more likely as Dicer substrates than the others (Figure 1B and Supplementary Figure S1D). Analysis of tsRNAs length revealed that Dicer dependent tsRNA derived from pre-tRNA and tRNA 3′ends are 18–22nt long, while tRNA 5′end derived tsRNA are longer (Figure 1C and Supplementary Figure S2A and B and Supplementary file 1). Most tRNA isotypes would result in short (18–22nt) tsRNA fragments. However, we also identified some tRNA exceptions, in particular tRNA^Gly^, tRNA^Glu^, tRNA^His^, tRNA^Lys^, tRNA^Asp^ and tRNA^Asn^ that resulted in longer fragments (Supplementary Figure S2C). To illustrate how many sequences, fall into these categories, we use a cumulative length distribution plot to show that 68% of tsRNA are up to 22nt long and 72% are up to 25nt long (Supplementary Figure S2D). For comparison, the length distribution of miRNA showed most fragments around 22nt long as expected and snoRNA fragments showed greater length variation (Figure 1C and Supplementary Figure S2A and B). Finally, we analysed whether some tRNA are more likely to be processed into tsRNA. In order to do that we have analysed the expression of tRNA and the abundance of tsRNAs derived from it. As expected we observed moderate positive correlation (Spearman's ρ = 0.32, p = 0.025) between tRNA expression and corresponding tsRNA abundance with only few exceptions (Supplementary Figure S2E).

We then selected two tsRNA candidates for northern blot and the analysis confirmed that tsRNA derived from tRNA^Pro^ were reduced upon Dicer knockdown whilst tsRNA derived from tRNA^Ala^ were hardly detectable (Figure 1D and Supplementary Figure S3A). In order to quantify tsRNA isolated from wt and Dicer KD samples, we employed stem loop qRT-PCR and showed reduced levels of tsRNA fragments derived from tRNA^Pro^, tRNA^Lys^ and Dicer known products miRNA Let7 and miRNA21. We used tRNA^Ala^ and tRNA^Val^ as negative, Dicer independent, controls (Figure 1E and Supplementary Figure S3B). To demonstrate that Dicer directly cleaves tRNA, we performed an *in vitro* Dicer cleavage assay. Incubation with Dicer led to reduced levels of tRNA^Arg^ and let7a precursor (pre-let7a), a well-known Dicer substrate, but did not affect levels of snRD38A (negative control) (Supplementary Figure S3C). Subsequent northern blot analyses showed the emergence of sRNA species when tRNA^Pro^, tRNA^Tyr^, tRNA^Gly^ or pre-let7a, but not tRNA^Ala^ or snRD38A, were incubated with Flag-Dicer or TAP-Dicer (Figure 1F, Supplementary Figure S3D and E).

**Figure 2.**
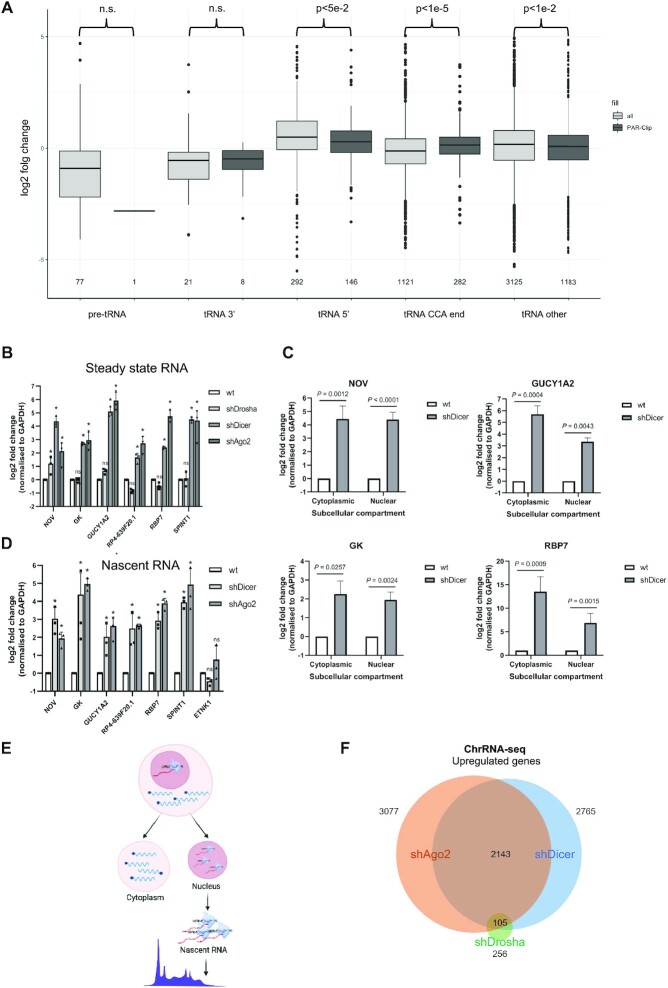
tsRNA target genes in nucleus. (**A**) Box plots showing the log2 fold change of various Dicer dependent tsRNA types present in Dicer PAR-CLIP data. (**B**) Bar chart showing the log_2_ fold change of steady-state levels of six selected predicted tsRNA target genes upon Drosha, Dicer and Ago2 knockdown. (**C**) Bar charts showing the log2 fold change of the steady-state levels of four selected predicted target genes, measured by qRT-PCR, in the cytoplasm and nucleus upon Dicer knockdown. (**D**) Bar chart showing the log_2_ fold change of the nascent RNA levels of six selected target genes upon Dicer and Ago2 knockdown, with *ETNK1* as negative control. (**E**) Diagram summarizing ChromRNA-seq approach. (**F**) Venn diagram displaying the overlap between upregulated genes upon Dicer (*P* < 0.005), Ago2 (*P* < 0.005) and Drosha (*P*< 0.05) knockdown detected in chrRNA-seq.

Next, we analysed whether tsRNAs that are bound to Dicer proteins are also Dicer dependent. To this end, we employed Dicer PAR-CLIP data and intersected them with sRNA-seq data from wt and Dicer knockdown cells. We show that 18–22nt long tsRNAs originated from pre-tRNA, tRNA 3′ends and mature tRNA identified in Dicer PAR CLIPs were significantly reduced upon Dicer depletion (Figure 2A). Altogether these data suggest that Dicer specifically binds and cleaves certain tRNAs and is therefore involved in the biogenesis of a subset of tsRNAs.

### Bioinformatic prediction of Dicer dependent tsRNA target genes

Next, we took a bioinformatic approach to explore the role of Dicer-dependent tsRNAs in gene silencing. Using Dicer and Ago PAR-CLIP data, we isolated 18- to 22-nt tsRNAs that were bound to Dicer, Ago1, Ago2, Ago3 and Ago4. Interestingly, when grouping the unique tsRNA sequences into highly similar ones (one tsRNA sequence is the same as the other but one nucleotide longer), we found that most tsRNA sequences were identified in the Ago2 PAR-CLIP data (161 out of 192 sequences), suggesting an association between tsRNAs and Ago2 (Supplementary Figure S3F). To predict tsRNAs target genes, we mapped Dicer- and Ago- bound tsRNAs to the genomic sequences of upregulated genes in Dicer knockdown cells (from RNA-seq data comparing gene expression in wt HEK293T and shDicer cells) using miRanda, a bioinformatic tool for miRNA target prediction (Supplementary Figure S3G). As a result, we obtained a list of genes that were predicted to be targeted by Dicer- and Ago-bound tsRNAs and their exact predicted target sites. We then confirmed the upregulation of randomly selected target genes identified in Dicer and Ago2 knockdown (shAgo2) conditions, whilst the expression of these candidates remained unaffected in Drosha knockdown (DroshaKD) (Figure 2B, Supplementary Figure S3H), indicating that they are not regulated by a miRNA-dependent mechanism (32). To investigate the upregulation in more detail, we performed subcellular fractionation followed by qRT-PCR and found that the upregulation of four selected target genes occurred, surprisingly, in both the cytoplasm and the nucleus (Figure 2C and Supplementary Figure 4C). This argues against a role for PTGS, which should only lead to an increase of transcript levels in the cytoplasm. Next, we measured nascent RNA levels of selected target genes and observed their significant upregulation in shDicer and shAgo2 cells, but not in Drosha KD (Figure 2D and Supplementary Figure 4A, B). If TGS would be involved, RNA polymerase II (RNAPII) levels at the predicted target genes should increase in the absence of Dicer. However, ChIP analyses showed that amount of total and active RNAPII interacting with the promoter, exon and 3′ untranslated regions (UTR) of three selected target genes remained unchanged upon Dicer depletion. In contrast, total and active RNAPII was significantly upregulated at known TGS loci: CCR5, TBCEL and EF1A (33–35) upon Dicer depletion (Supplementary Figure S5A). Western blot analysis showed that the overall protein levels of active RNAPII were unaffected in shDicer cells (Supplementary Figure S5B), suggesting that the observed upregulated gene expression of target genes was not caused by a global increase of transcription. Finally, we tested whether we can detect any TGS over tsRNA target genes and known TGS loci. We showed that repressive mark dimethylated H3 at lysine 9 (H3K9me2) was not observed at target genes, whilst it was significantly enriched at known TGS loci and reduced upon Dicer depletion (Supplementary Figure S5C). These results point towards a novel gene silencing mechanism that is distinct from TGS and PTGS. We hypothesized that Dicer-dependent tsRNAs can lead to gene silencing by targeting nuclear RNA without affecting the levels of transcription.

Unaffected RNAPII levels upon Dicer knockdown prevented us from using NET-, GRO- or PRO-seq approaches to further examine this gene silencing mechanism genome-wide. Therefore, we performed chromatin-associated RNA sequencing (chrRNA-seq) (36) to compare levels of chromatin bound RNA, as a proxy for nascent RNA (37), across wt, shDicer, shAgo2 and DroshaKD cells (Figure 2E and Supplementary Figure S6A and B). We analysed differential expression of all transcribed genes and identified an overlap of 2143 upregulated genes in Dicer and Ago2 knockdown samples, whilst only 256 genes were upregulated in DroshaKD cells (Figure 2F and Supplementary File 2).

**Figure 3.**
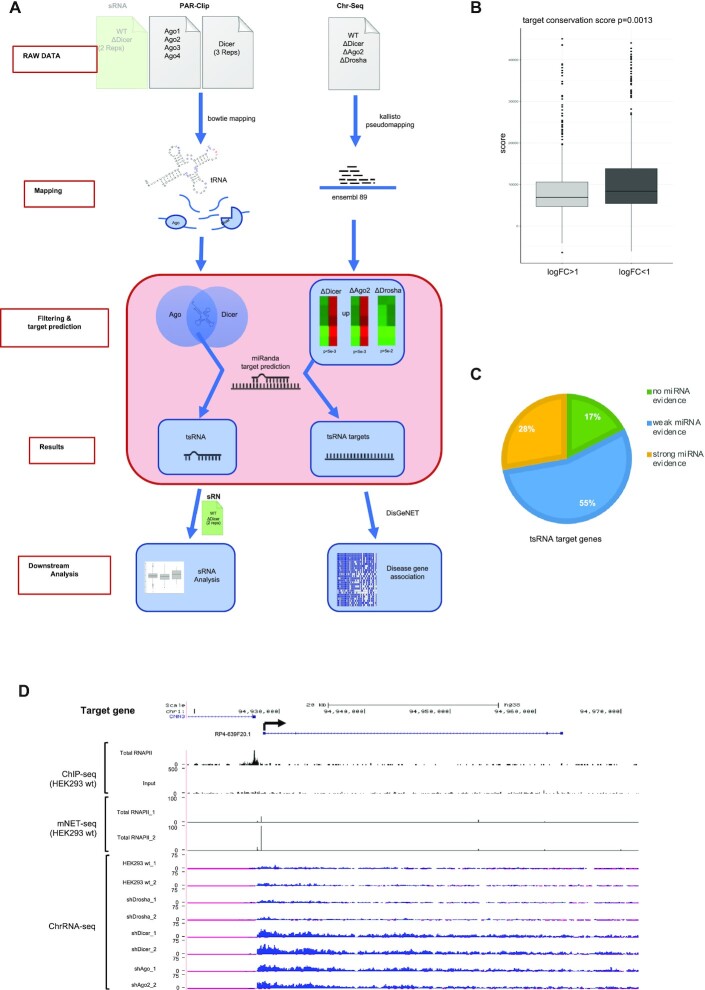
Bioinformatic prediction of tsRNA target genes. (**A**) Diagram representing the bioinformatic workflow of tsRNA target prediction using chrRNA-seq data. (**B**) Box plot showing conservation scores for Dicer dependent and independent tsRNA target genes. (**C**) Pie chart showing the breakdown of tsRNA targets based on evidence for being targeted by miRNAs. (**D**) Combined snapshot of RNA pol II ChIP-seq, mNET-seq and chrRNA-seq profiles across target gene *RP4-639F20.1* (*n = 2*). Normalized read counts are indicated.

Next, we mapped the identified tsRNAs bound by Dicer and Ago2 (from PAR-CLIP) to the genomic sequences of genes that were upregulated at the nascent RNA level in the absence of Dicer and Ago2 (chrRNA-seq) (Figure 3A). These analyses resulted in a list of 1758 predicted tsRNA targets, including protein coding genes and long non-coding RNAs. All identified genes with details of their predicted target sites for tsRNAs sequences and positions and sequences of corresponding tsRNAs are provided in the Supplementary File 3. These data suggest that tsRNAs target nuclear RNA in pathway that we called nascent RNA silencing (NRS).

The identification of target genes could result in some false positives. Therefore, we looked at the conservation scores of target genes. We have used the mutiz7way scores for all target sites, which were extracted from UCSC. We have included the conservation scores in the target table in ‘target_positions’ tab (Supplementary file 3). Restricting the target sites to the ones which are targeted by Dicer dependent tsRNA (|log_2_FC|>1 in sRNA-seq), we see a significant difference in conservation score (Figure 3B).

Since the NRS mechanism appears to be very different from miRNA driven PTGS, we analysed whether NRS target genes overlap with miRNA target genes by employing miRTarBase (38). Interestingly, most NRS targets (72%) showed no or only weak evidence (not supported by reporter assay, western blot or qPCR evidence) for being targeted by miRNAs (Figure 3C), suggesting that NRS is a distinct gene silencing mechanism which targets a subset of genes that are not preferentially targeted by miRNAs.

Next, we considered whether the genes that were upregulated following Dicer/Ago2 depletion, were transcribed in wt cells even though they generate very low levels of nascent RNA. We aligned RNAPII ChIP-sequencing (ChIP-seq) data with our chrRNA-seq data and confirmed the positive correlation between RNAPII occupancy and the expression of genes in the wt sample (Supplementary Figure S6C). Snapshots of three predicted tsRNA target genes *RP4-639F20.1*, *SPINT1* and *GK*, showed presence of RNAPII ChIP-seq and mNET-seq peaks and elevated levels of nascent RNA (shown by reads in intronic as well as exonic regions) in shDicer and shAgo2 in comparison to DroshaKD and wt samples, while levels of non-target gene *GAPDH* remained constant across all conditions (Figure 3D, Supplementary Figure S7A–C).

### Dicer dependent tsRNA target introns of genes for nascent RNA silencing

**Figure 4.**
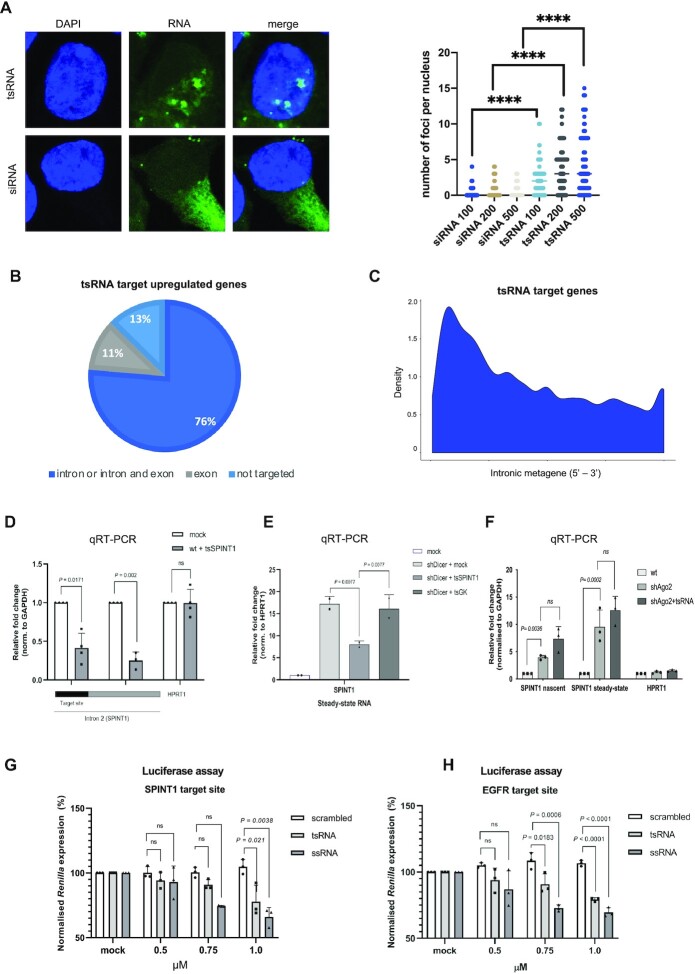
tsRNAs target introns for nascent RNA silencing. (**A**) (Left) Representative confocal images showing the localization of fluorescently labelled tsRNA and siRNA (in green) after transfection into BT549 cells. DAPI is in blue. (Right) Quantification of number of nuclear foci from confocal images. (**B**) Pie chart showing the proportion of upregulated genes that are targeted in intron or both intron and exon, targeted exclusively in exon and not targeted. (**C**) Metagene profile representing the distribution of predicted tsRNA target sites across the introns of target genes. (**D**) Bar charts showing the relative fold change of expression of the predicted tsSPINT1 (also tRF-3^Gln-CTG^) target site and the intronic site adjacent to it within intron 2 of *SPINT1* (*n = 3*), measured by qRT-PCR, with *HPRT1* as negative control. (**E**) Bar chart showing the fold change of steady-state expression of *SPINT1* upon mock transfection and transfection of tsRNA against *SPINT1*, with transfection of tsRNA against *GK* as negative control. (**F**) Bar chart showing nascent and steady state *SPINT1* RNA levels in wt, Ago2 KD and Ago2 KD transfected with tsRNA targeting *SPINT1*, with HPRT1 as negative control. (**G**) Bar chart showing the knockdown efficiency of SPINT1 intronic regions using the tsRNA, fully complementary (ssRNA) and scrambled sequences, plotted as a percentage (SD; *P = 0.019) of the normalized *Renilla* luciferase expression. The luciferase activity of the mock transfected cells was set as 100%. All tested 5′-phosphorylated ssRNA were transfected at 1 μM, 0.75 μM and 0.5 μM. Silencing activities were measured at 24 h post-transfection. (**H**) As in (G), using EGFR gene.

Our data identified Dicer dependent tsRNA mediated NRS as a nuclear mechanism. However, subcellular fractionation followed by Northern blotting showed tsRNA presence in both the cytoplasm and nucleus (Supplementary Figure S9A). This observation, however, cannot distinguish between where tsRNA are generated and the location at which they carry out their biological functions. We therefore transfected fluorescently labelled siRNAs (commercially available, targeting 3′UTR) and tsRNAs, which were predicted to target *EGFR* for silencing (tsEGFR), into the BT549 breast cancer cell line. Confocal images of the transfected cells show that the siRNA against *EGFR* only localizes in the cytoplasm, consistent with PTGS. In contrast, tsEGFR showed clear nuclear accumulation, implying a nuclear role (Figure 4A, Supplementary Figure S9B). To identify the position of tsRNA target sites, we performed further bioinformatic analysis and showed that the majority of tsRNAs were predicted to target introns (1536 out of 1758). It should be noted that most of the genes were targeted by more than one tsRNA. Only a small proportion of target genes (222 out of 1758) were targeted exclusively in exons (Figure 4B). Next, we considered whether there are any common features among genes that targeted in their introns. An intronic metagene analysis revealed that tsRNA target sites had the tendency to be positioned in early introns of the gene (Figure 4C). It should be noted that the initial introns are usually longer that the others in the gene, which may imply that they play an important role as a binding platform for regulatory elements such as tsRNA. Overall, these results imply that NRS is facilitated through tsRNA intronic targeting.

To validate tsRNAs intronic targeting, we transfected synthetic single stranded tsRNA predicted to target *SPINT1* (tsSPINT1) into wt HEK293T cells and quantified the relative fold change of the expression of intronic sites in the gene. qRT-PCR results showed significant decrease in expression levels at the predicted target site and the intronic site adjacent to it (Figure 4D). Also, in Dicer knockdown cells, in which *SPINT1* was upregulated (due to loss of endogenous tsRNAs), the transfection of tsSPINT1 led to an attenuation of upregulation of the transcript (Figure 4E). To extend these data, we also looked at SPINT1 steady state and nascent RNA levels in Ago2 KD cells and observed increased SPINT1 gene expression. Furthermore, addition of exogenous tsRNA targeting SPINT1 did not rescue elevated SPINT1 expression in Ago2 KD cells, suggesting that Ago2 plays crucial role in NRS (Figure 4F).

Next, we performed Luciferase assay to investigate the specificity of an individual tsRNA and to test various regions of the same target gene for tsRNA targeting. We used both *SPINT1* and *EGFR* (NRS target genes) sequences to clone four reporters: the intronic region containing the predicted target site, an intronic region without a target site, exon and the 3′UTR. tsRNA-mediated silencing was assessed by Dual-Luciferase Reporter Assay System (Promega). Cells were transfected with the reporters, followed by transfection of synthetic 5′-phosphorylated single stranded tsSPINT1 or tsEGFR. The silencing effect on Renilla protein expression revealed that tsRNA was only targeting the introns containing its target site for silencing, while the other reporters (an intron without the target site, an exon and 3′UTR) were not silenced. As tsRNAs is not fully complementary to its target sequence (only partially complementary like miRNA), we converted the tsSPINT1 or tsEGFR sequence into fully complementary siRNA-like molecules (ssRNA). These molecules also showed specific silencing on intronic target sites, but not on the other reporters. We also used scrambled sRNA as a control, which did not lead to any silencing (Figure 4G, H and Supplementary Figure S8A, B). Altogether, these data suggest that tsRNAs facilitate intronic NRS in a sequence-specific manner.

**Figure 5.**
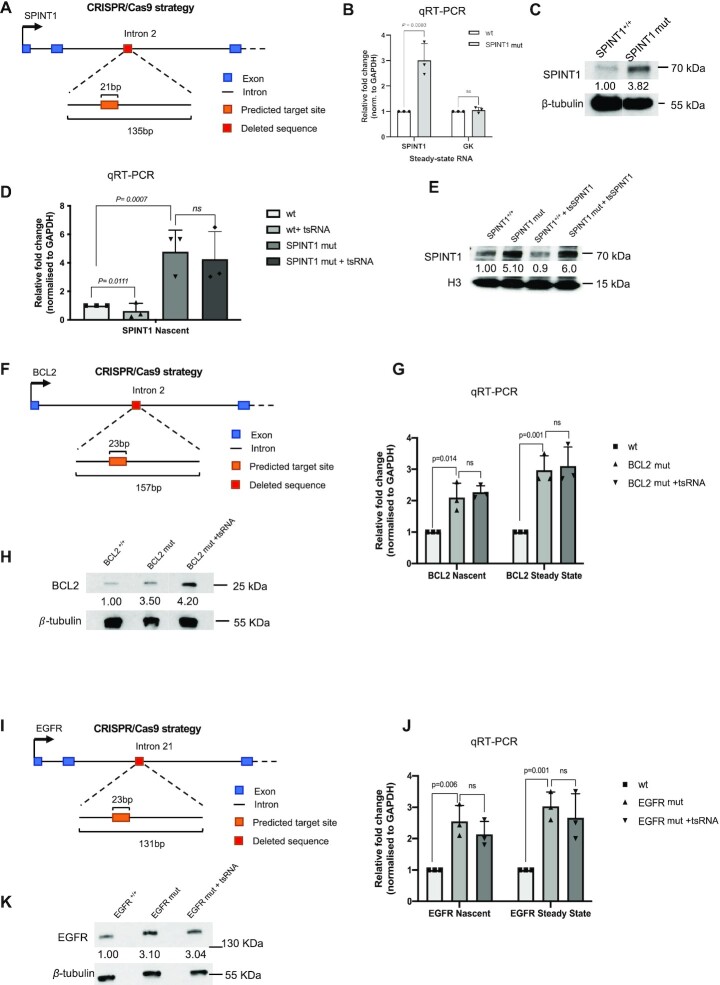
Deletion of tsRNA target site leads to increased gene expression. (**A**) Diagram representing the CRISPR/Cas9 strategy for generating a mutant HEK293T-based cell line in which the predicted target site and its flanking sequence for SPINT1 in intron 2 is removed. (**B**) Bar chart showing the fold change of expression of steady state *SPINT1* and *GK* transcripts, measured by qRT-PCR, in wild type, heterozygous SPINT1^+/–^ and homozygous SPINT1^–/–^ cells. (**C**) Western blot images showing signals of SPINT1 in wild type, and homozygous SPINT1^mut^ cells with ß-tubulin as loading control. Numbers represent quantification of the SPINT1 signal normalized to ß-tubulin. (**D**) Bar chart showing the fold change of nascent expression of *SPINT1* upon mock transfection and transfection of tsRNA against *SPINT1* in wt and SPINT1^mut^ cells. (**E**) Western blot images showing signals of SPINT1 in wild type, and homozygous SPINT1^mut^ cells mock or tsRNA transfected of with H3 as loading control. Numbers represent quantification of the SPINT1 signal normalized to H3. (**F**) Diagram representing the CRISPR/Cas9 strategy for generating a mutant HEK293T-based cell line in which the predicted target site and its flanking sequence for BCL2 in intron 2 is removed. (**G**) Bar chart showing the fold change of nascent and steady state expression of *BCL2* upon mock transfection and transfection of tsRNA against *BCL2* in wt and BCL2^mut^ cells. (**H**) Western blot images showing signals of BCL2 in wild type, and homozygous BCL2^mut^ cells mock or tsRNA transfected of with ß-tubulin as loading control. Numbers represent quantification of the SPINT1 signal normalized to ß-tubulin. (**I**) As in F for EGFR in intron 21. (**J**) As in G for EGFR. (**K**) As in H for EGFR.

To further expand the validation of intronic targeting, we generated a HEK293T-based cell line in which we deleted a 135-bp genomic segment in intron 2 *SPINT1* containing the predicted intronic target site using CRISPR-Cas9 gene editing (Figure 5A). After screening of mutant colonies, we selected a homozygous (SPINT1mut) mutant cell line for further validation (Supplementary Figure S9C, D). qRT-PCR results show an increase in *SPINT1* expression at both nascent and steady-level RNA levels in SPINT1mut cells (Figure 5B and Supplementary Figure S9E). Finally, western blotting demonstrated that SPINT1 protein levels in wt and SPINT1mut cells correlate with the transcript levels, showing increased levels of SPINT1 proteins in SPINT1mut cells (Figure 5C). To test whether SPINT1mut cells are resistant to specific tsRNASPINT1 due to lack of endogenous target site, we transfected wt and SPINT1mut cells with exogenous tsSPINT1. We observed silencing effect on nascent RNA and protein levels in wt cells but not in SPINT1mut cells (Figure 5D and E). Next, we employed the same CRISPR-Cas9 editing strategy to knock out target sites in two other target genes EGFR and BCL2 (Supplementary Figure S9F and G). Again, in both cases the expression of the target gene was increased in mutant cells on nascent, steady state and protein levels. Furthermore, these mutants were resistant to exogenous tsRNAs (Figure 5F–K). These data confirm the specificity of tsRNAs to their predicted target sites in the introns of their target genes.

### Nascent RNA silencing is mediated by Ago2 cleavage activity

**Figure 6.**
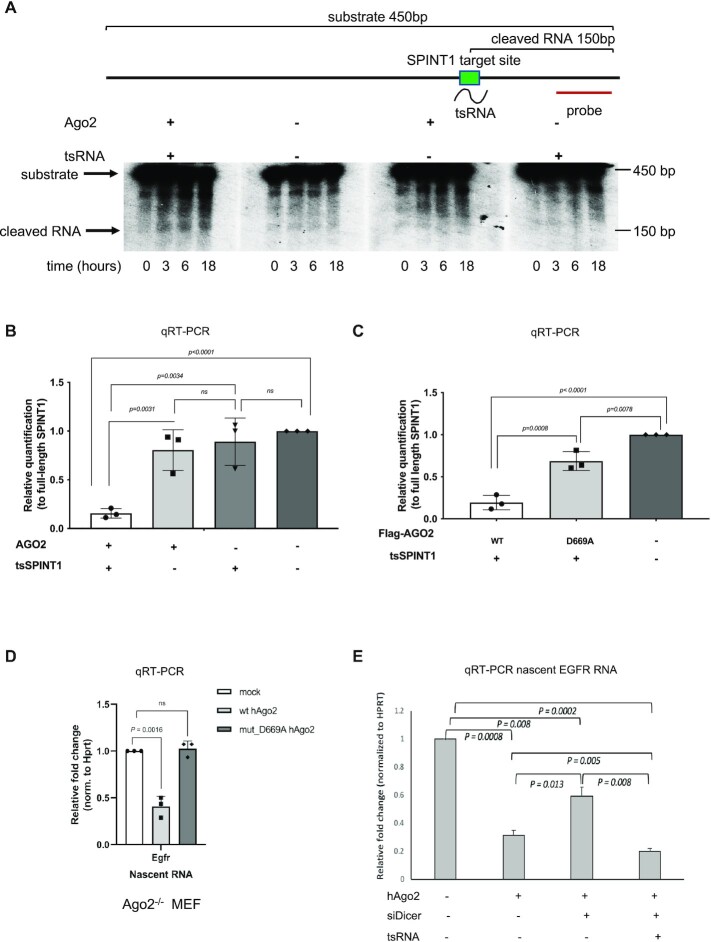
Nascent RNA silencing is mediated by Ago2 cleavage. (**A**) Images of northern blots showing signals of the tested RNA substrate, which was derived from the genomic sequences of SPINT1 containing its predicted target site in intron 2 and flanking sequences, and the resulting cleaved RNA at different time points after the addition of components indicated above each image. The experimental strategy is illustrated in the diagram on top. (**B**) Bar chart showing the RNA levels of SPINT1 intron substrate containing tsRNA target site, in presence or absence of recombinant Ago2 and tsRNA targeting SPINT1. (**C**) As in (B), in presence of Flag tagged wt and Ago2^D669A^ mutant. (**D**) Bar chart showing the fold change of mouse nascent *Egfr* transcripts, measured by qRT-PCR, upon mock transfection and transfection of plasmids expressing wild type human Ago2 (wt hAgo2) and mutant form of human Ago2 D669A (hAgo2^D669A^) in MEFs. (**E**) Bar chart showing mouse nascent EGFR levels in wt MEF ^Ago2-/-^ transfected with hAgo2, siRNA targeting Dicer and tsRNA targeting human EGFR.

Previously, we observed that tsRNAs were bound to Ago2 and predicted tsRNA target genes were upregulated upon Ago2 knockdown. We therefore hypothesized that Ago2 is an important player in NRS. First, we showed Ago2 binding to a selected tsRNA, originated from tRNA^Arg^, by immunoprecipitation of Ago2 followed by northern blotting (Supplementary Figure S10A). If Ago2 is guided to nascent RNA by tsRNA, then its proximity to the chromatin should be transcription dependent. Indeed, we demonstrated that transcriptional inhibition by α-Amanitin led to reduced levels of Ago2 on chromatin (Supplementary Figure S10B). These observations led us to test whether Ago2 cleaves RNA substrates in the presence of tsRNA *in vitro*. We incubated recombinant Ago2 with tsRNA and with *in vitro* transcribed RNA substrate (450 bp), derived from the genomic sequences of SPINT1 intron containing the predicted target site, in presence of a cocktail of ATP and Mg^2+^. Northern blotting showed the accumulation of sRNA (150 bp), corresponding to sRNA yielded from specific cleavage at the predicted target site. The absence of either tsRNA or Ago2 prevented cleavage of the substrate RNA (Figure 6A and Supplementary Figure S10C). These data suggest that Ago2 can indeed cleave substrate RNA, not only in the presence of fully complementary sRNA (siRNA), but also in the presence of partially complementary sRNA (tsRNA). To measure the Ago2 cleavage activity quantitatively, we designed qRT-PCR experiment using *in vitro* transcribed SPINT1 RNA substrate containing tsRNA target site and added recombinant Ago2 in presence or absence of tsSPINT1. The cleavage activity was determined by the amount of the substrate (Supplementary Figure S10D). Indeed, we showed significant cleavage of the RNA substrate when Ago2 was added together with tsRNA. This cleavage activity was not observed in the absence of either tsRNA or Ago2 (Figure 6B). Next, we performed this experiment with FLAG-tagged Ago2 wt and cleavage impaired mutant Ago2^D669A^. We observed efficient cleavage of the RNA substrate in presence of Ago2wt but not in Ago2^D669A^ mutant. It should be noted that partial cleavage detected in presence of Ago2^D669A^ mutant was most likely caused by unspecific nucleases present in Ago2 pull-down fractions (Figure 6C).

Next, we identified 1263 homologous mouse genes for the 1758 human tsRNA predicted target genes we generated. Of the 1263 mouse homologs, 1243 were predicted to be targeted by at least one tsRNA, which was predicted to the equivalent human gene. 395 human tsRNAs (out of 395) were predicted to target at least one of the corresponding homologs in mice. We also investigated whether there is any difference between tsRNAs that target human genes and their mouse homologs. Notably, of 1012 mouse genes (of 1263), each was targeted by 90% of the same tsRNAs as its human homolog (Supplementary Figure S11A and B). These analyses imply that NRS is an evolutionarily conserved mechanism; this is expected as tRNA are conserved across many species. This also led us to employ an Ago2 knockout (Ago^–/–^) mouse embryonic fibroblast (MEF) cell line (39) to test the *in vivo* Ago2 cleavage in NRS. We transfected Ago2^–/–^ MEF cells with plasmids expressing either wt or hAgo2^D669A^, a mutant form of hAgo2 which lacks endonuclease activity, but can still bind sRNA and lead to translational repression (1). First, we checked the expression of both hAgo2 plasmids in these MEF cells by qRT-PCR and showed that they were both expressed (Supplementary Figure S11C). Next, we showed that the expression of wt hAgo2 restored the NRS effect on mouse *Egfr* at the nascent level. Interestingly, the expression of hAgo2^D669A^ did not downregulate *Egfr* nascent RNA levels, further supporting the model where the ‘slicer’ activity of Ago2 is crucial for NRS (Figure 6D). Finally, western blot analysis showed that transfection of both wt and hAgo2^D669A^ can decrease *Egfr* protein levels, indicating that whilst wt hAgo2 at least partially employs NRS, the mutant hAgo2^D669A^ might have restored miRNA mediated PTGS (Supplementary Figure S11D). Similarly, we tested expression of another target gene BCL2 and observed that its expression was also significantly decreased in presence of hAgo2 in transfected Ago2^–/–^ MEF cells (Supplementary Figure S11E). Finally, we extended these analyses and compared the expression of mouse EGFR in Ago2^–/–^ MEF cells, transfected with hAgo2, in either wt or Dicer KD background in presence or absence of human tsRNA targeting EGFR. We observed that Dicer KD partially affected Ago2 mediated NRS of mouse EGFR. However, this was restored in the presence of exogenous human tsRNA targeting EGFR (Figure 6E). These data support the conclusion that Ago2 endonuclease activity is required for NRS.

### Nascent RNA silencing targets disease associated genes

**Figure 7.**
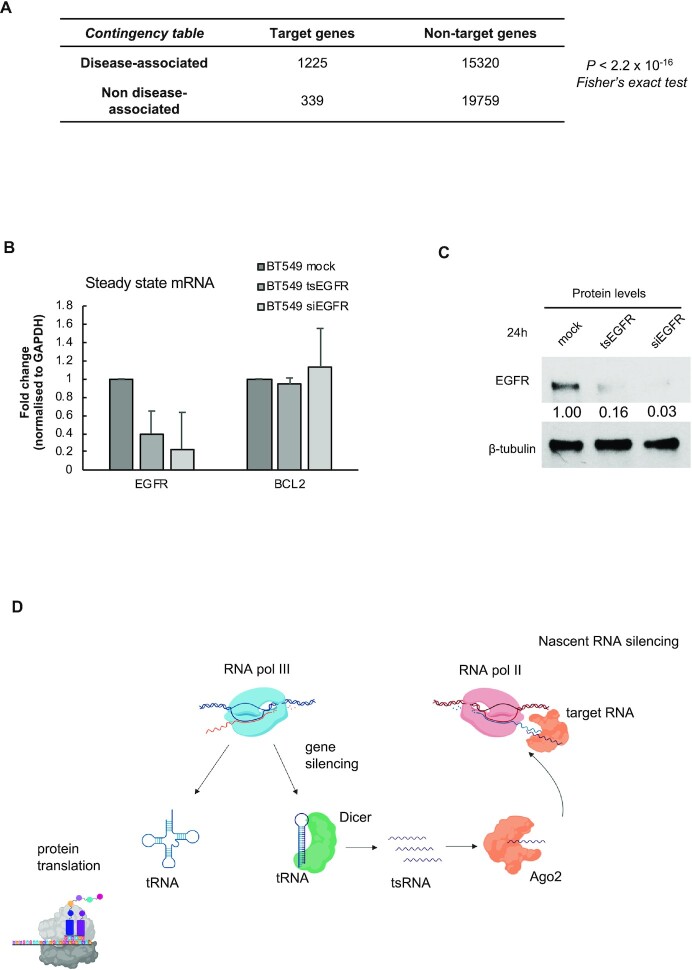
Nascent RNA silencing targets disease-associated genes. (**A**) Contingency table summarizing disease association of target and non-target genes (*P <* 2.2e–16; Fisher's exact test). (**B**) Bar chart showing the fold change of steady-state *EGFR* transcripts, measured by qRT-PCR, upon transfection of synthetic tsRNA and siRNA against *EGFR*, with *BCL2* as negative control. (**C**) Western blot image showing signals of EGFR upon transfection of synthetic tsRNA and siRNA against *EGFR*, with ß-tubulin as loading control. (**D**) Model summarizing nuclear RNA silencing. Dicer recognizes transcribed tRNA and processes it into functional tsRNA. This is loaded onto Ago2 and translocated into nucleus where they target nascent RNA for degradation through Ago2 cleavage activity.

Next, we explored the biological importance of tsRNA-mediated NRS. Using DisGeNET, a database for human disease-related genes, we found that tsRNAs target genes were significantly disease-associated, relative to non-target genes (Figure 7A). Furthermore, we identified association with at least one disease for 1210 target genes (out of 1542) (Supplementary Figure S12 and S13). This finding underscores the biological importance of the NRS mechanism.

### Synthetic tsRNAs silence expression of target genes

We considered whether we could use synthetic tsRNA to knock down proto-oncogenic genes in cancer cell lines. First, we transfected synthetic single-stranded tsRNA targeting *EGFR* (tsEGFR) into BT549 breast cancer cells in parallel with commercially available double-stranded siRNA against *EGFR* (siEGFR) and measured the change of *EGFR* expression by qRT-PCR. Surprisingly, transfection of tsEGFR resulted in the repression of *EGFR* to a degree similar to that of siRNA, whilst the expression of BCL2 (non-target for this particular tsRNA) remained unchanged (Figure 7B). We extended these results further by comparing the specificity of individual tsRNA. We compared the NRS effects on EGFR expression using the tsEGFR and tsSPINT1 and showed that only the former silenced EGFR expression at both steady state and nascent levels (Supplementary Figure S14A and B). Also, western blot analysis confirmed the repression of EGFR proteins by tsRNA and siRNA (Figure 7C). Furthermore, we demonstrated similar NRS effects at both steady-state and nascent transcription levels using synthetic tsRNA against a non-coding RNA, *LINC00665* in A549 lung cancer cells (Supplementary Figure S14C and D). It should be noted that many long non-coding RNAs are nuclear and therefore very difficult to target by siRNA, whilst tsRNA can lead to their silencing in the nucleus. Finally, transfection of tsRNA against *BCL2*, an anti-apoptotic factor into MCF7 breast cancer cells resulted in decreased levels of BCL2 protein. This in turn leads to increased levels of cleaved Caspase-9, an apoptotic signal (Supplementary Figure S14E and F).

## DISCUSSION

### Dicer dependent tsRNA biogenesis

Regulatory sRNAs, particularly miRNA, siRNA and PIWI-associating (pi)RNA have been identified and extensively studied over the past two decades. Although tsRNAs were at first considered as products of random degradation, improved sequencing techniques have resulted in the discovery of this sRNA species in many organisms. The biogenesis of tsRNAs, however, remains a matter of debate. There are reports showing that individual tRF-3s and tRF-5s are Dicer dependent however, meta-analysis of published bioinformatic data revealed most tRF-3s and tRF-5s are present in Dicer knockout mouse stem cells (15). We identified a subset of human tsRNAs, derived from pre-tRNA, tRNA 3′ends and mature tRNA, that are indeed dependent upon Dicer. It is possible that using various cell lines, tissues or perhaps species might result in some differences.

Previously Dicer has been shown to be binding to structural RNAs, such as tRNAs, snoRNAs and vault RNAs, by PAR-CLIP; the depletion of Dicer also led to a slight increase in tRNA levels, measured by qPCR using primers that amplify the full-length RNA (25). Other reports also suggested that tRNAs might fold into shRNA-like structures that could be processed by Dicer (5,13,40). Based on these findings, we speculate that a proportion of tRNAs are folded into shRNA and serve as a source of tsRNA, which act as regulators of gene expression.

We observed that Dicer depletion led to destabilization of tsRNAs, including those derived from pre-tRNA, tRNA 3′ends and mature tRNA, using northern blotting and next generation sequencing. However, it is possible that the decrease of tsRNAs is caused by an indirect effect of Dicer knockdown. To circumvent this, FLAG-Dicer was shown to cleave tRNA^Pro^ and other tRNAs *in vitro*, generating tsRNA. Importantly, our data echoes the report by Cole *et al.* in which the authors observed marked decrease of a tsRNA derived from tRNA^Gln^ using a different Dicer knockdown strategies and also detected *in vitro* generation of tsRNA by Dicer (11).

### tsRNA mediated nascent RNA silencing

Here, we propose a novel gene silencing mechanism for Dicer-dependent tsRNAs. In NRS, tsRNAs guide Ago2-containing silencing complexes to target nascent transcripts for direct cleavage in a sequence specific manner. This hypothetical model was built upon the evidence that tsRNA target genes were upregulated upon Dicer and Ago2 knockdown, but the transcription of these genes was unaltered. This was later validated by the silencing of target genes by transfection of synthetic tsRNAs and the upregulation of target gene upon deletion of predicted target sites. However, we also identified genes that were downregulated upon Dicer and Ago2 knockdown. We also predicted tsRNA target sites in these genes. This might suggest that similar to miRNA, tsRNA could also play a role in RNA activation or promoter targeting.

RNAi factors have been previously detected in the nucleus (41). A recent report also found intronic sequences using Ago PAR-CLIP in adult stem cells and show that Ago can lead to post-transcriptional gene silencing in miRNA dependent manner in nucleus (42). It remains enigmatic why miRNA loaded Ago would not promote NRS. Perhaps various modifications on tsRNA could lead to different target RNA:tsRNA stability. Another independent report identified tsRNA-intronic target sequence hybrids in Ago1 crosslinking, ligation and sequencing of hybrids (CLASH-seq). These data support the notion that introns are targeted by RNAi in the nucleus.

The key question which immediately arises from these observations is how nascent RNA can be targeted if splicing occurs co-transcriptionally. A recent report, using direct nanopore sequencing of nascent RNA, showed that in human cells, splicing and transcription are not physically coupled: most introns are not spliced until RNA pol II transcribes 4 kilobases (kb) downstream of the intron (22). This might provide a window of opportunity for introns to serve as a regulatory platform for RNA dynamics. Furthermore, retention of introns is a well-studied phenomenon of alternative splicing (23). Pre-mRNAs with retained introns can potentially serve as an optional source for nuclear RNAi targeting.

Overall, our data revealed a nuclear gene silencing mechanism that is mediated by Dicer generated tsRNA. Ago2 loaded with tsRNA finds RNA targets through miRNA-like base pairing. Ago2 then slices the nascent RNAs and consequently prevents their translation into proteins (Figure 7D). This mechanism is distinct from PTGS and TGS as it takes place in the nucleus and does not affect the transcriptional state of the gene. As tRNAs are evolutionarily conserved across many species, it is likely that NRS is a prevalent mechanism of gene expression regulation in eukaryotes. Furthermore, our data showing that synthetic tsRNA can silence multiple proto-oncogenic genes in cancer cells suggest that NRS could be an attractive novel mechanism for cancer therapy.

## DATA AVAILABILITY

All data are available in the supplement; raw data are deposited in GEO accession number GSE126751. Combined RNA pol II ChIP-seq and chrRNA-seq data can be viewed here: https://genome-euro.ucsc.edu/s/rita1510/HEK_tRNA. Custom written codes are available in Github: https://github.com/rita1510/tRNA_codes.git.

## Supplementary Material

gkac022_Supplemental_Files
