# Supplementary material for: Dicer dependent tRNA derived small RNAs promote nascent RNA silencing
Source: Nucleic Acids Res. 2022 Jan 20;50(3):1734–52. doi: 10.1093/nar/gkac022 (PMC8860591; doi:10.1093/nar/gkac022)
Supplement: gkac022_Supplemental_Files [file gkac022_supplemental_files.zip › Suppl Figure and Legends.pdf]

Supplementary Figure 1

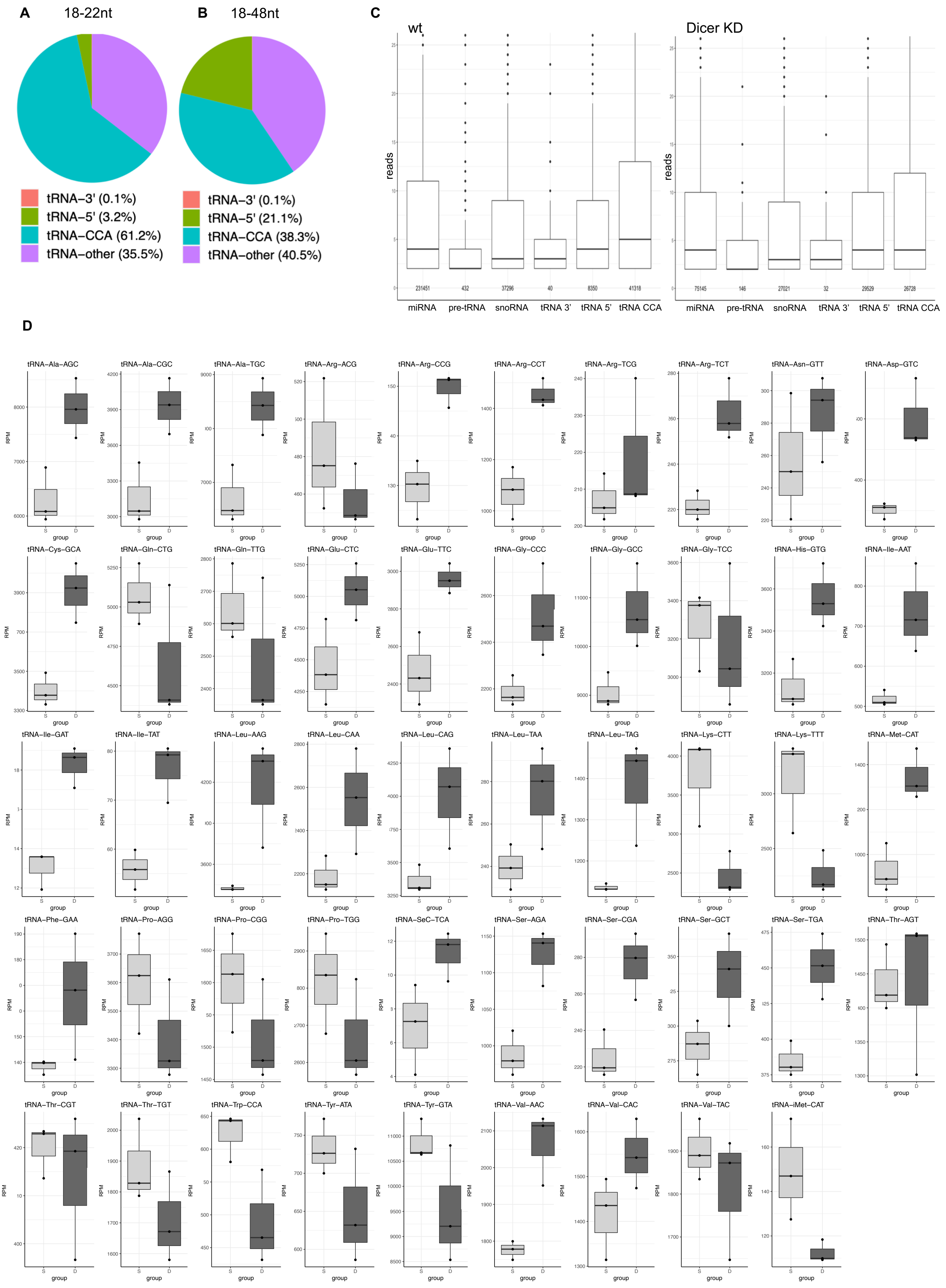

Supplementary Figure 2

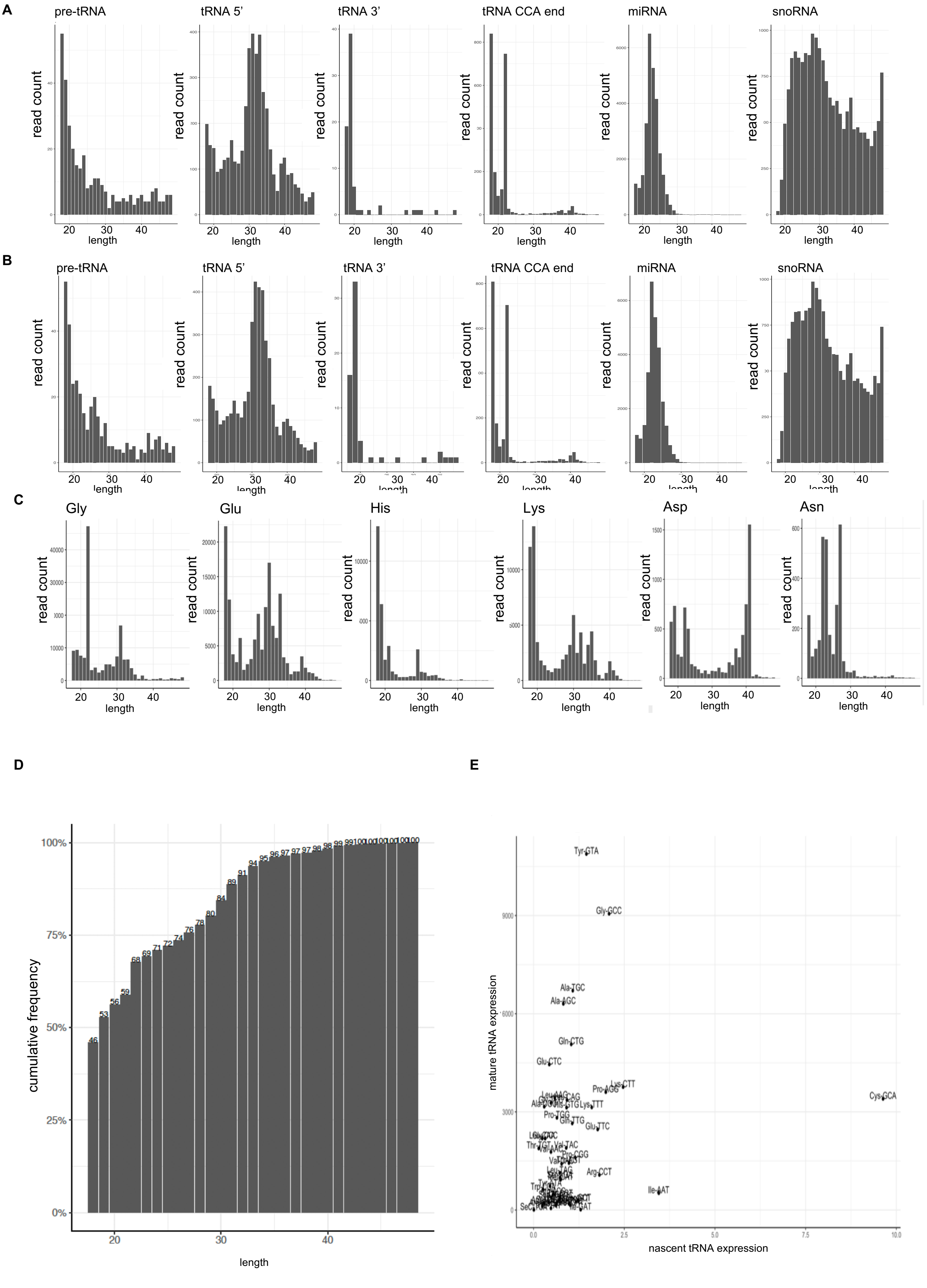

Supplementary Figure 3

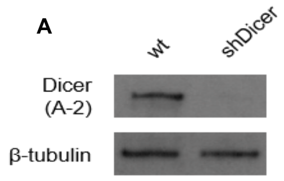

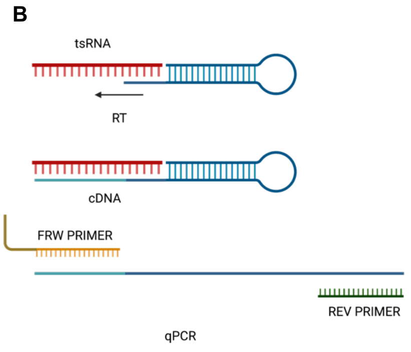

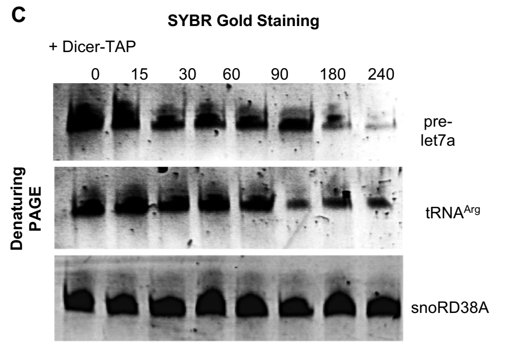

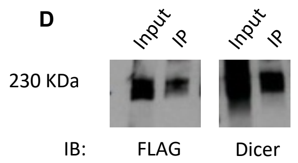

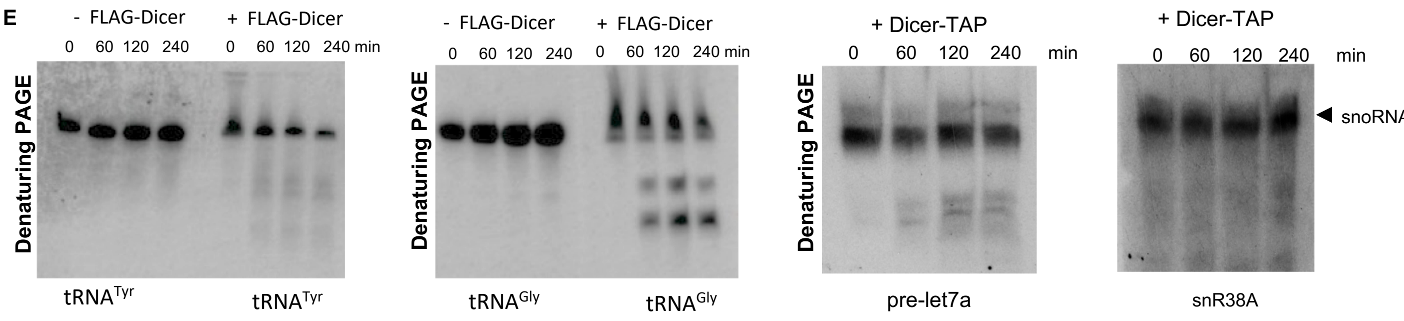

F

AGO	grouped_seqs_in_thisAGO_tRNAmapped/total_tsRNA_groups	#sequnces aligned to tRNA	ungrouped_seqs_in_thisAGO_tRNAmapped/total_tsRNA	total_dataset_seqs	#sequences aligned to miRNA
AGO1	192/192	101077	413/415	2260482	120304
AGO2	161/192	12550	300/415	3606988	242740
AGO3	192/192	227614	415/415	3803842	395432
AGO4	179/192	81954	383/415	3677793	266112

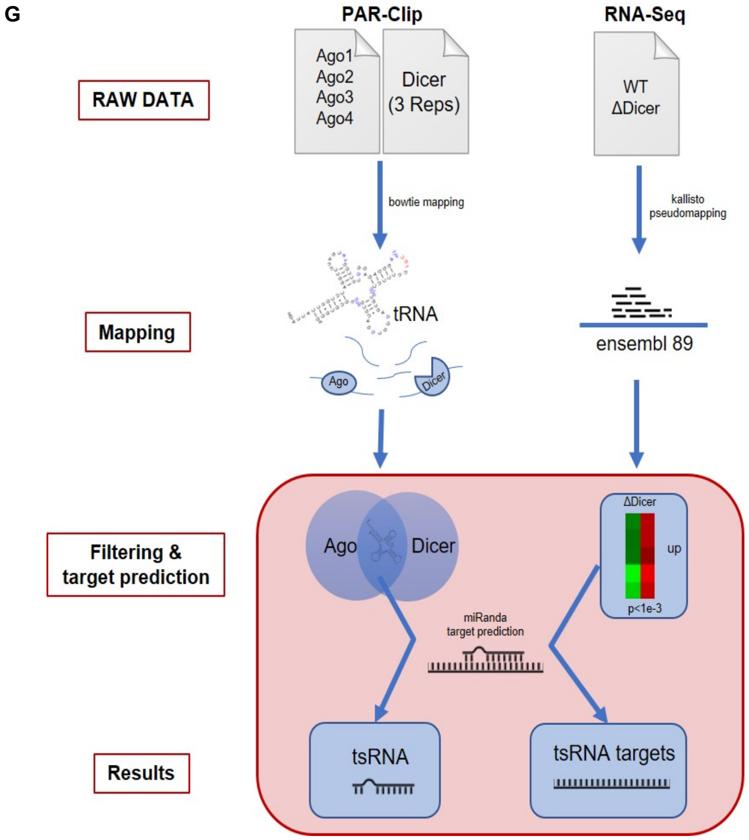

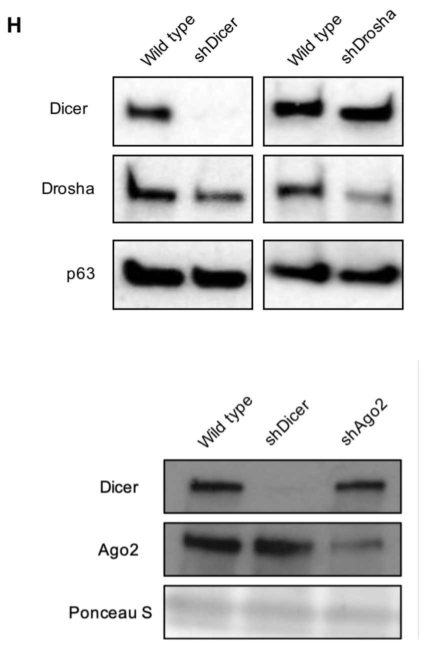

Supplementary Figure 4

A

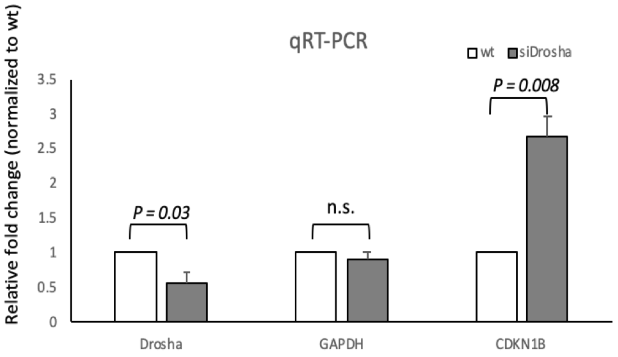

steady state RNA

B

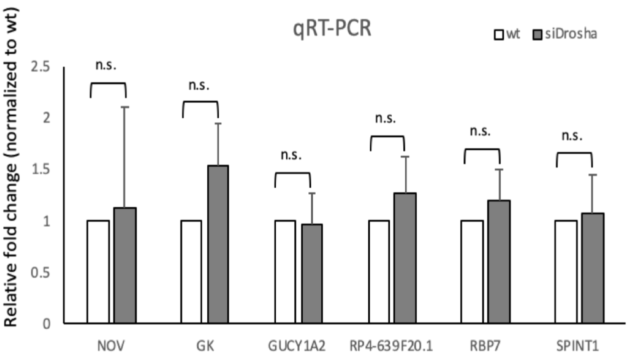

nascent RNA

C

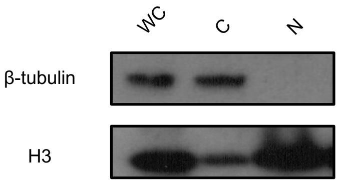

Supplementary Figure 5

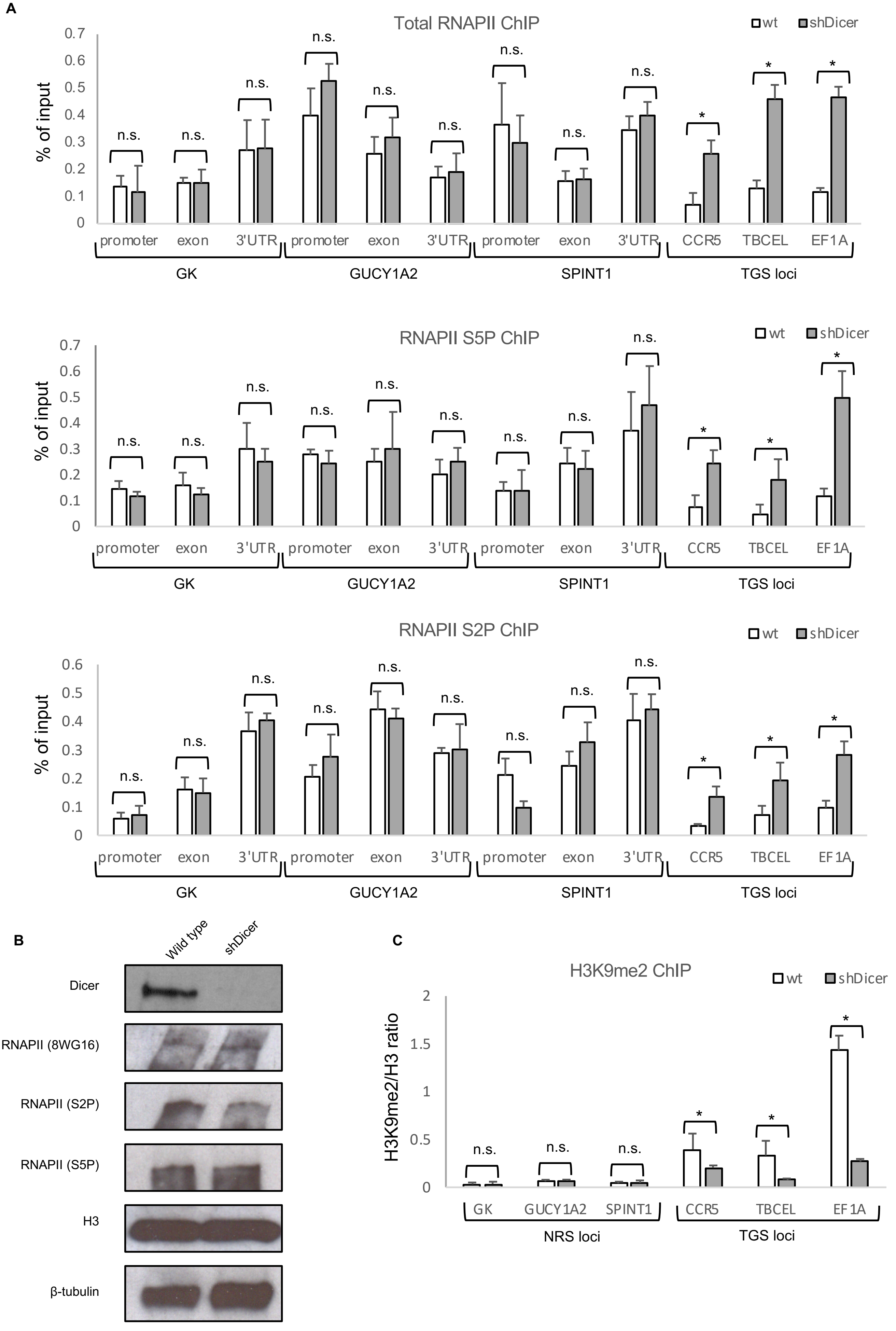

Supplementary Figure 6

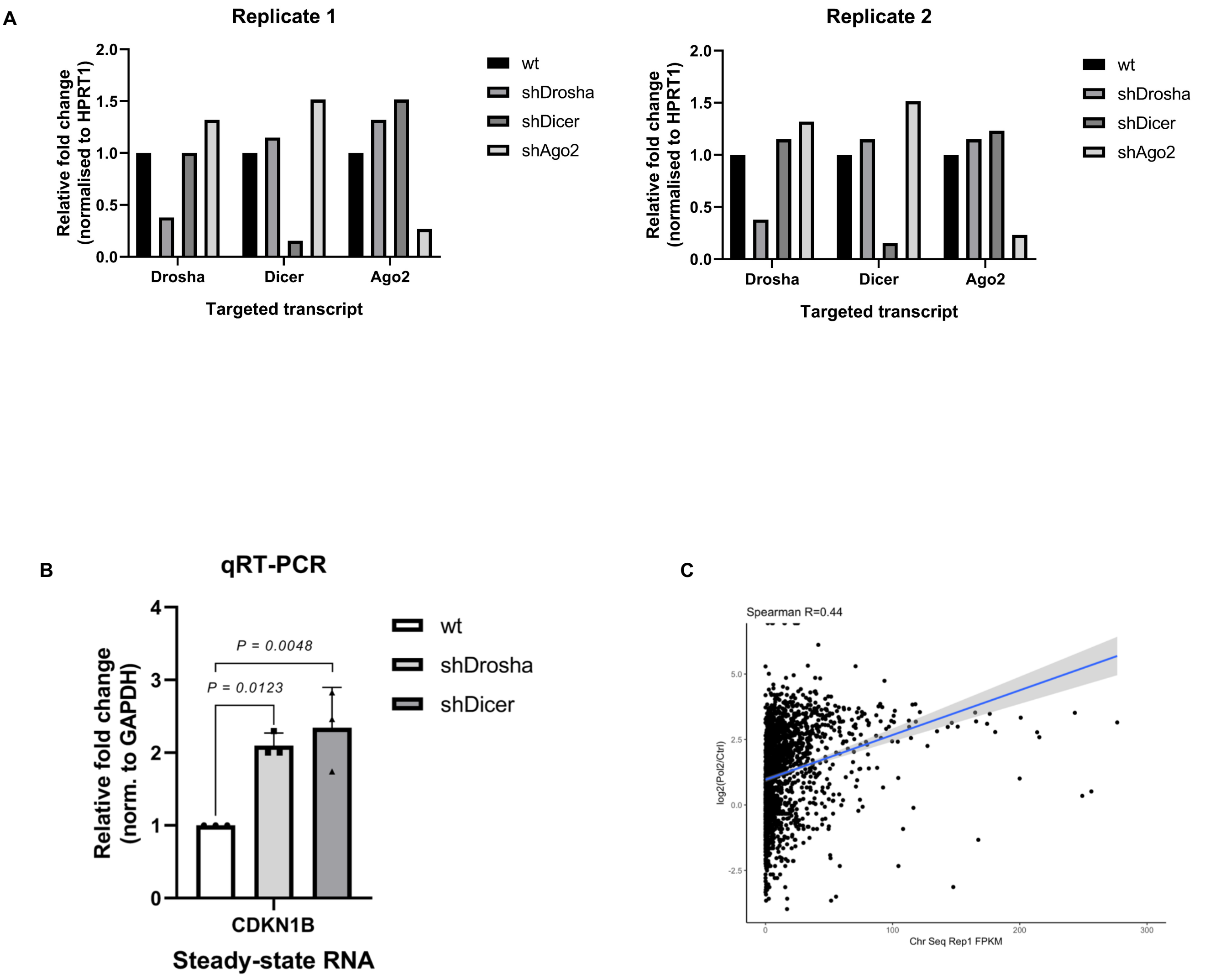

Supplementary Figure 7

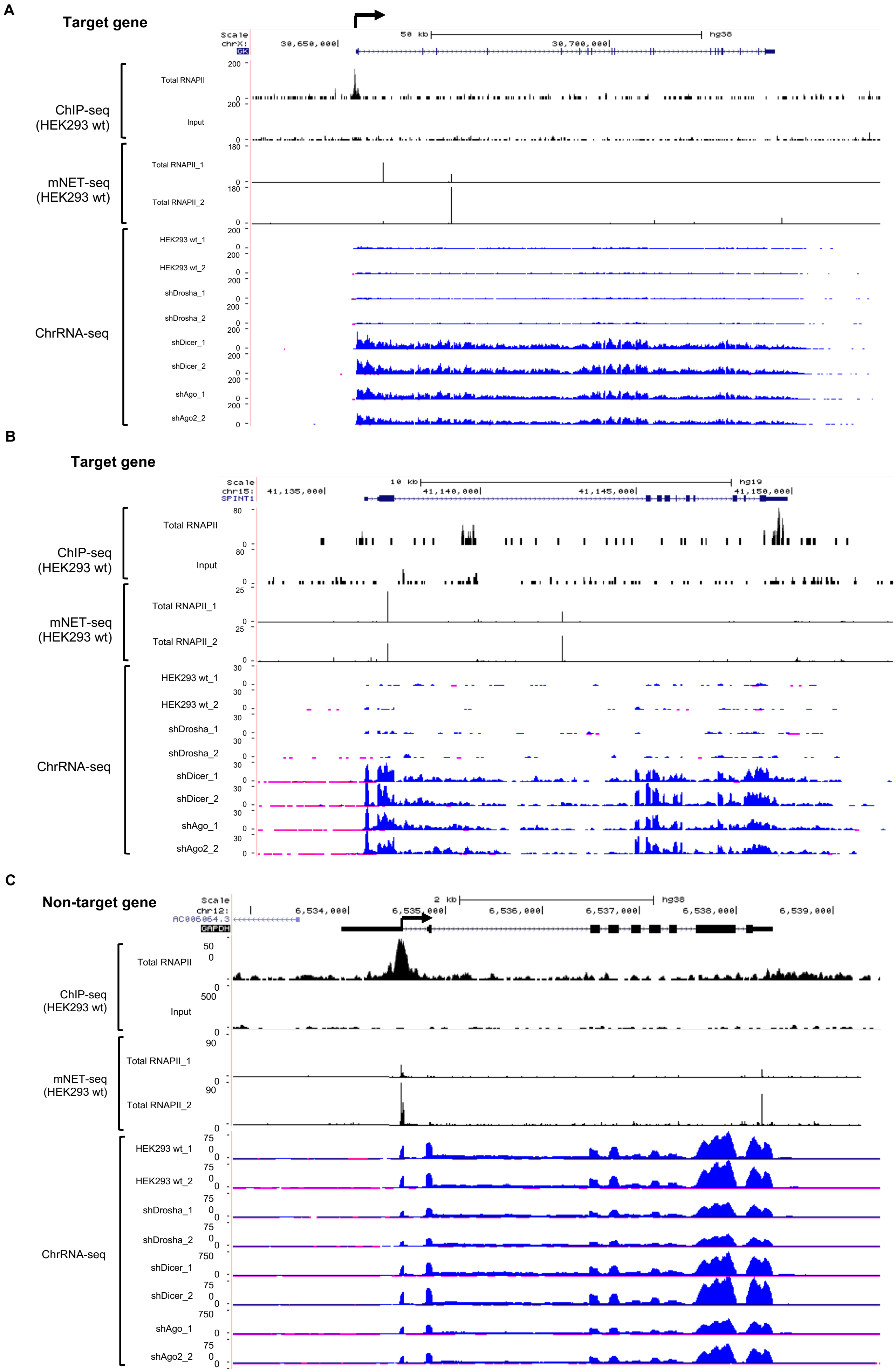

Supplementary Figure 8

A

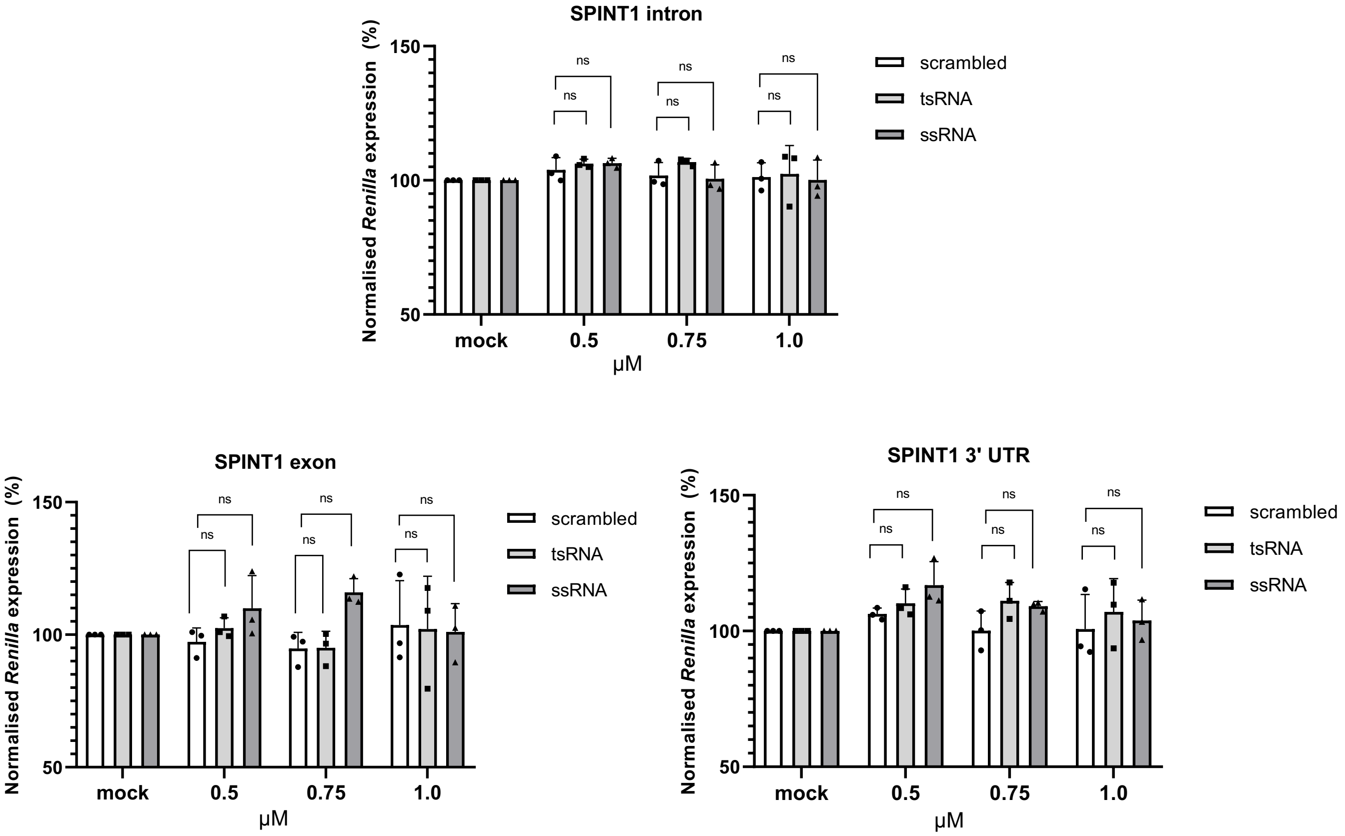

B

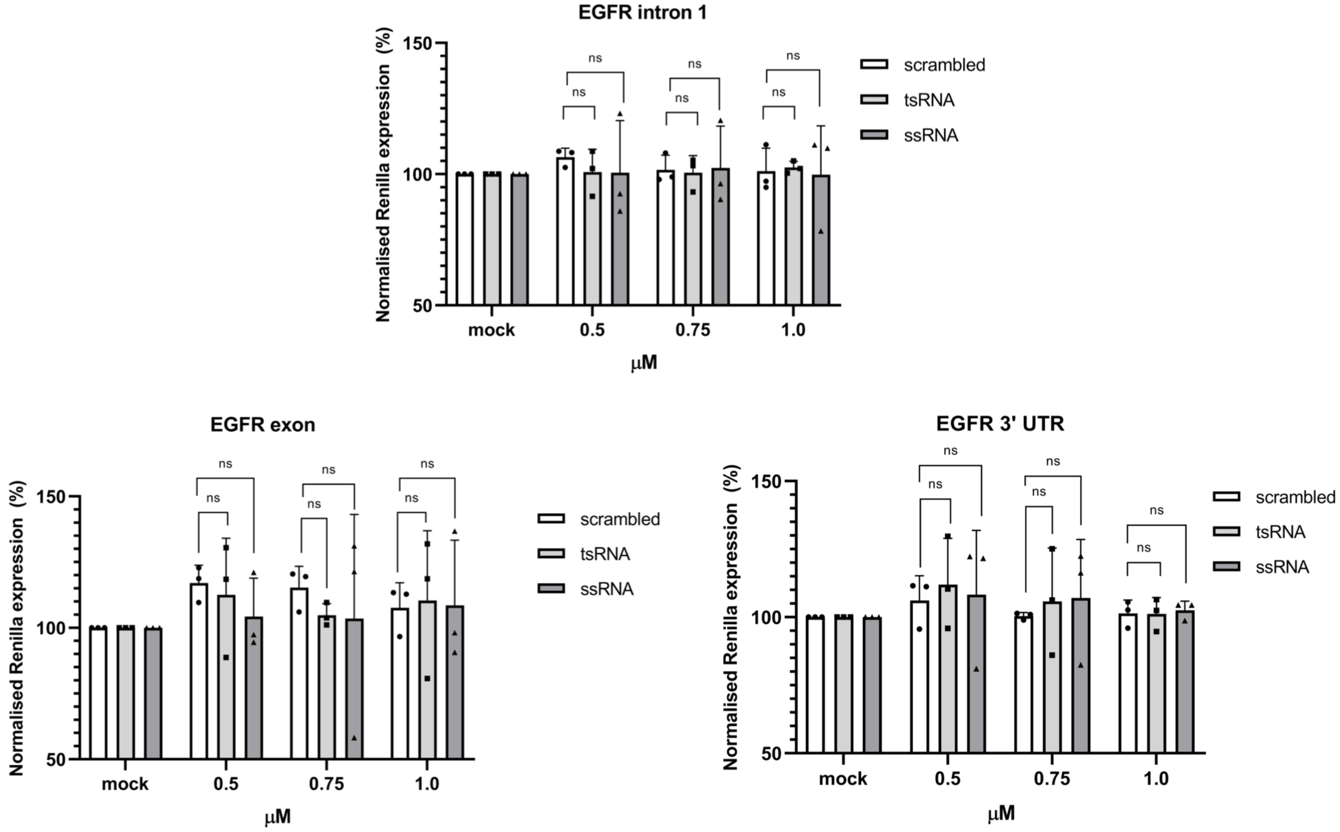

Supplementary Figure 9

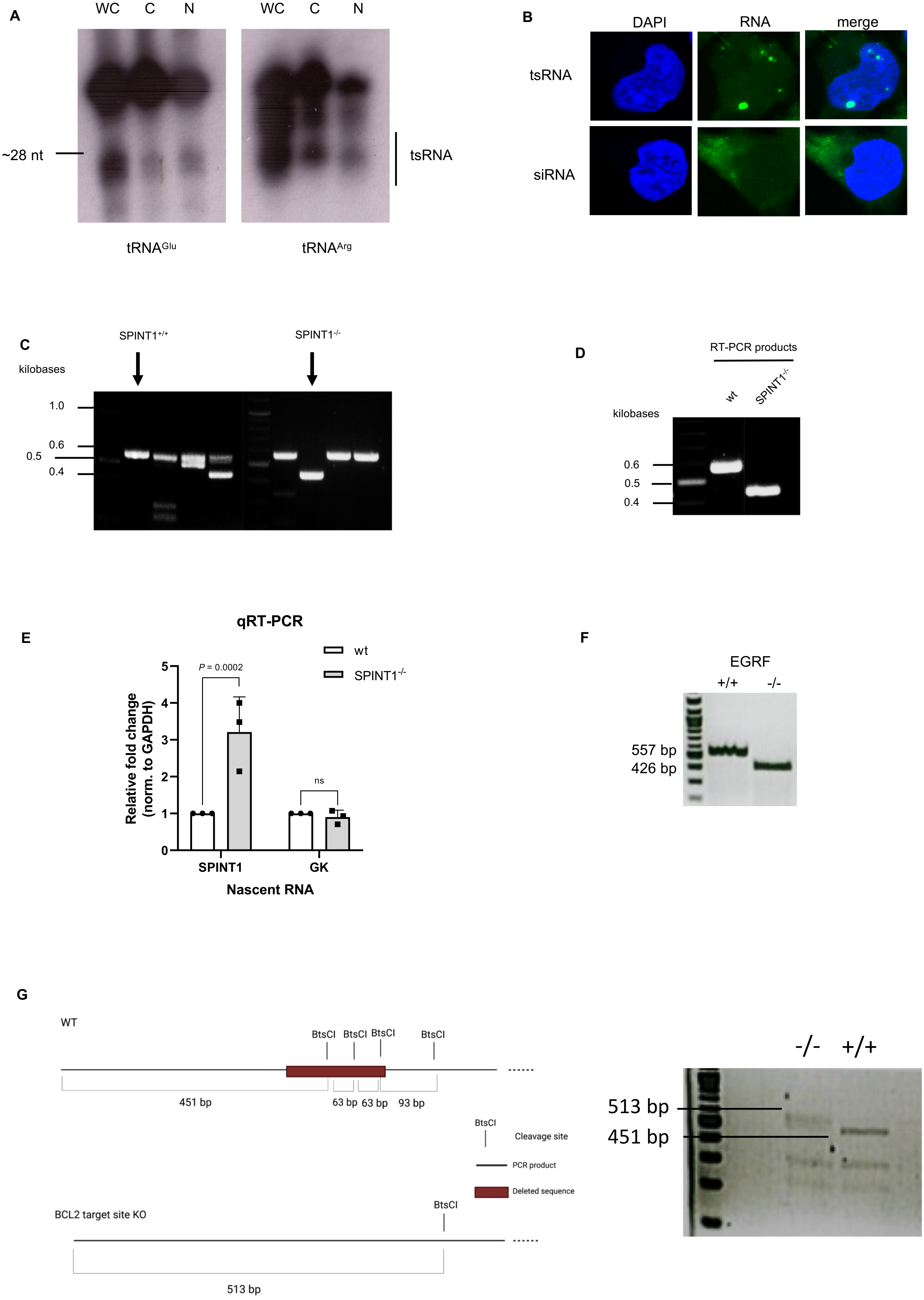

Supplementary Figure 10

A

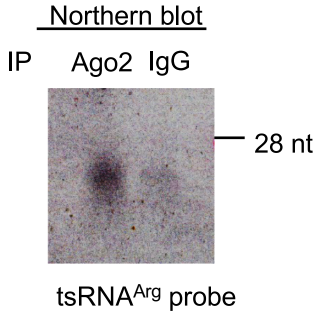

B

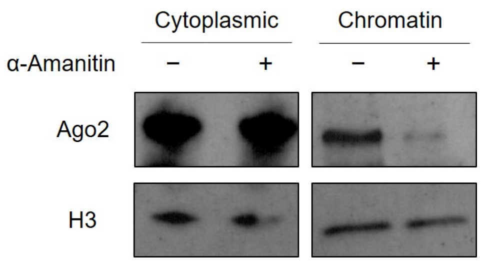

C

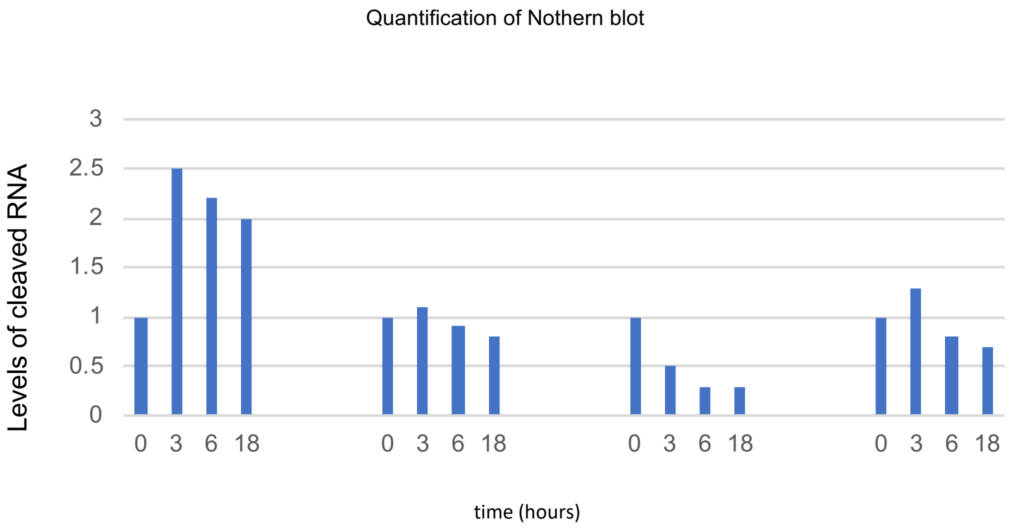

D

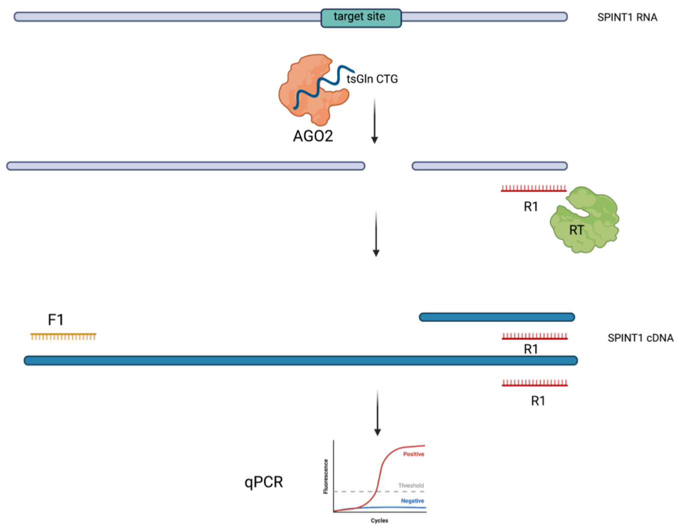

Supplementary Figure 11

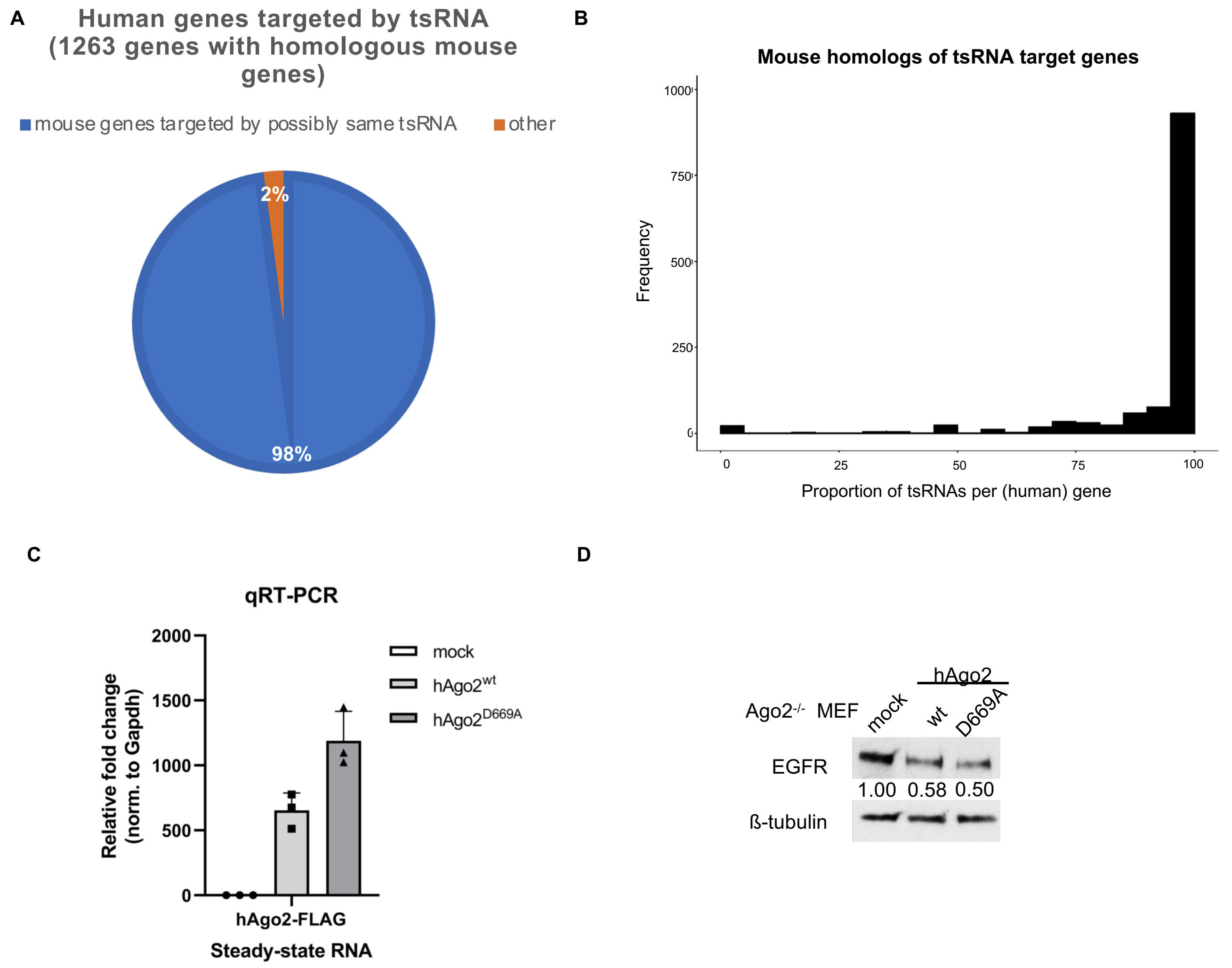

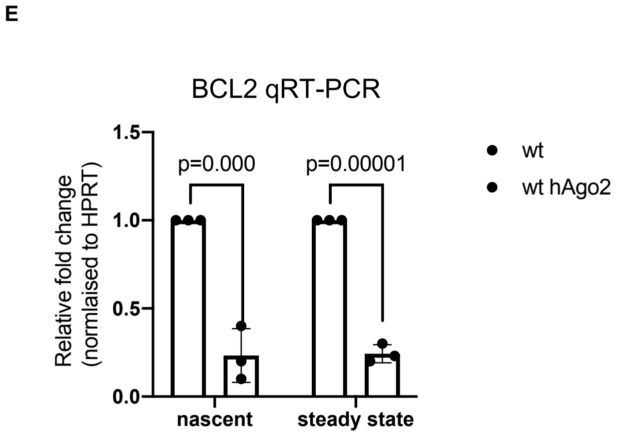

Supplementary Figure 12

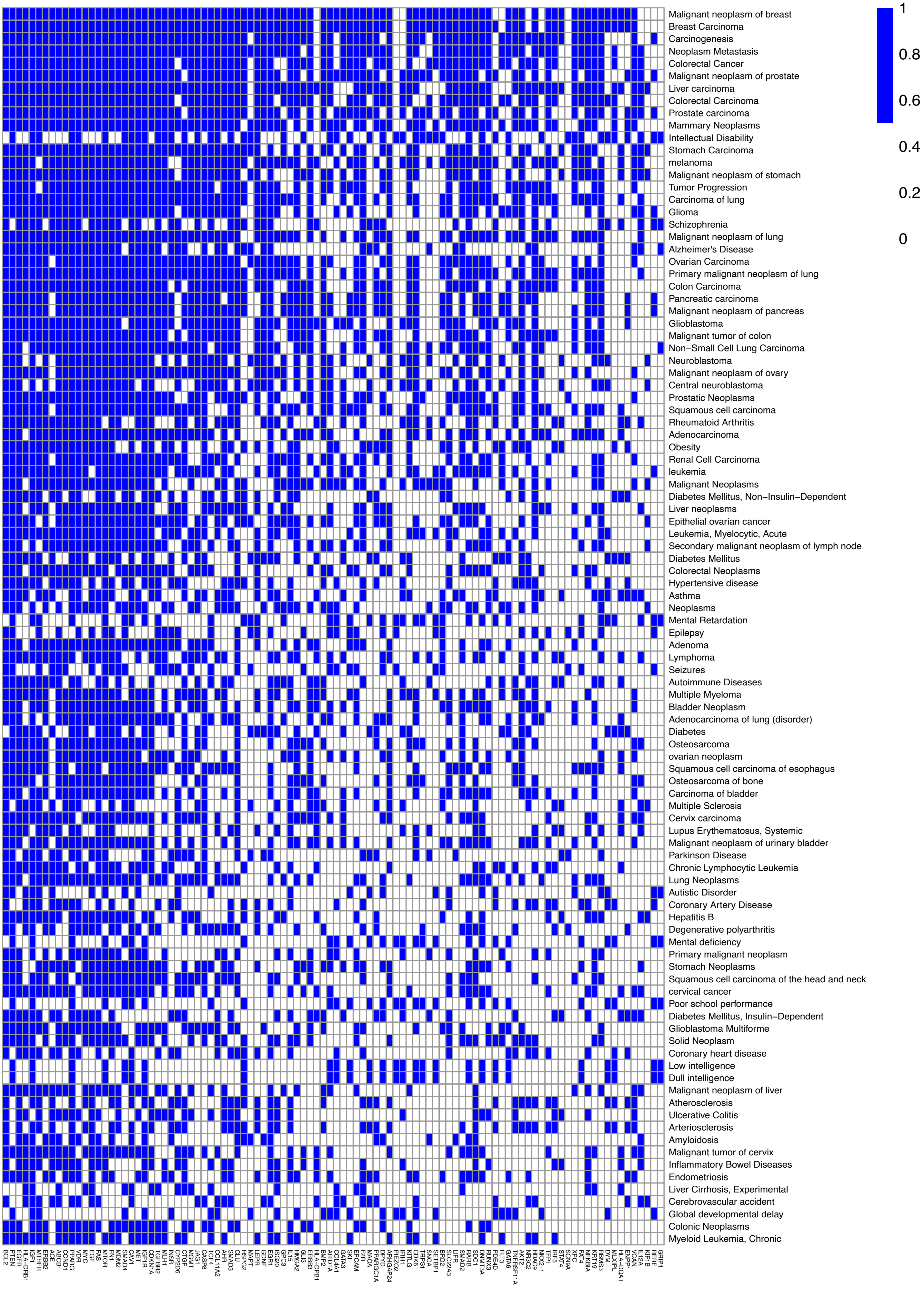

Supplementary Figure 13

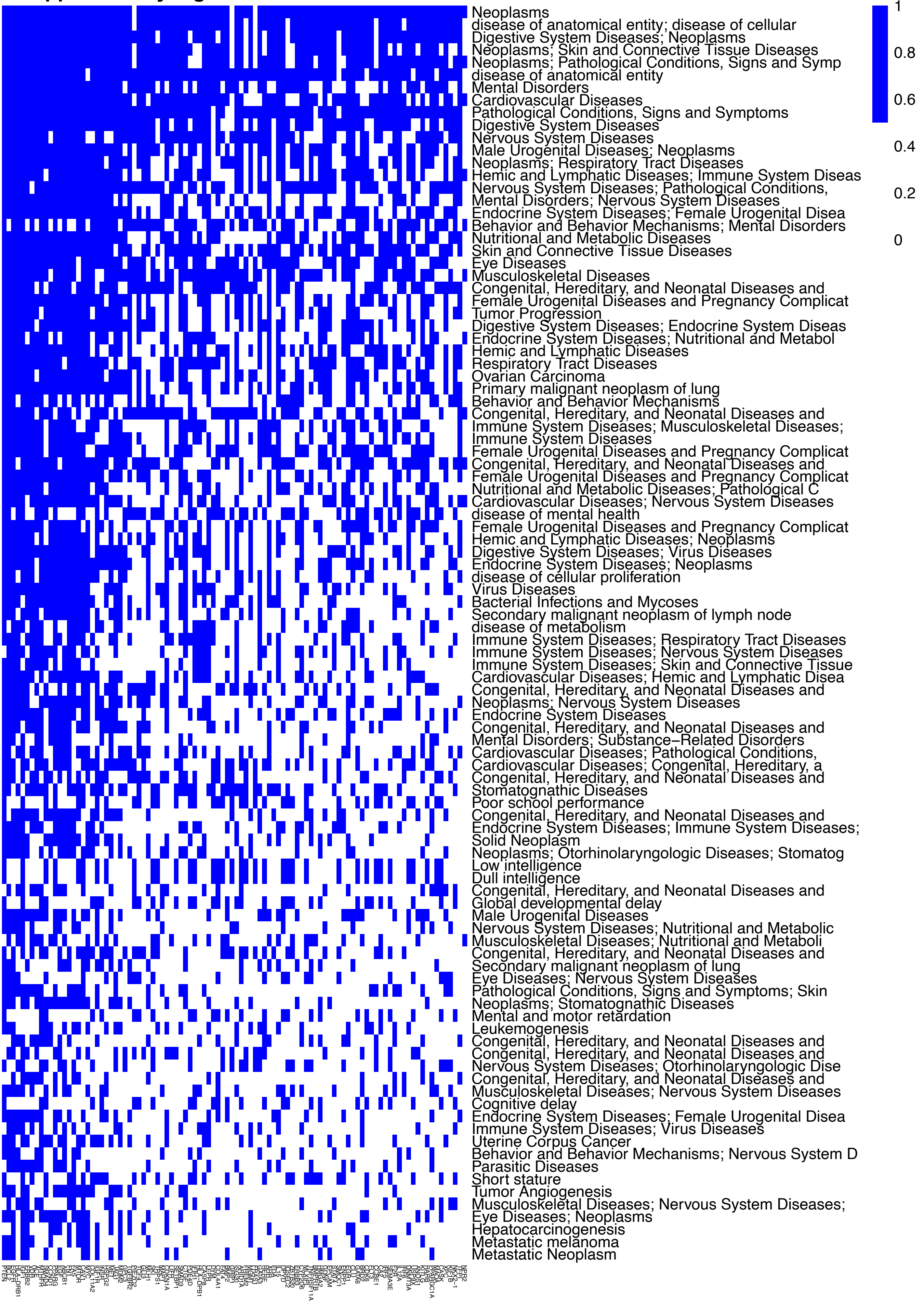

Supplementary Figure 14

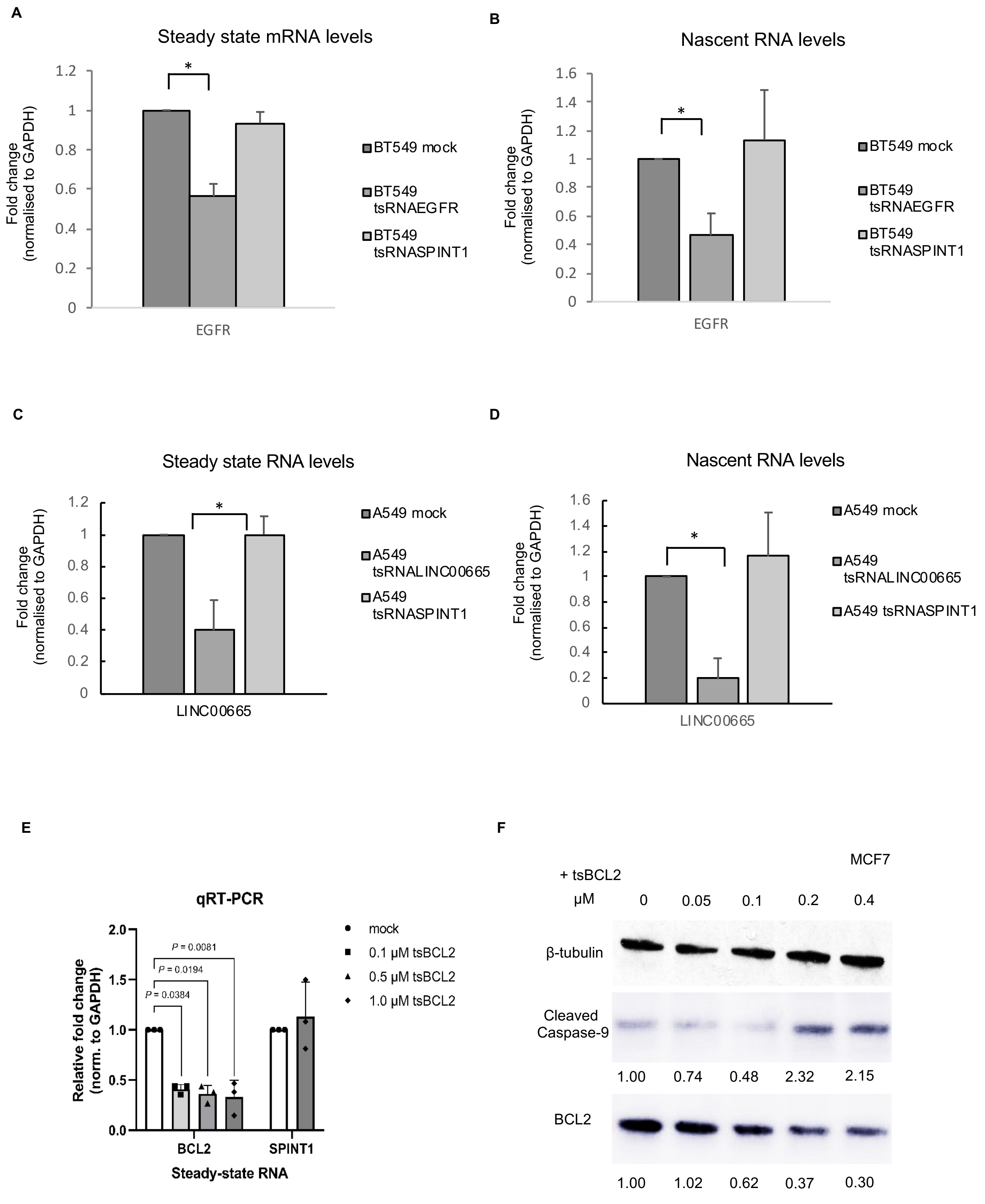

Dicer dependent tRNA derived small RNAs promote nascent RNA silencing

Arianna Di Fazio[1, †], Margarita Schlackow[1, †], Sheng Kai Pong[1, †], Adele Alagia[1] and Monika Gullerova[1, *]

Supplementary Figure Legends

Supplementary Figure 1: Analyses of sRNA-seq data A, Pie chart showing distribution for 18-22nt long tsRNA origins. **B,** As in A for tsRNA of lengths 18-48nt. **C,** Box plots showing abundance of tsRNA derived from various origins isolated from wt and Dicer KD samples. Numbers below the box plots correspond to total levels of sRNA sequences for each type. **D,** Box plots showing tsRNA derived from various tRNA isotypes isolated from wt and Dicer KD samples.

Supplementary Figure 2: Length analysis of tsRNAs A, Bar charts representing the length distribution of various tsRNA types based on sequence length, in nucleotides (nt) for 2nd replicate. **B**, As in A for 3rd replicate. **C.** Bar charts representing the length distribution of various tsRNA types based on sequence length, in nucleotides (nt) for selected tRNA isotypes. **D,** cumulative length distribution plot illustrating total number of sequences fall at depicted length. **E,** Plot showing the abundance of tRNA and tsRNAs. tRNA abundance was determined from ChrSeq data and the R summarizeOverlaps function and normalized to mapped reads. tsRNA read abundances for all 3 WT were extracted from the SPORTS1.1 "summary.txt"-files, and used the read count for the "mature tRNA"-mapping subclasses.

Supplementary Figure 3: Analyses combining Dicer and Ago PAR-CLIP with sRNA-seq A, Western blot showing levels of Dicer upon induction of Dicer KD. **B,** Diagram showing Stem loop qRT-PCR approach for quantification of tsRNA **C**, Image of a SYBR-Gold-stained polyacrylamide gel showing signals for let7a precursor (pre-let7a), tRNA^{Arg} and snoRD38A at different time points after the addition of TAP-tagged Dicer. **D**, Western blot showing successful FLAG-Dicer pulldown by Anti-FLAG magnetic beads **E**, Northern blot images showing signals for tRNA^{Tyr} , tRNA^{Gly} , let7a (positive control) and snR38A (negative control) precursors and their corresponding sRNA at different time points after the addition of TAP-

tagged or FLAG-Dicer. **F,** Table summarising tRNA derived sequences identified in Ago PAR-CLIP data sets. **G,** Diagram representing the bioinformatic workflow of tsRNA target prediction using RNA-seq data. **H,** Western blot images showing successful knockdown of Dicer, Drosha and Ago2.

Supplementary Figure 4: Target genes are upregulated in nucleus and cytoplasm upon Dicer knockdown. A, Bar chart showing steady state RNA levels of Drosha, GAPDH and CDKN1B in wt and Drosha KD samples. **B,** Bar chart showing the log2 fold change of the nascent RNA levels of six selected target genes upon Drosha knockdown. **C,** Western blot image showing successful preparation of subcellular fractions.

Supplementary Figure 5: Levels of total and active RNA pol II interacting with target genes remain unchanged upon Dicer knockdown. A, Bar charts showing signals of total and active forms of RNA pol II (in percentage of input) across the promoter, exon and 3' UTR sites of three selected target genes upon Dicer knockdown. Additional TGS loci, as indicated, were used as controls. Data were derived from n=3 biological repeats and 3 technical repeats. **B,** Western blot images showing signals of total and active forms (S2P and S5P) of RNA pol II in wild type and Dicer knockdown cells, with ß-tubulin and histone H3 as loading controls. **C,** Bar chart showing levels of H3K9me2/H3 at selected loci including NRS and TGS promoters. Data were derived from n=3 biological repeats and 3 technical repeats.

Supplementary Figure 6: tsRNA target prediction using chrRNA-seq data. A, Bar charts showing the fold change of steady-state Drosha, Dicer and Ago2 transcripts, measured by qRT-PCR, upon Drosha, Dicer and Ago2 knockdown (replicates 1 and 2) from samples used for ChromRNA-seq. **B,** Bar chart showing steady state RNA levels of CDKN1B in wt, Drosha and Dicer knockdown samples. **C,** Graph plotting log2 fold change of RNA pol II signal relative to control against normalised reads of genes measured by chrRNA-seq.

Supplementary Figure 7: tsRNA target genes genomic profiles. A, Combined snapshot of RNA pol II ChIP-seq, mNET-seq and chrRNA-seq profiles across target gene GK (*n = 2*). Normalised read counts are indicated. **B**, As in A for SPINT1 **C**, Combined snapshot of RNA pol II ChIP-seq,

mNET-seq and chrRNA-seq profiles across non-target gene GAPDH (*n = 2*). Normalised read counts are indicated.

Supplementary Figure 8: tsRNAs target genes in introns. A, Bar chart showing the knockdown efficiency of SPINT1 intron (other than intron with target site), exon and 3'UTR regions using the tsRNA, fully complementary (ssRNA) and scrambled sequences is plotted as a percentage (SD; $*P = 0.019$) of the normalized *Renilla* luciferase expression. The luciferase activity of the mock transfected cells was set as 100%. All tested 5'-phosphorylated ssRNA were transfected at 1µM, 0.75 µM and 0.5 µM. Silencing activities were measured at 24 h post-transfection. **B,** As in A for EGFR gene.

Supplementary Figure 9: tsRNA is detected in nucleus and cytoplasm, and targets introns for gene silencing. A, Northern blot images showing signals of $tRNA^{Glu}$ and $tRNA^{Arg}$ and their corresponding tsRNAs from whole-cell, cytoplasmic and nuclear fractions of wild type 293T cells. **B.** Representative confocal images showing the localisation of fluorescently labelled tsRNA and siRNA (in green) after transfection into BT549 cells. DAPI is in blue. **C,** Screening for SPNT1 target site mutants followed by CRISRP-Cas9 gene editing and single cell sorting. Image of DNA gel electrophoresis showing signals for PCR products from colonies derived from single cells. 579bp-size band corresponds to wild type allele, while 444bp-size band corresponds to mutant allele. **D**, Image of DNA gel showing signals for RT-PCR products from wild type, heterozygous ($SPINT1^{+/-}$) and homozygous ($SPINT1^{-/-}$) cells. **E,** Bar chart showing the fold change of expression of nascent *SPINT1* and *GK* transcripts normalised to GAPDH, measured by qRT-PCR, in wild type, heterozygous $SPINT1^{+/-}$ and homozygous $SPINT1^{-/-}$ cells. **F,** Screening for EGFR target site mutants followed by CRISRP-Cas9 gene editing and single cell sorting. Image of DNA gel electrophoresis showing signals for PCR products from colonies from single cells. 557bp-size band corresponds to wild type allele, while 426bp-size band corresponds to mutant allele. **G,** Screening for BCL2 target site mutants followed by CRISRP-Cas9 gene editing and single cell sorting. Diagram summarises the selection strategy. Mutant colonies will resulted in longer PCR product after BstCI restriction. PCR gel showing results from restriction reaction.

Supplementary Figure 10: Ago2 is the effector of NRS A, Image of northern blot showing signals of tsRNA^{Arg} from RNA immunoprecipitated with Ago2, with IgG-immunoprecipitated RNA as negative control. **B**, Western blot showing signals of Ago2 from cytoplasmic and chromatin protein fractions. Treatment of α-Amanitin is indicated above. **C,** Bar chart showing quantification of Northern blot shown in Figure 6A. **D**, Diagram showing experimental strategy for Ago2 cleavage assay, as shown in Figure 6 B and C.

Supplementary Figure 11: Evolutionary conservation of NRS A, Pie chart showing the proportion of human tsRNA target genes which have mouse homologues targeted by possibly same tsRNA. **B**, Histogram showing the proportion of tsRNAs from each tsRNA group targeting a human gene, which may also target the corresponding mouse homologues. **C,** Bar chart showing expression of wt and mutant hAgo2 in MEFs using qRT-PCR. **D,** Western blot image showing signals for EGFR upon mock transfection and transfection of expressing wt hAgo2 and hAgo2^{D669A} in MEFs. **E,** Bar chart showing relative fold change of mouse nascent and steady state *BCL2* transcripts, measured by qRT-PCR, upon mock transfection and transfection of plasmids expressing wild type human Ago2 (wt hAgo2) in MEFs.

Supplementary Figure 12: Association between tsRNA target genes and various diseases. Heatmap, sorted bi-directionally for gene-disease associations, of top 100 disease against top 100 target genes. Blue indicates a match while white indicates no match.

Supplementary Figure 13: Association between tsRNA target genes and various disease classes. Heatmap, sorted bi-directionally for gene-disease associations, of top 100 disease classes against top 100 target genes. Blue indicates a match while white indicates no match.

Supplementary Figure 14: Transfection of synthetic, single-stranded tsRNA results in repression of proto-oncogenic genes. A, Bar chart showing the fold change of steady-state levels of *EGFR* upon transfection of tsRNA against *EGFR* in BT549 cells, with transfection of tsRNA against *SPINT1* as negative control. **B**, Bar chart showing the fold change of nascent levels of *EGFR* upon transfection of tsRNA against *EGFR* in BT549 cells, with transfection of tsRNA against *SPINT1* as negative control. **C**, Bar chart showing the fold change of steady-state levels of *LINC00665* upon transfection of tsRNA against *LINC00665* in A549 cells, with

transfection of tsRNA against *SPINT1* as negative control. **D**, Bar chart showing the fold change of nascent levels of *LINC00665* upon transfection of tsRNA against *LINC00665* in A549 cells, with transfection of tsRNA against *SPINT1* as negative control. **E**, Bar chart showing the fold change of steady-state levels of *BCL2* upon transfection of tsRNA against *BCL2* in MCF7 cells. SPINT1 was used as negative control. **F**, Western blot images showing signals of cleaved Caspase-9 and BCL2 upon transfection of tsRNA against *BCL2* at increasing concentrations in MCF7 cells, with ß-tubulin as loading control.
