# Supplementary material for: Dicer dependent tRNA derived small RNAs promote nascent RNA silencing
Source: Nucleic Acids Res. 2022 Jan 20;50(3):1734–52. doi: 10.1093/nar/gkac022 (PMC8860591; doi:10.1093/nar/gkac022)
Supplement: gkac022_Supplemental_Files [file gkac022_supplemental_files.zip › Supplementary files 1-3 description.docx]

Supplementary files 1-3 or NAR-00394-A-2021.R2

Supplementary file 1: this file contains all profiles for all replicates of sRNA in pdf file for sRNAs mapping to precursor tRNAs and mature tRNAs, as well as allowing no mismatches in mapping/allowing up to 3 mismatches in mapping

Supplementary file 2: this file contains differential expression analysis of ChromRNA-seq data from shDicer, shAgo2 and shDrosha samples.

Supplementary file 3: this file contains predicted target table for all upregulated gene in shDicer vs wt samples, all down-regulated genes in shDicer vs wt samples. It also shows, how many gene targeted by NRS are also targeted by miRNA. For each target we provide information on tsRNA sequences, their positions within the targets and also conservation score for each target.
