# Supplementary figures and images for: Dicer dependent tRNA derived small RNAs promote nascent RNA silencing
Source: Nucleic Acids Res. 2022 Jan 20;50(3):1734–52. doi: 10.1093/nar/gkac022 (PMC8860591; doi:10.1093/nar/gkac022)

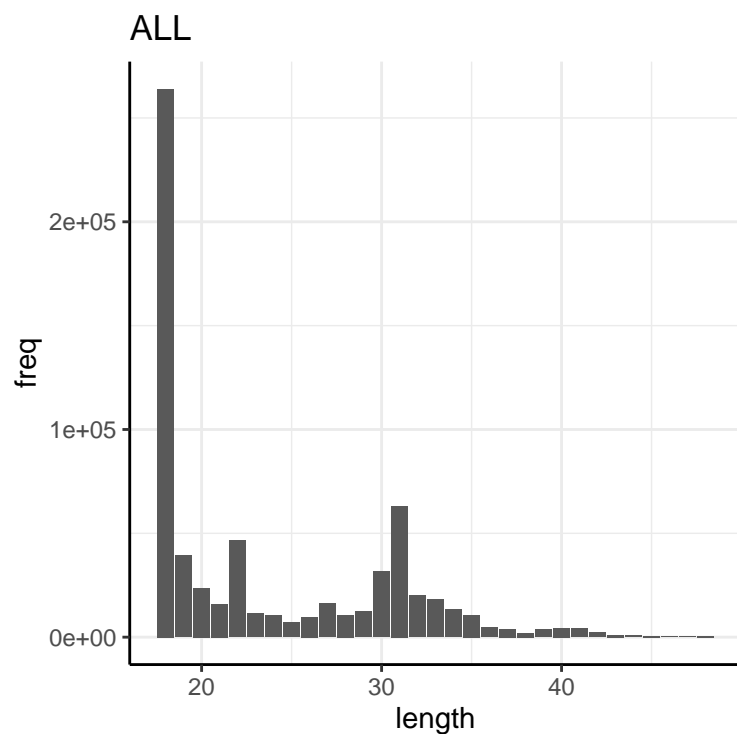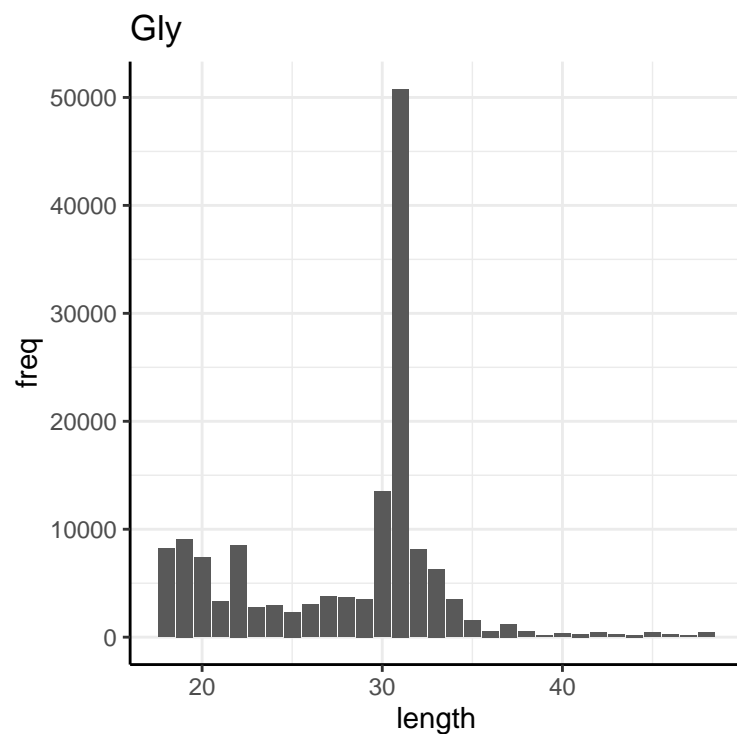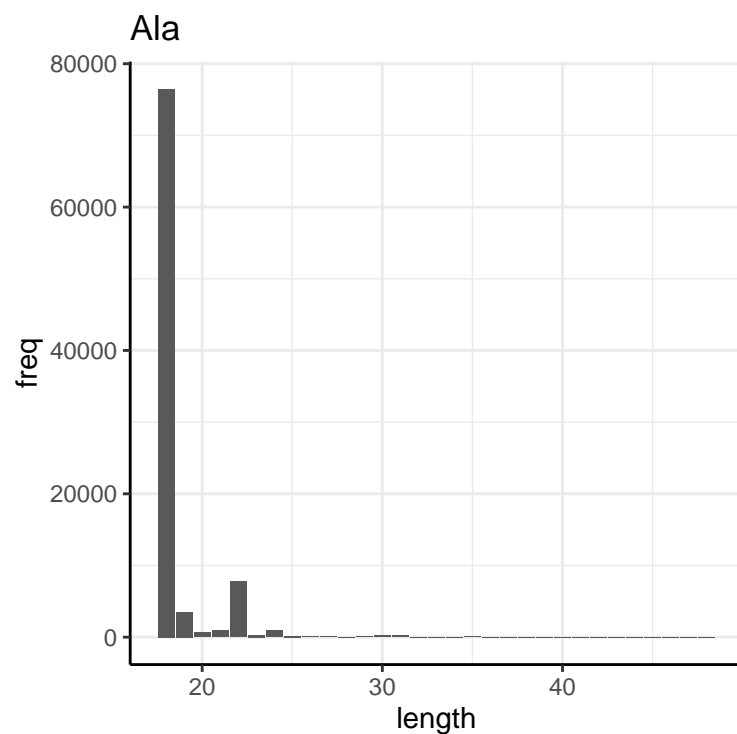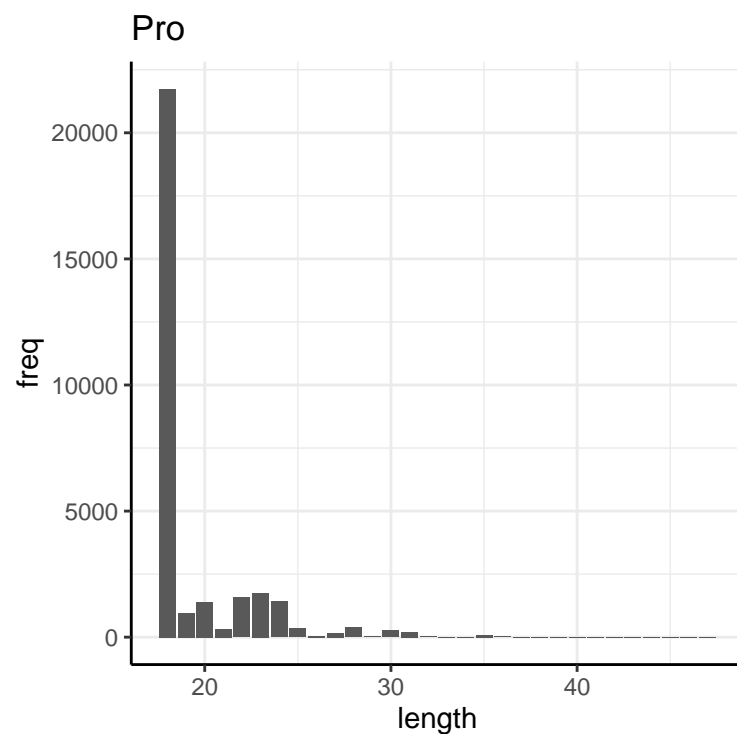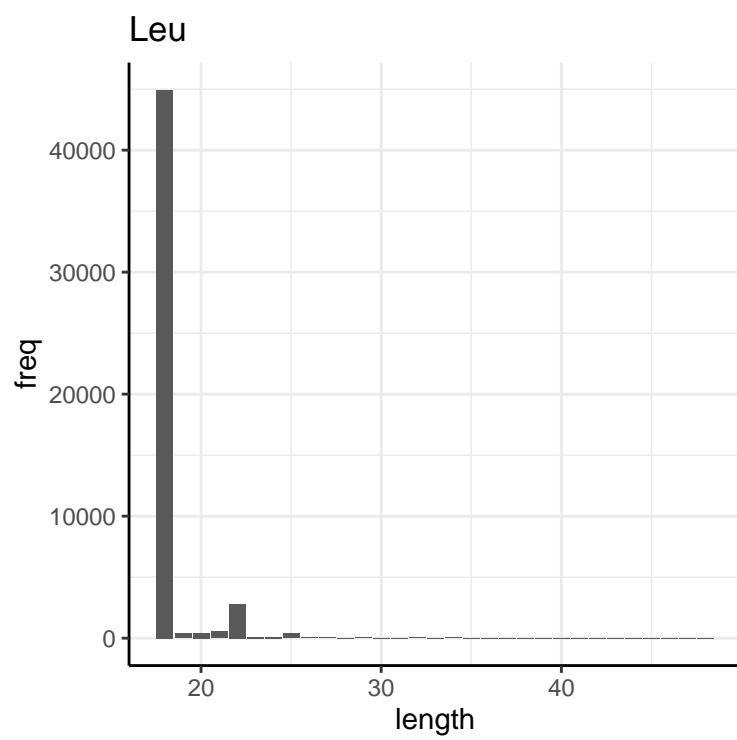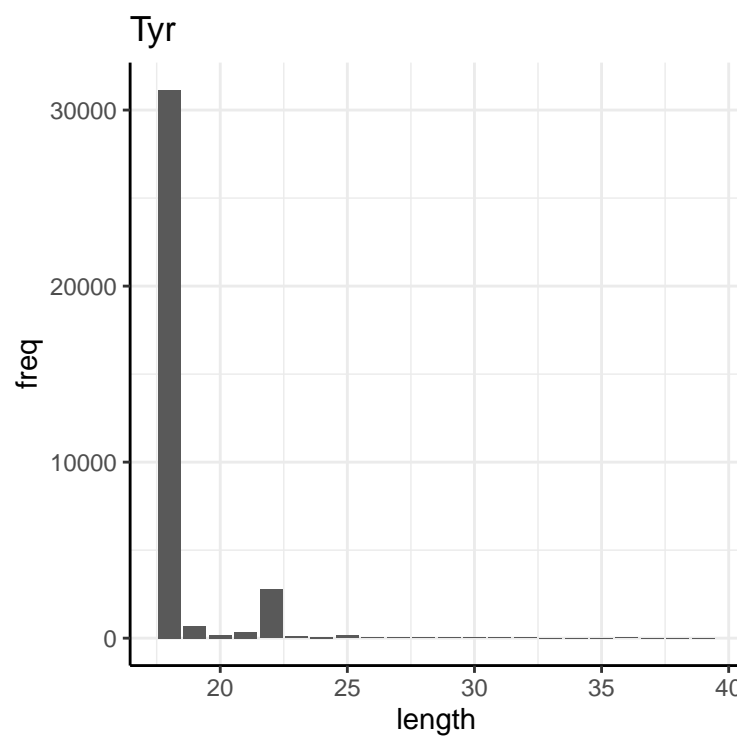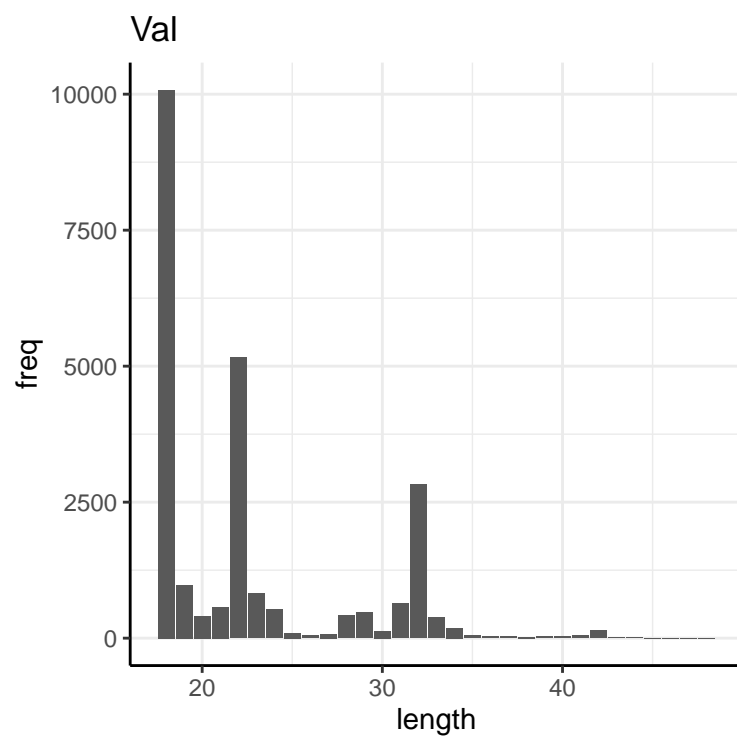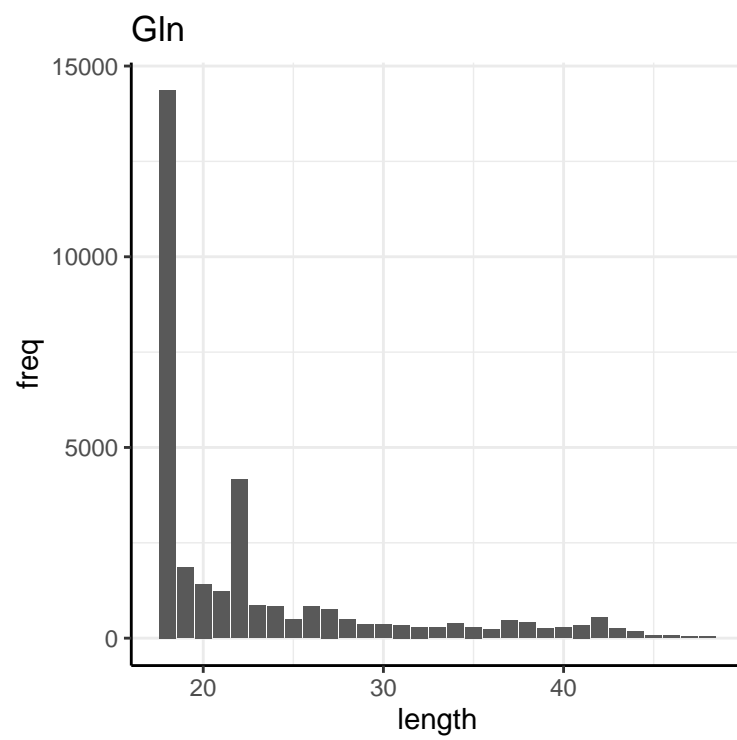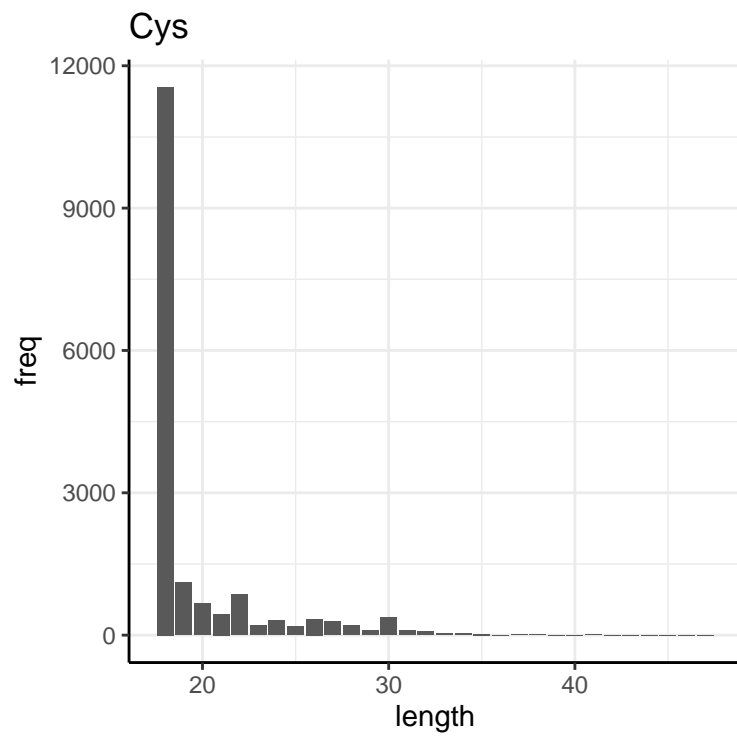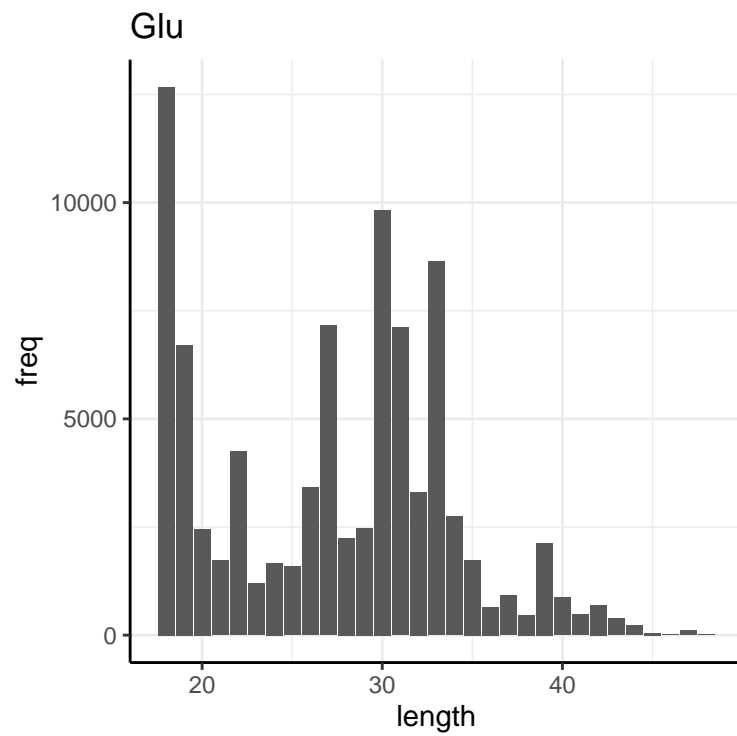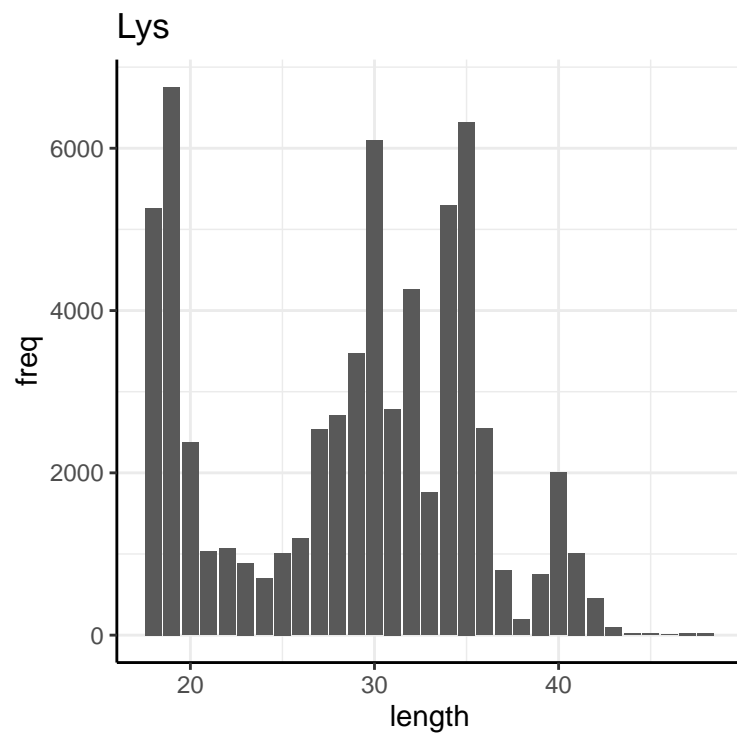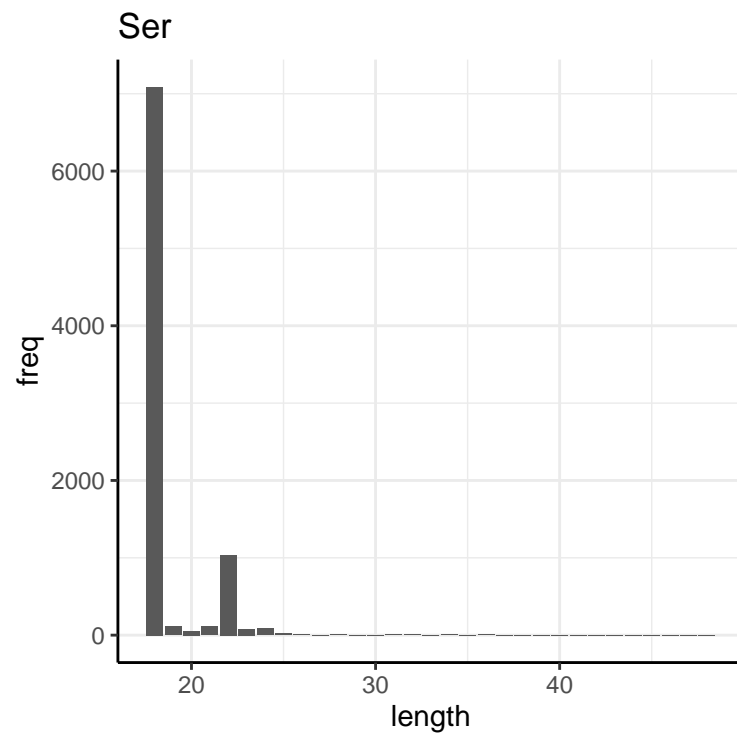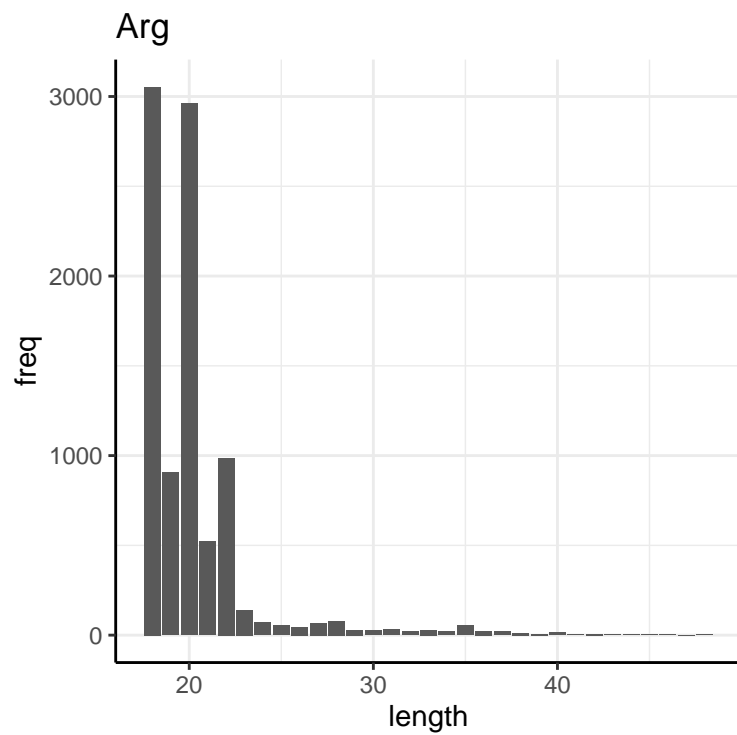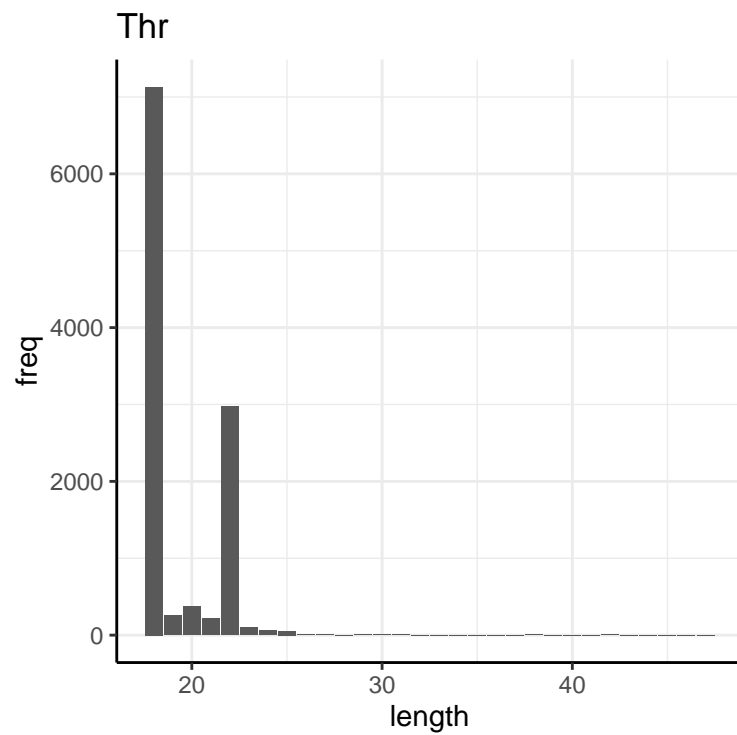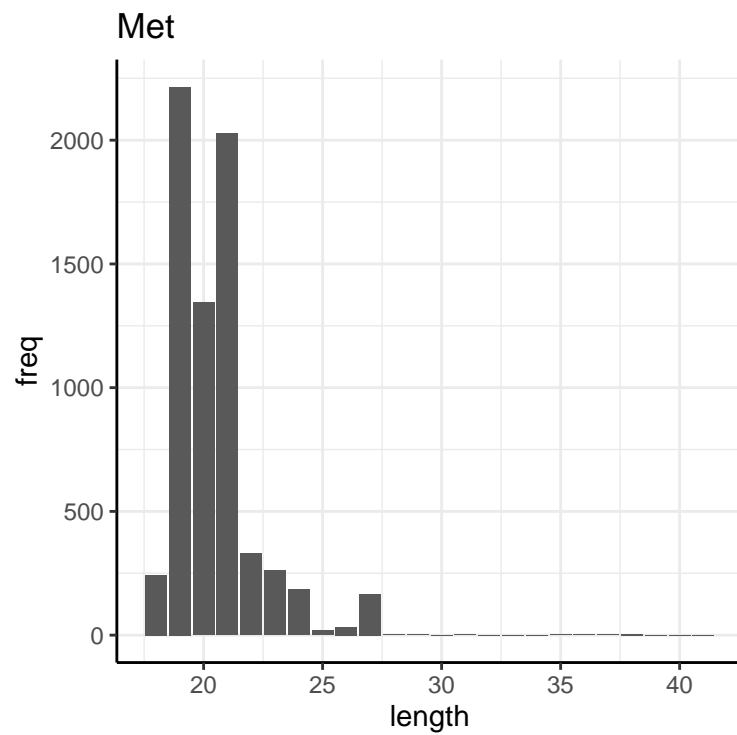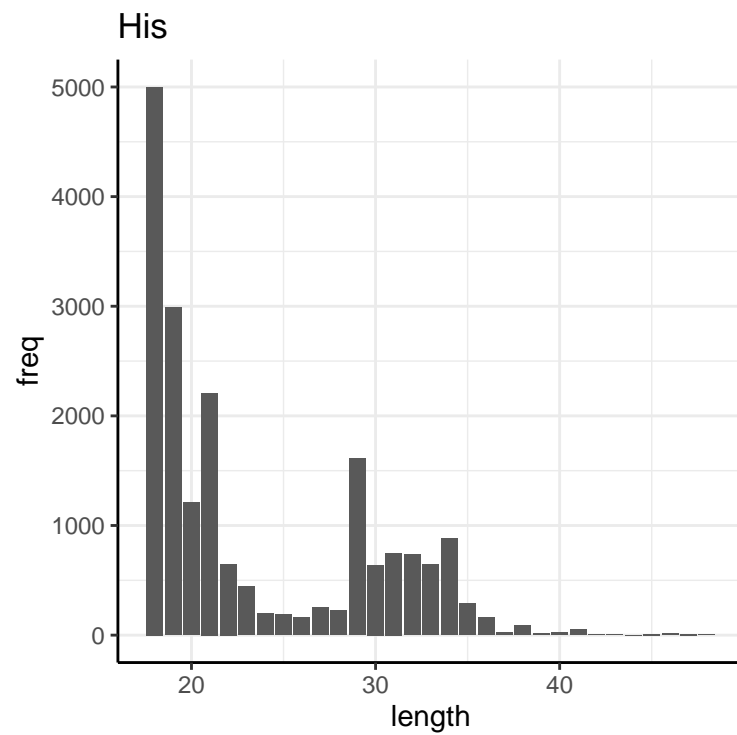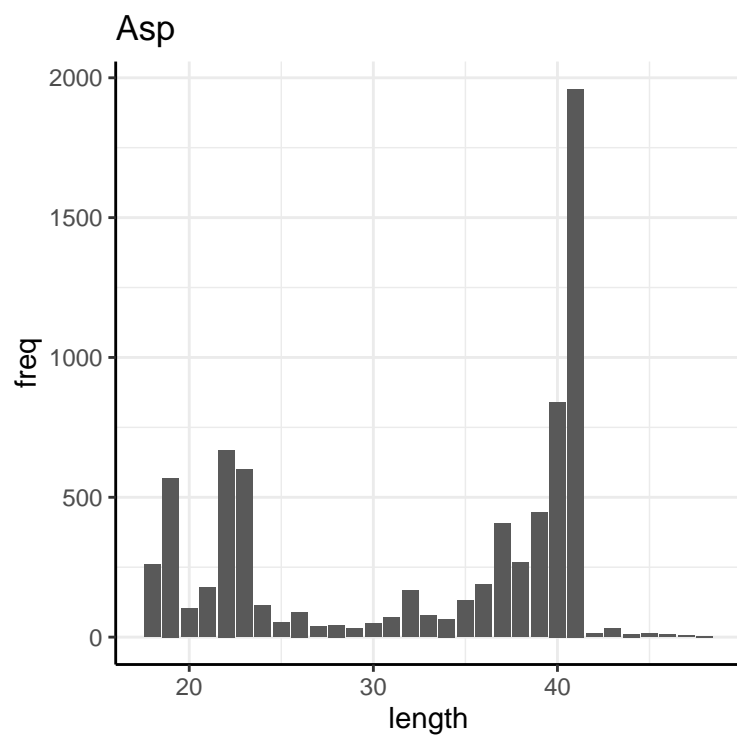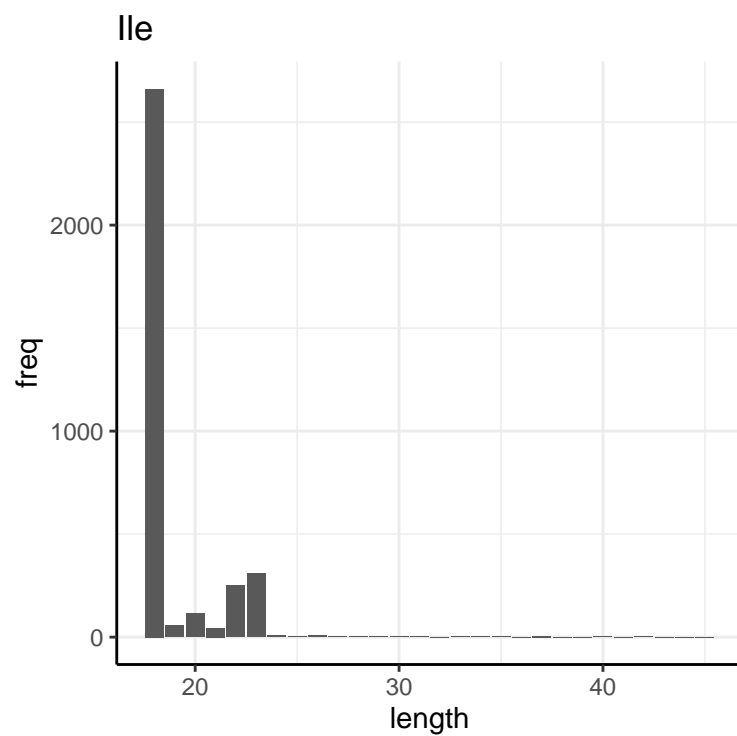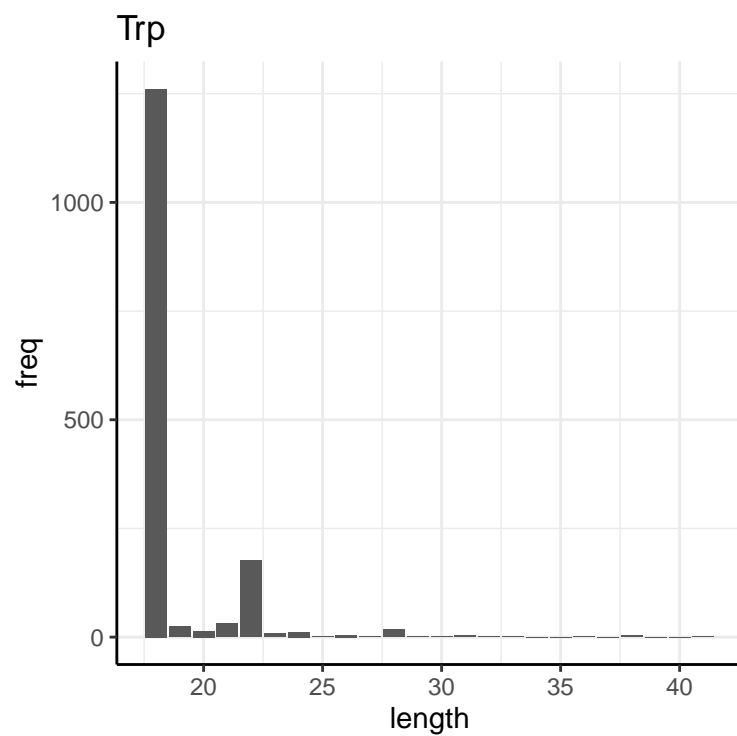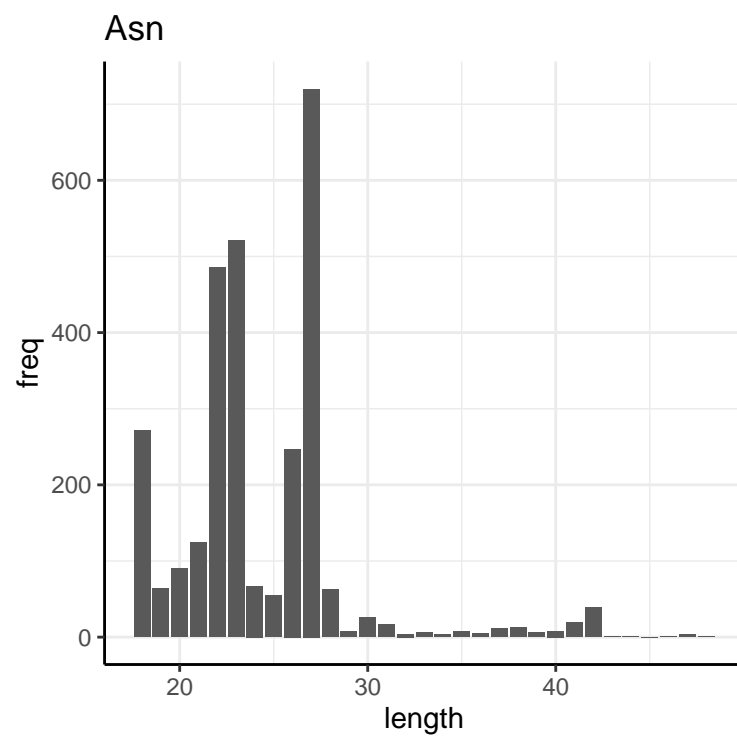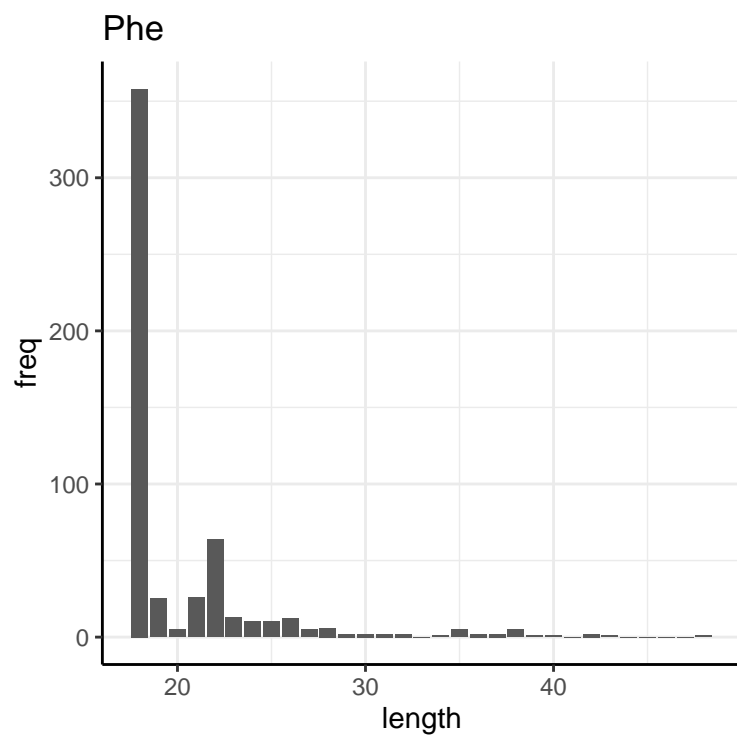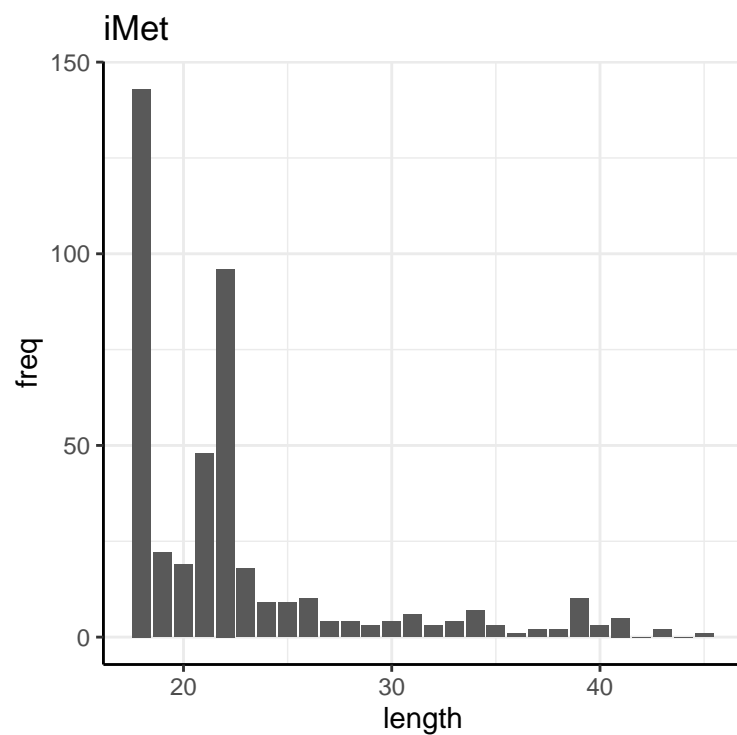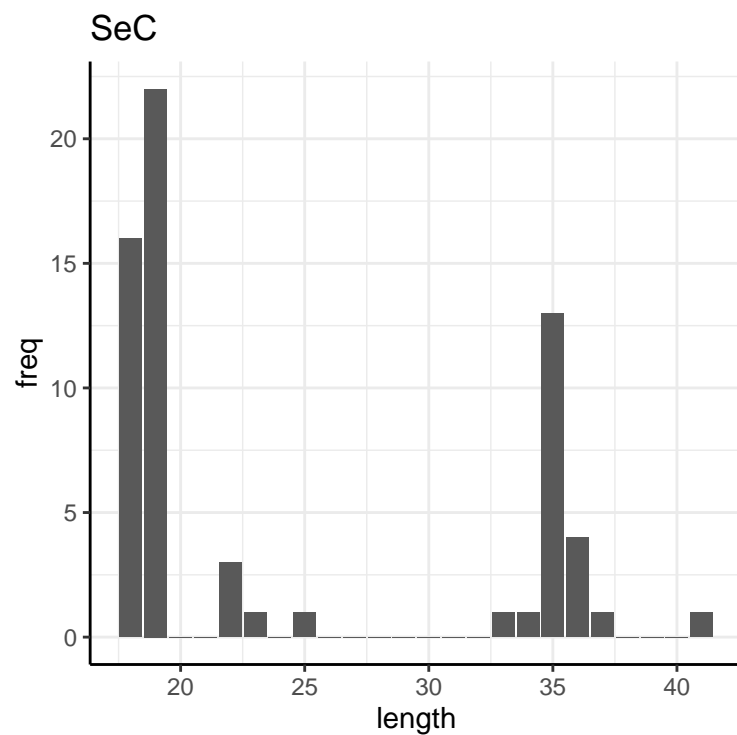

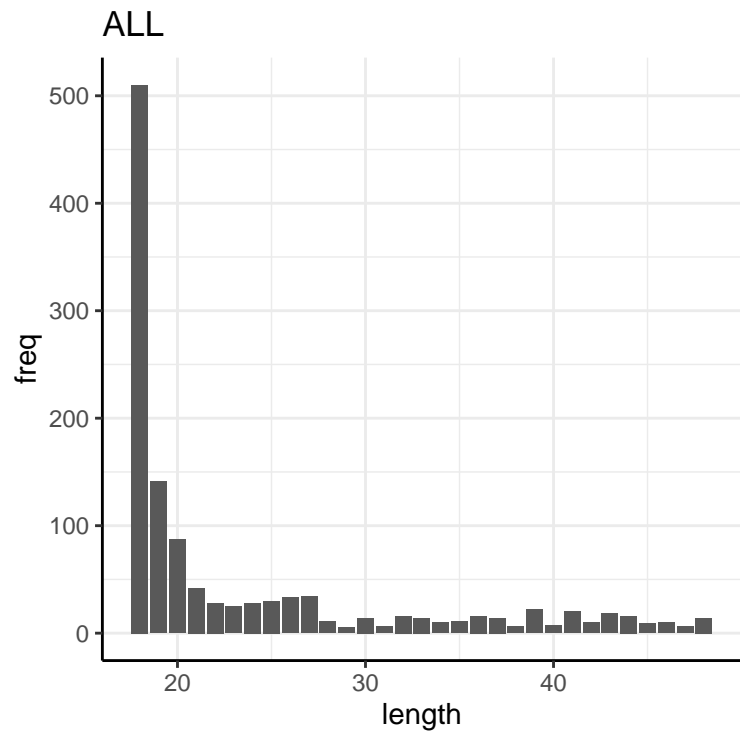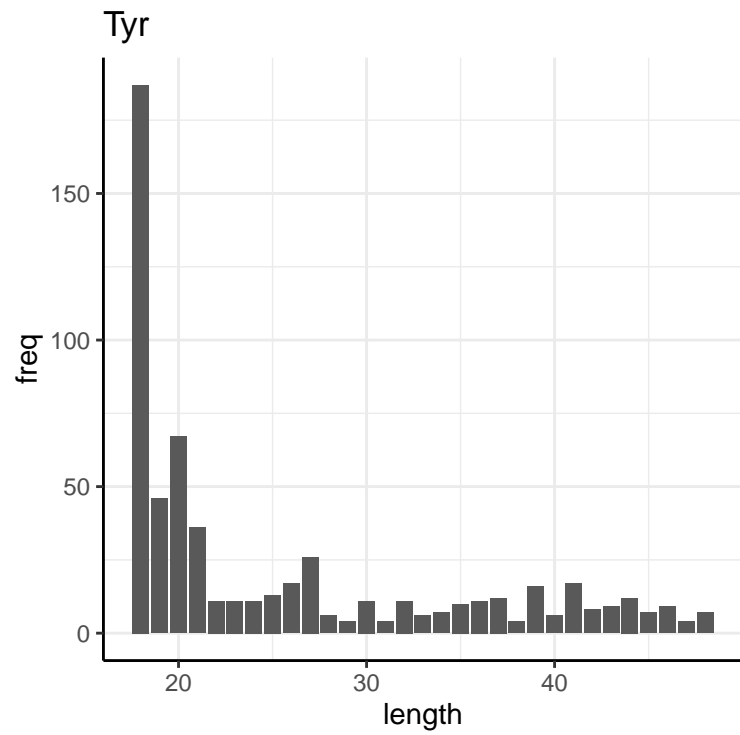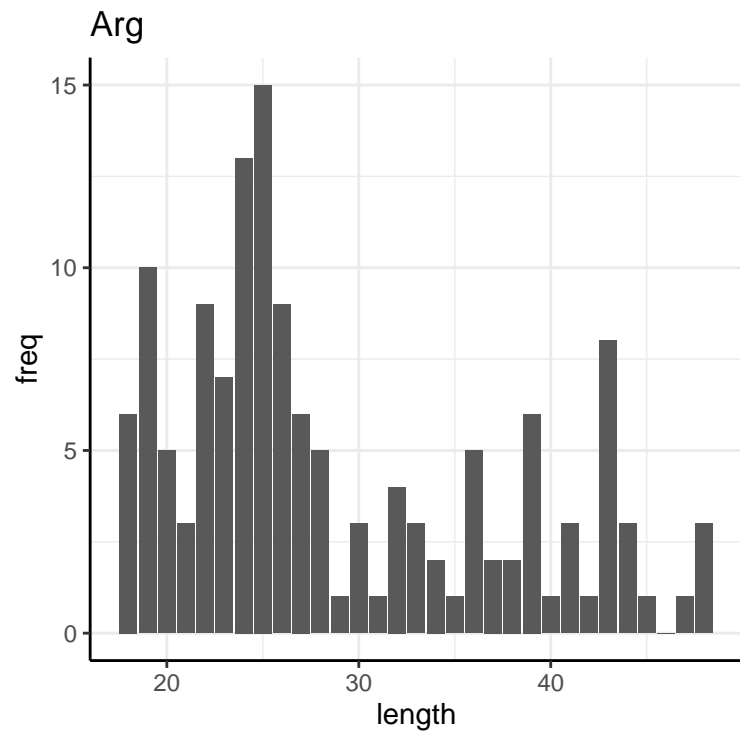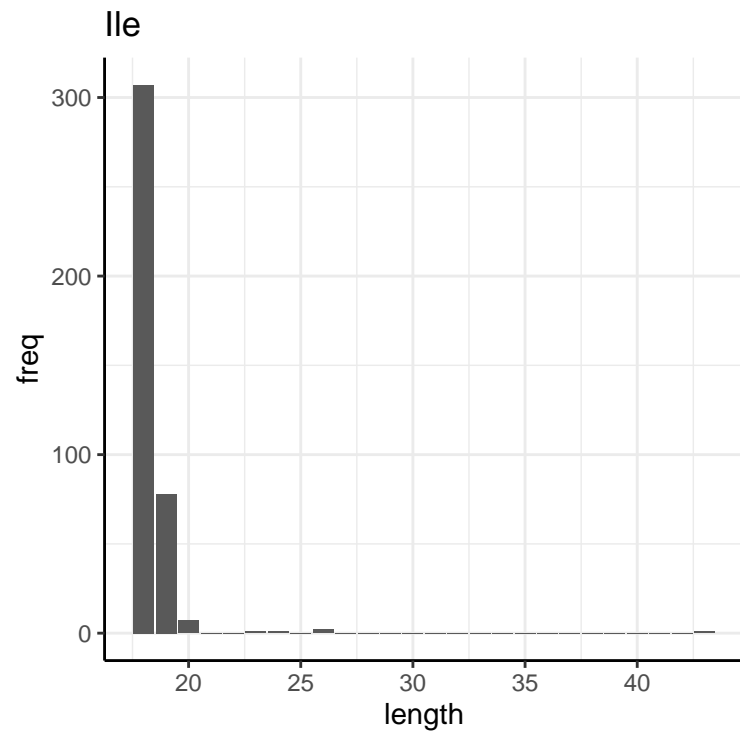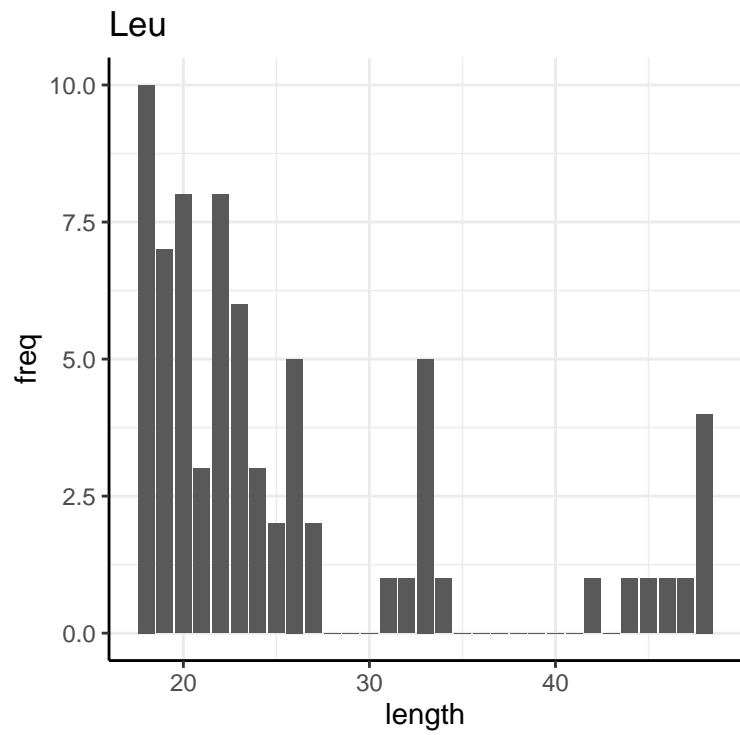

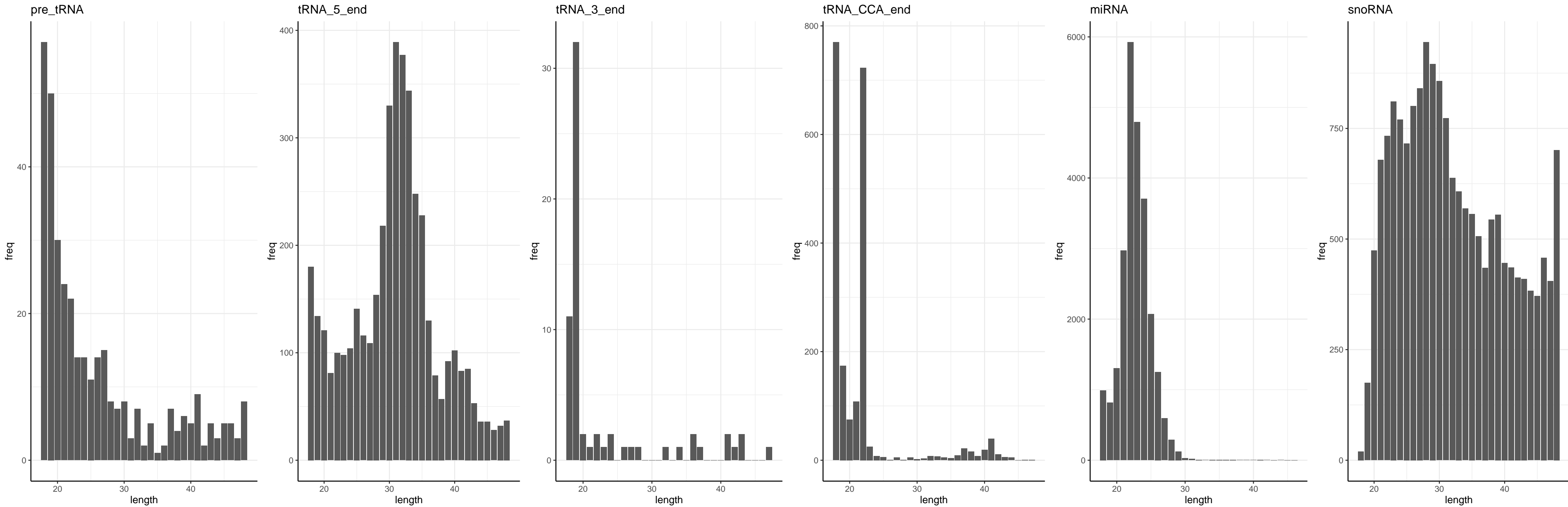

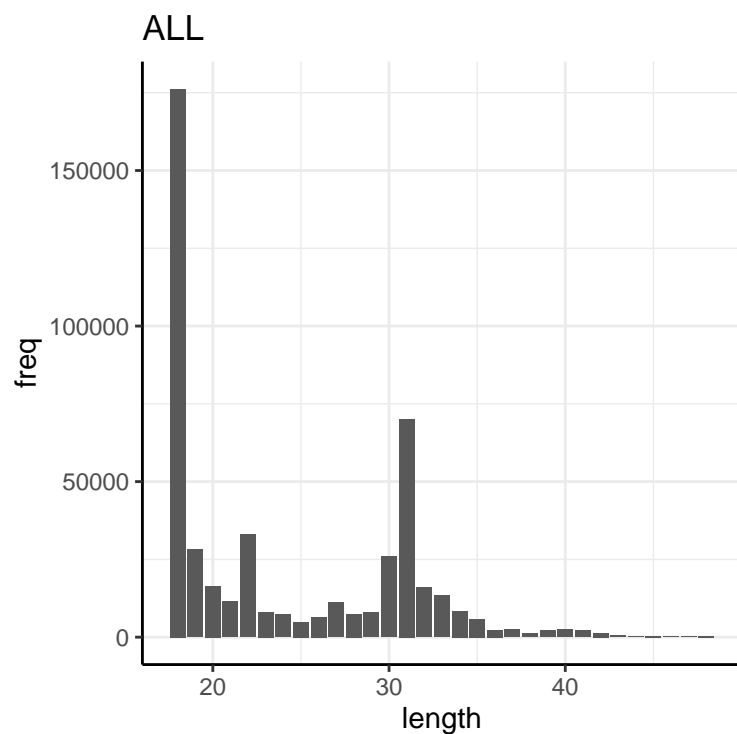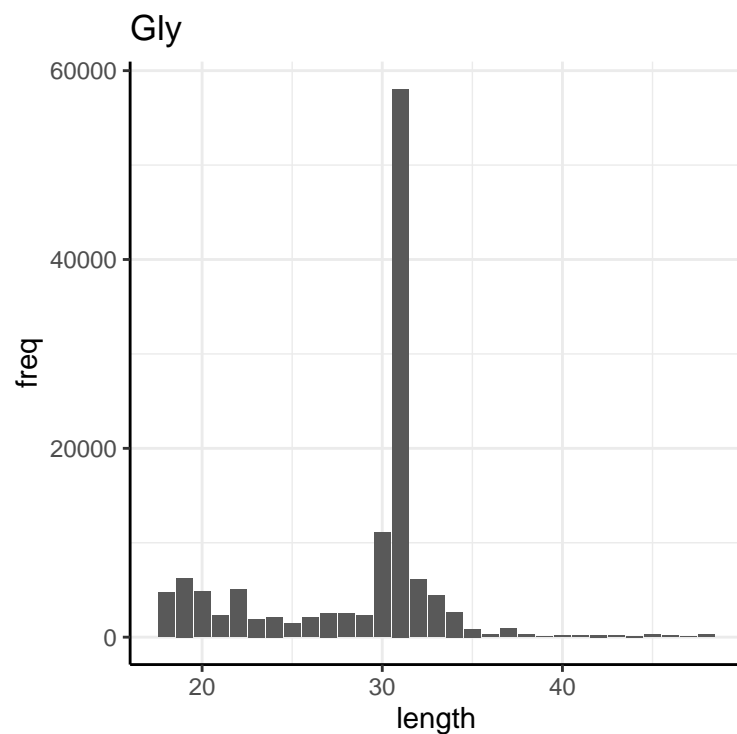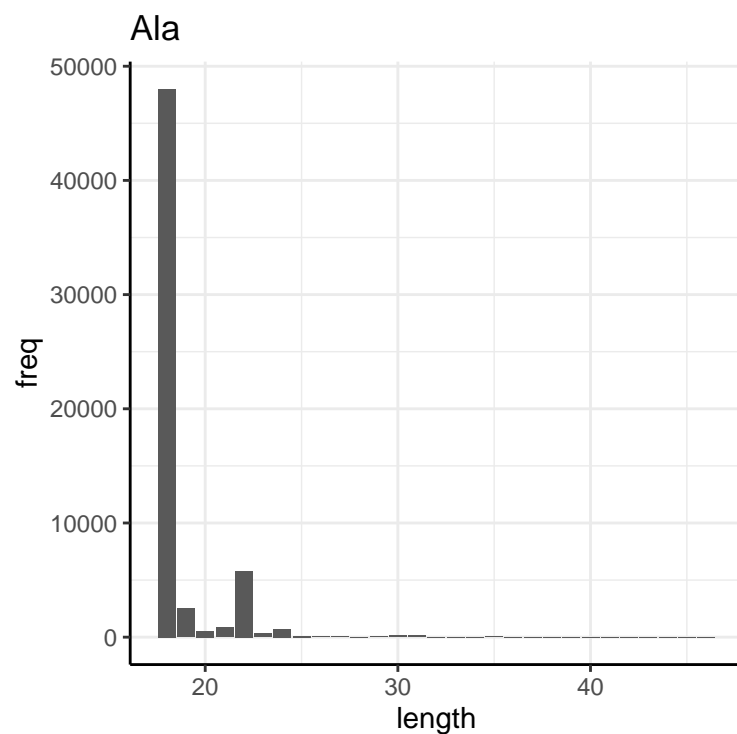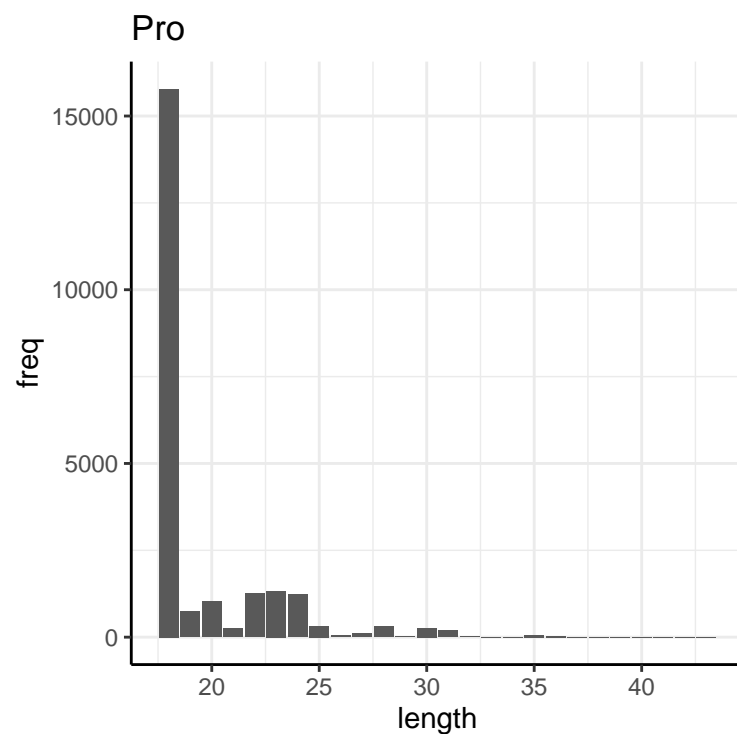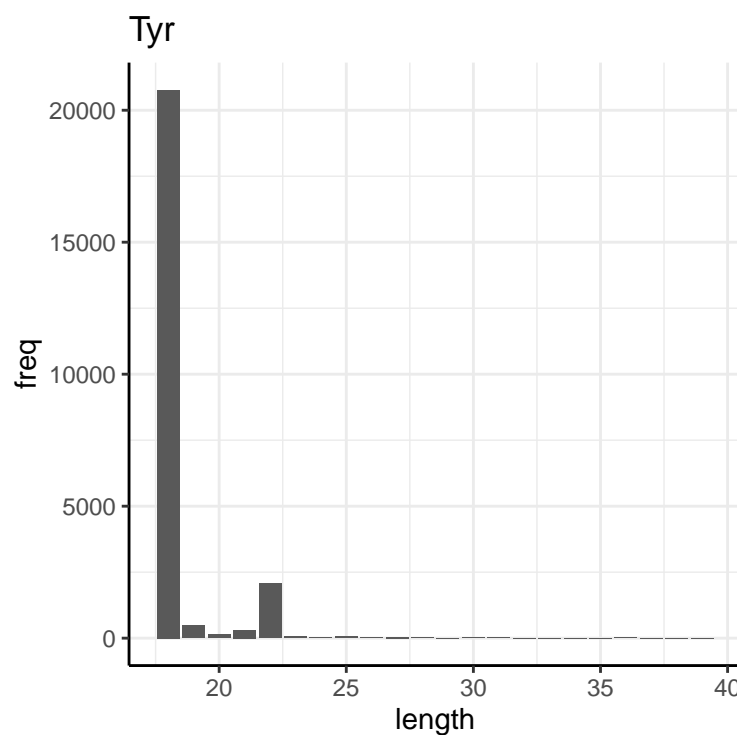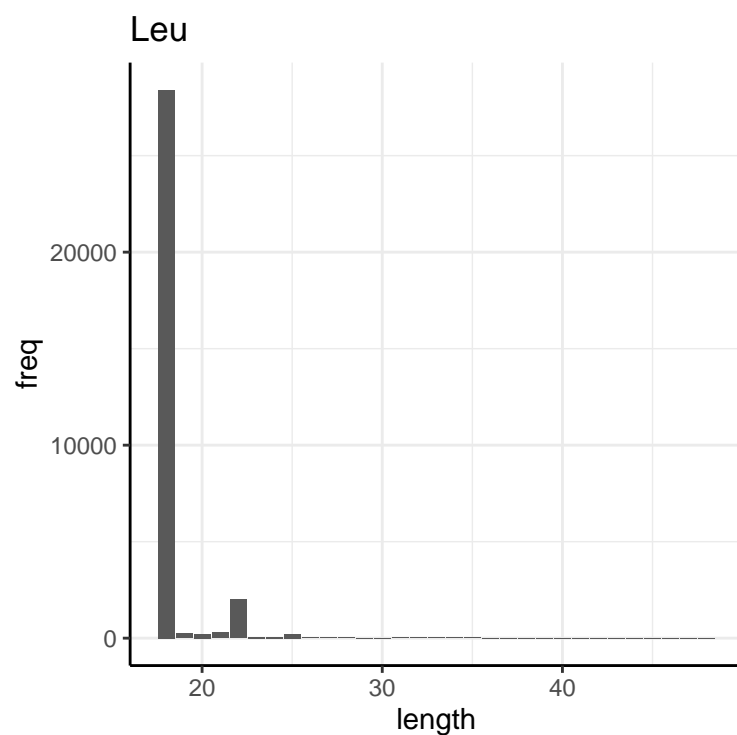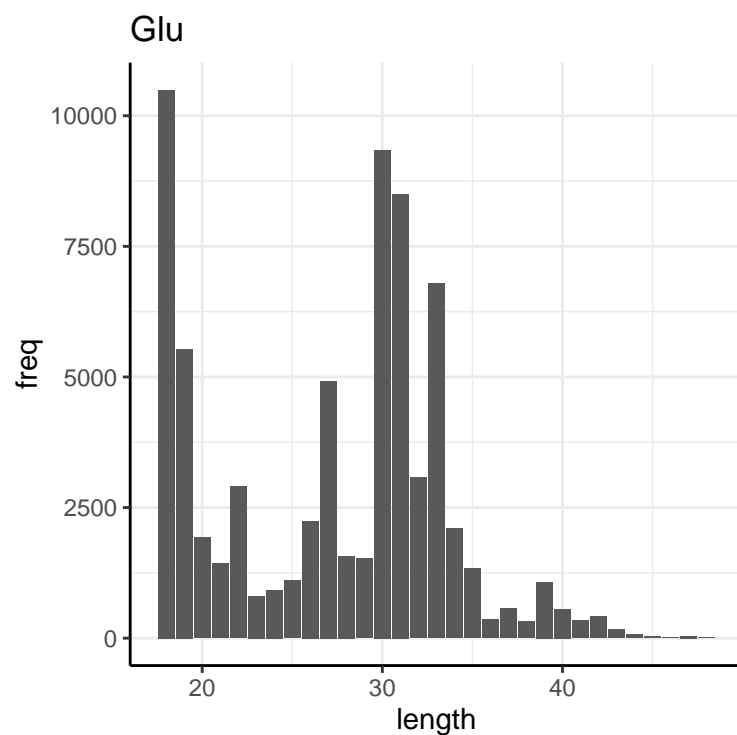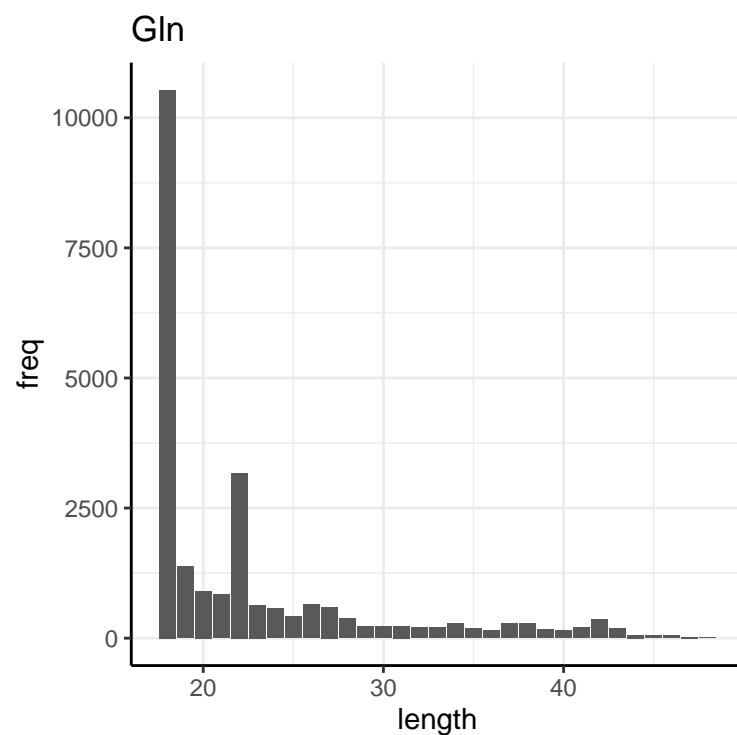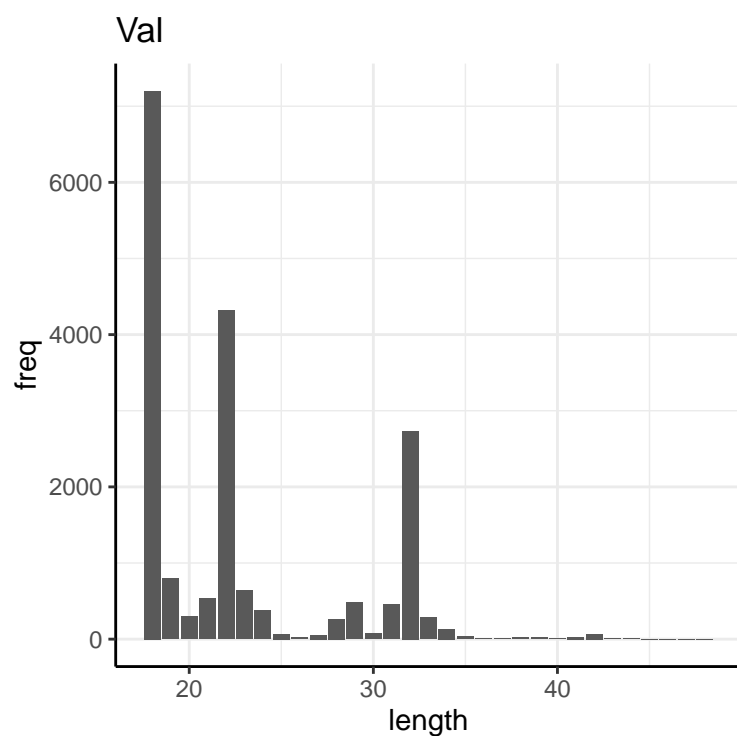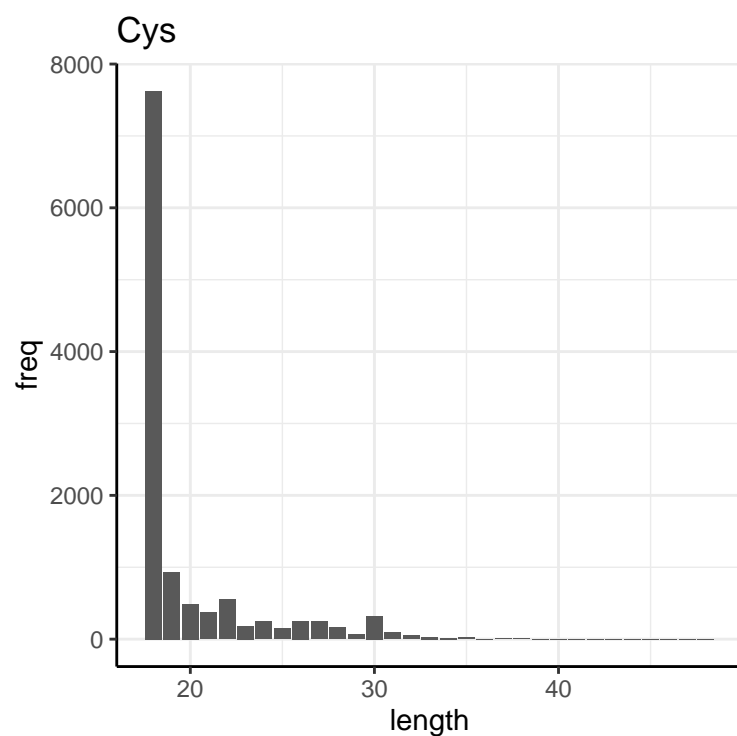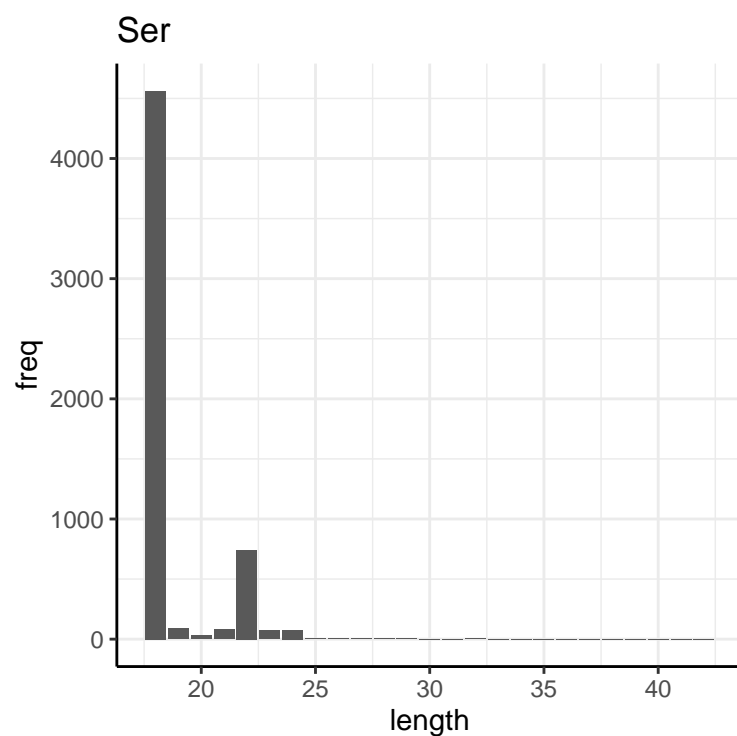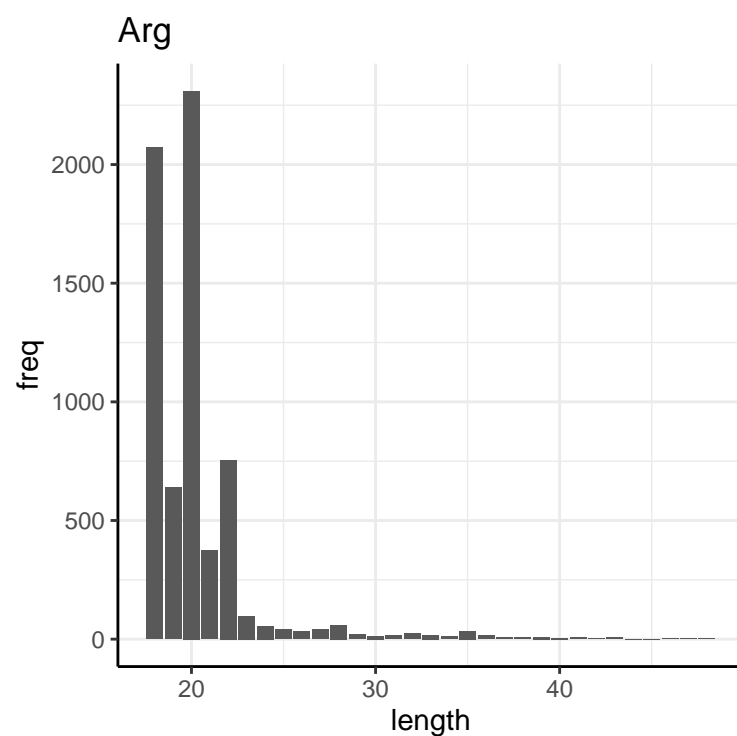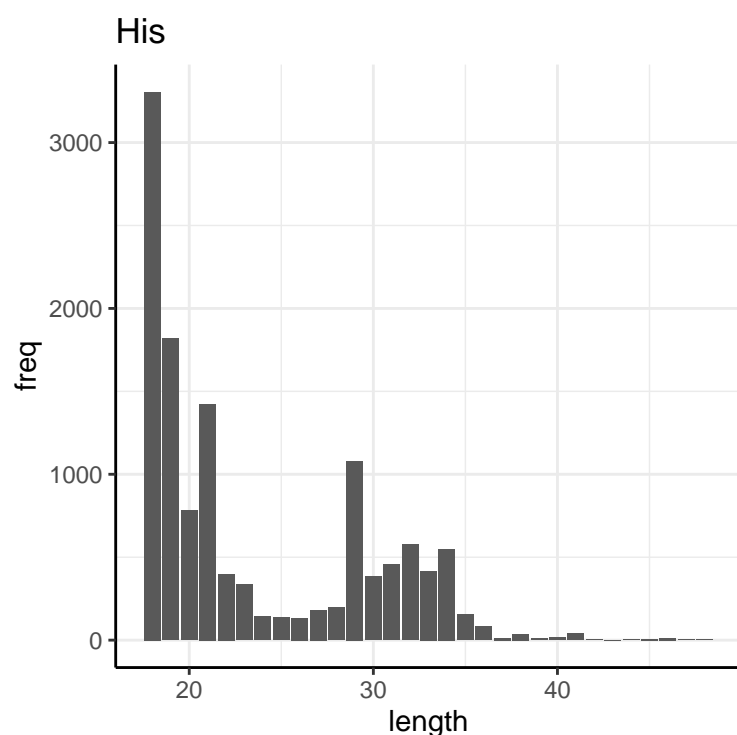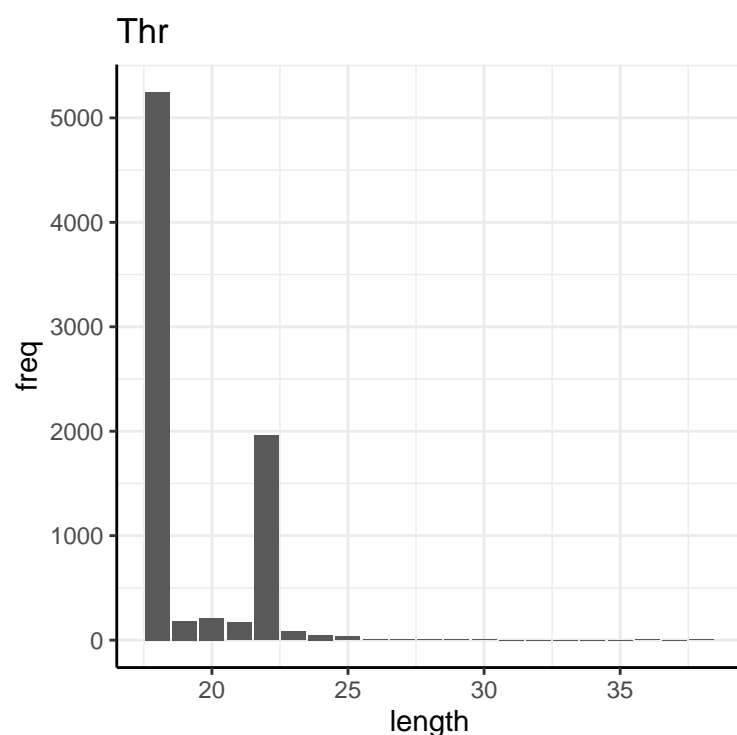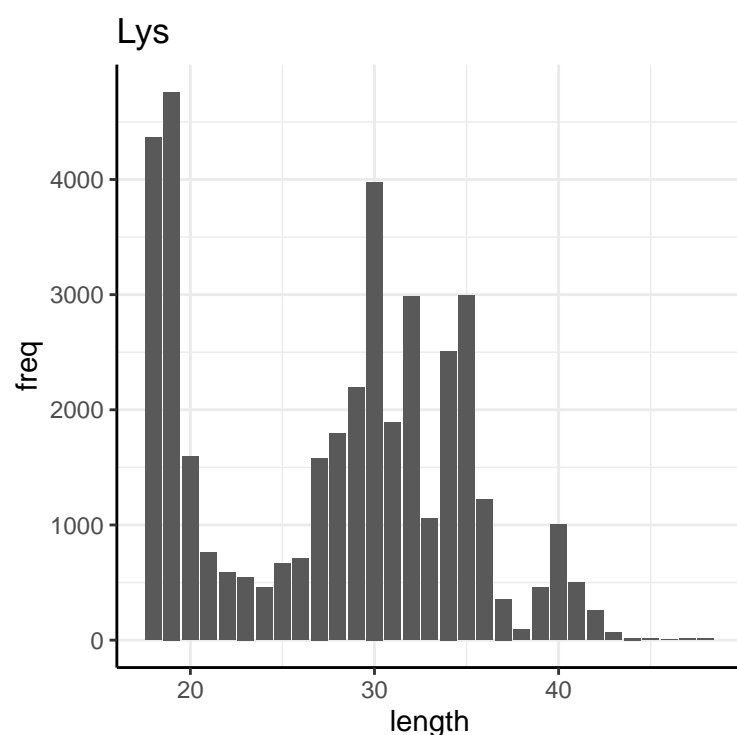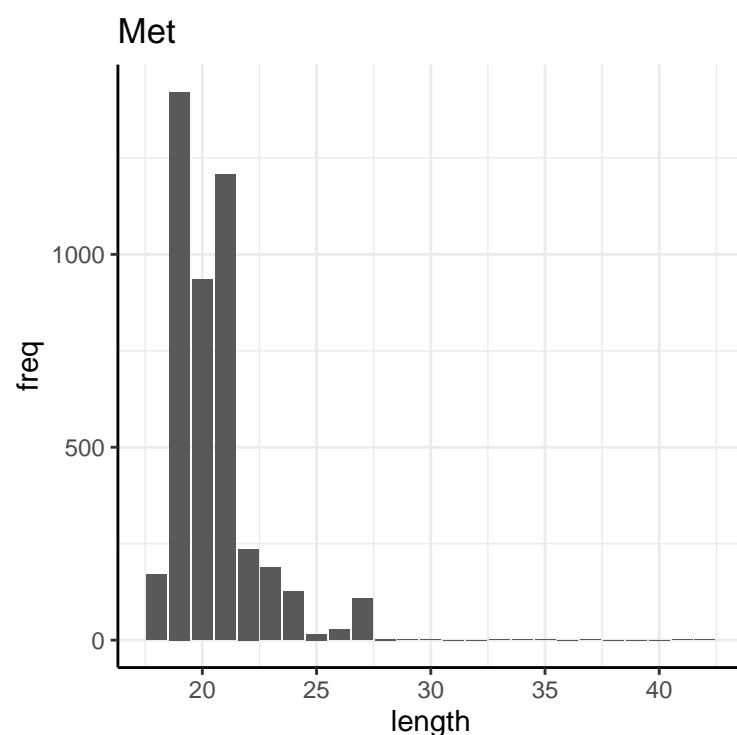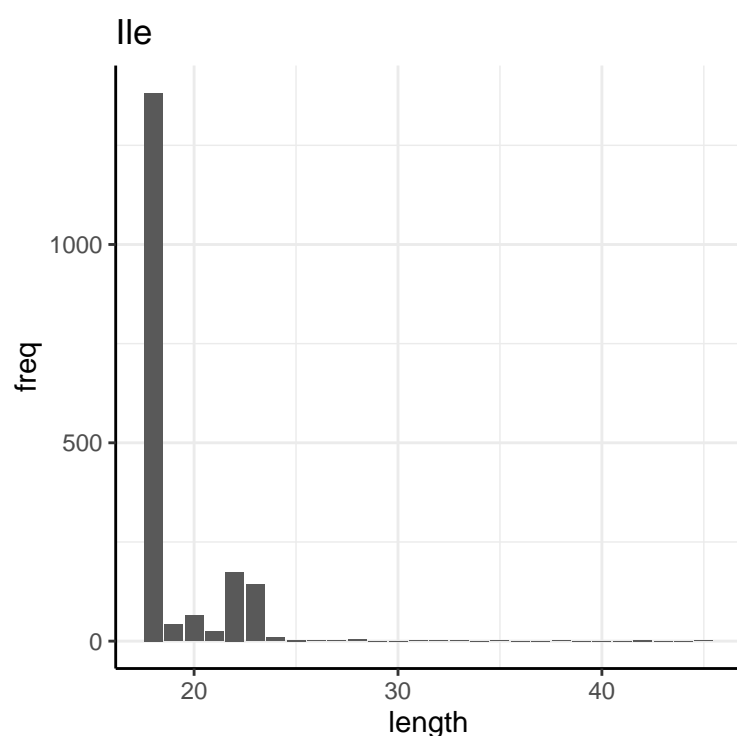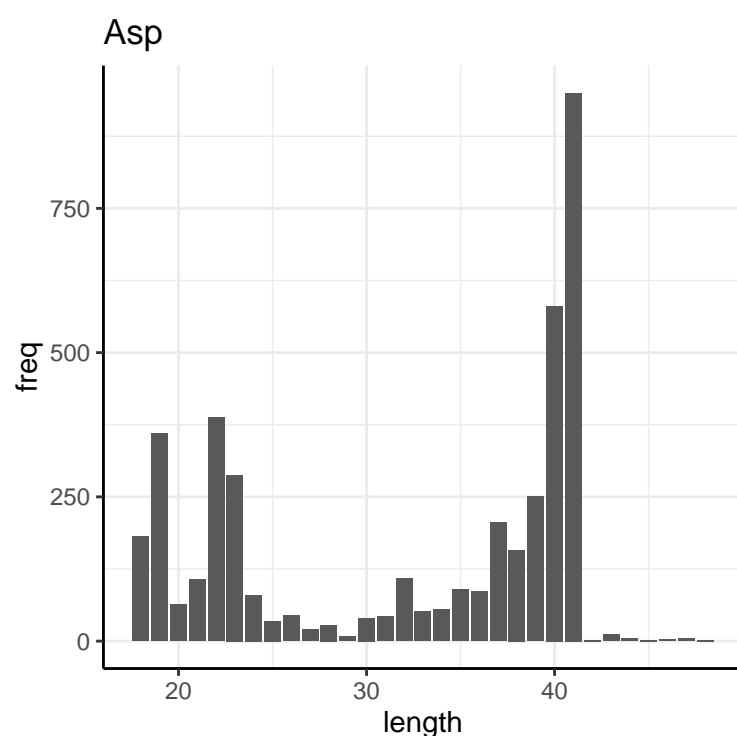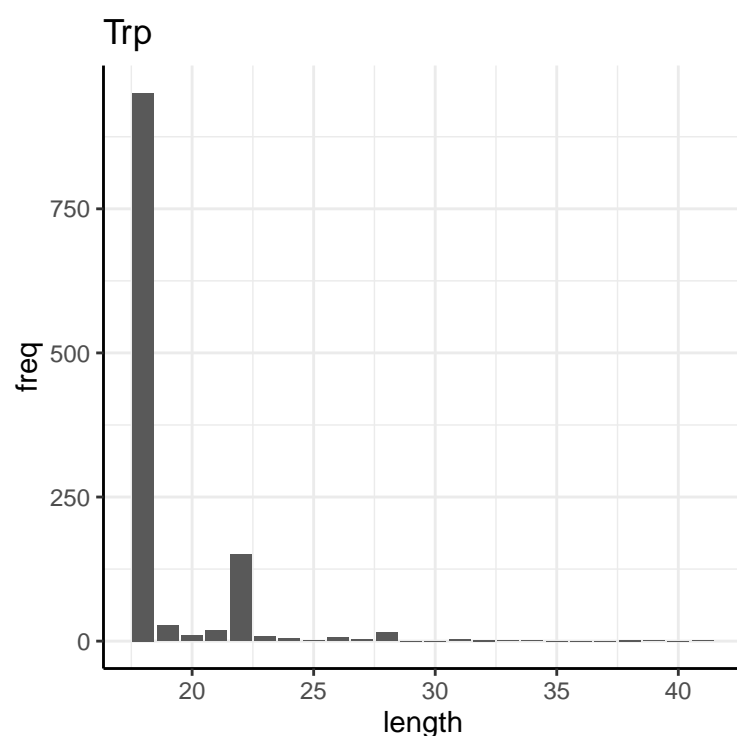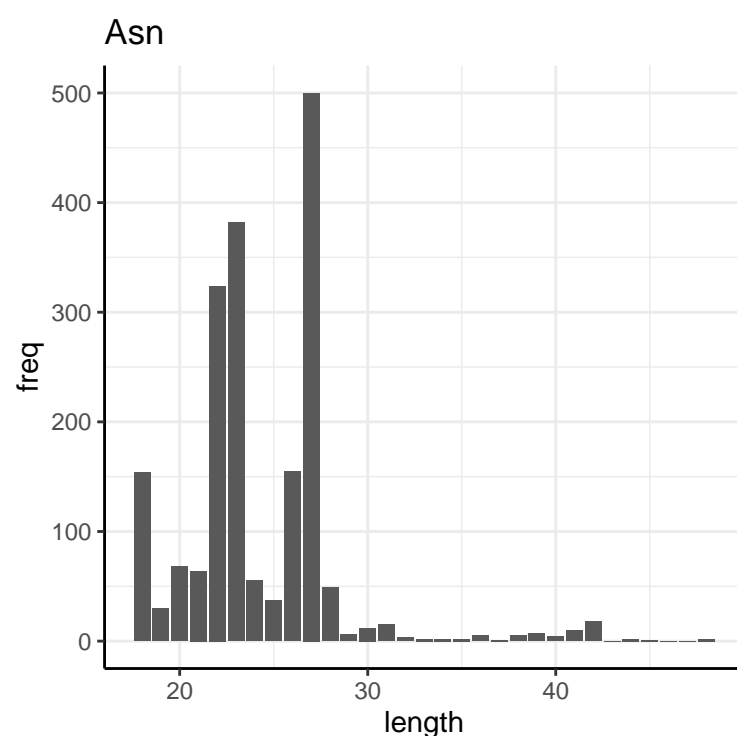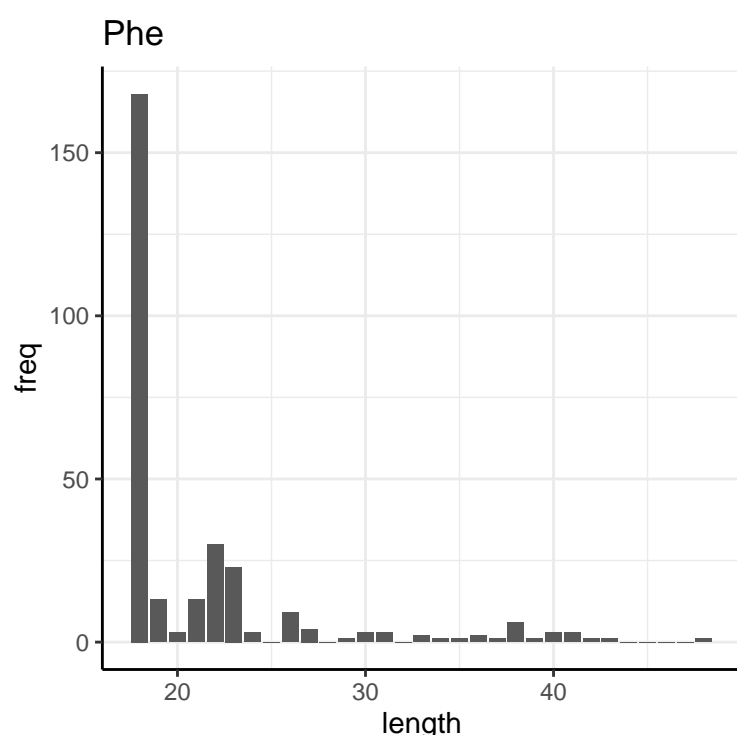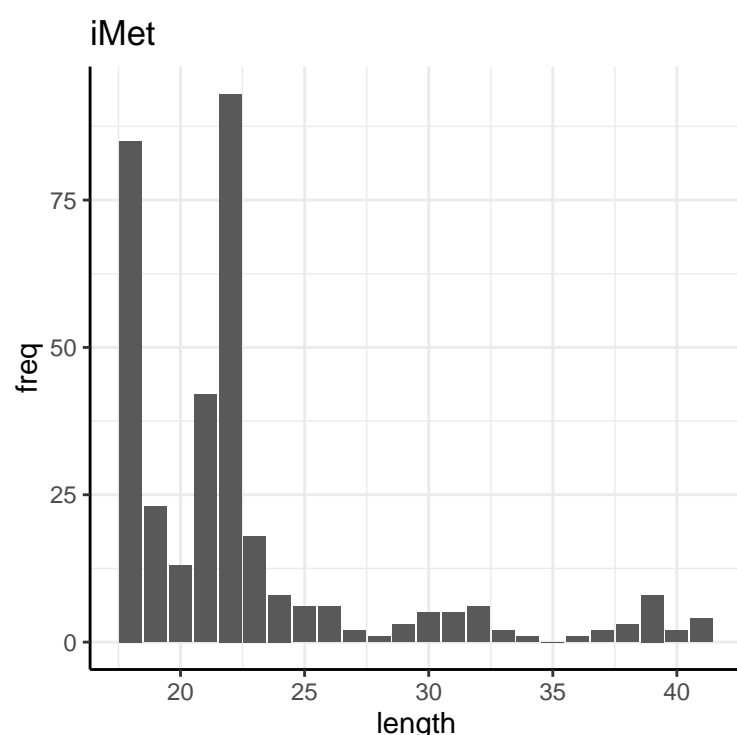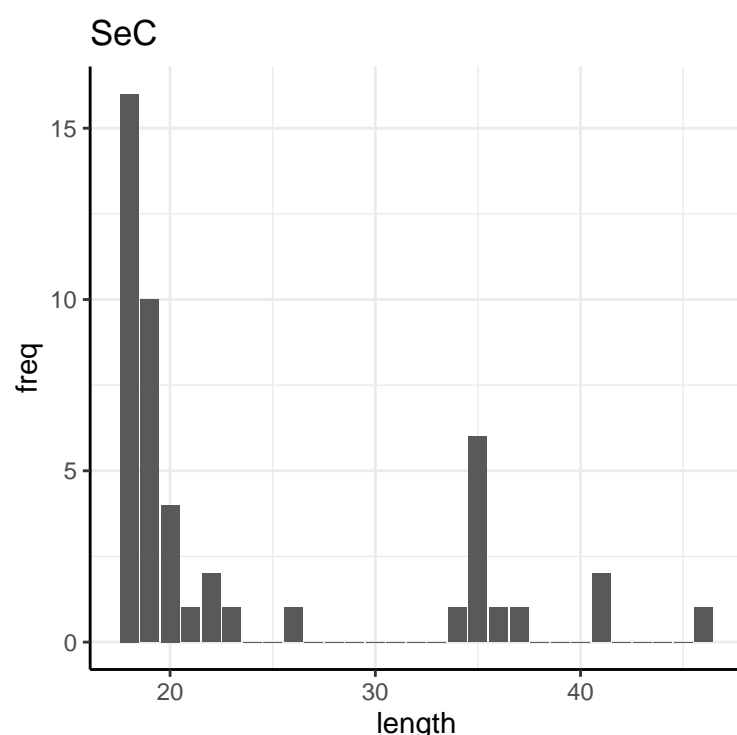

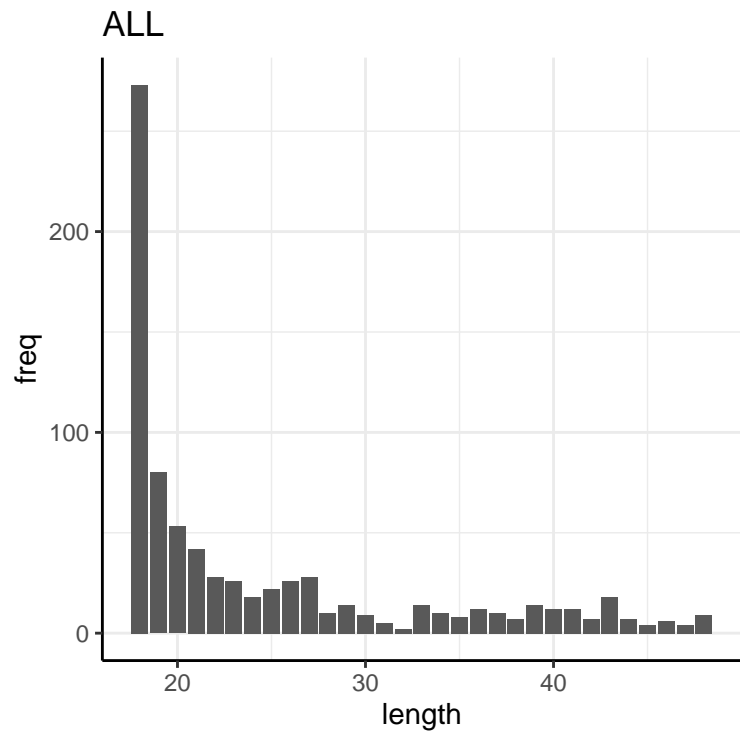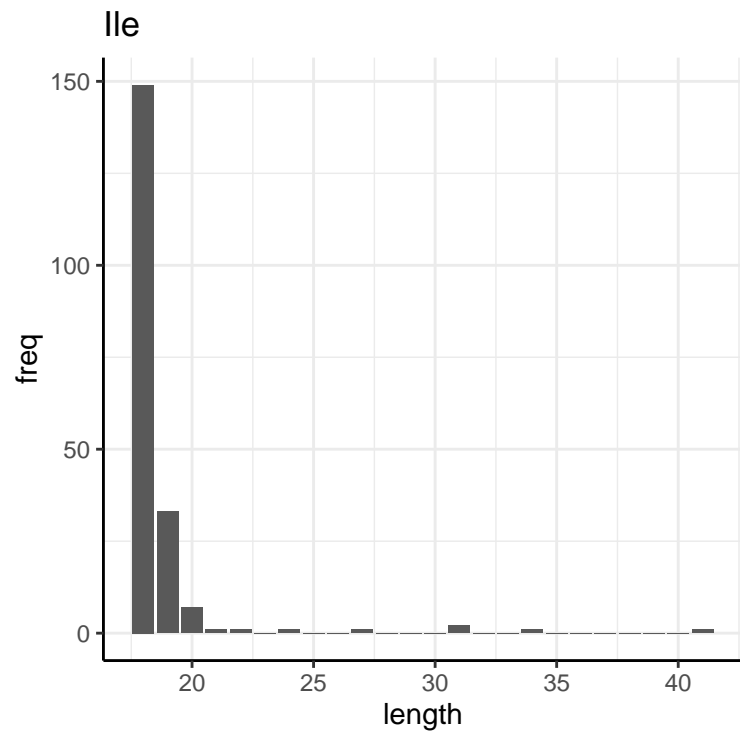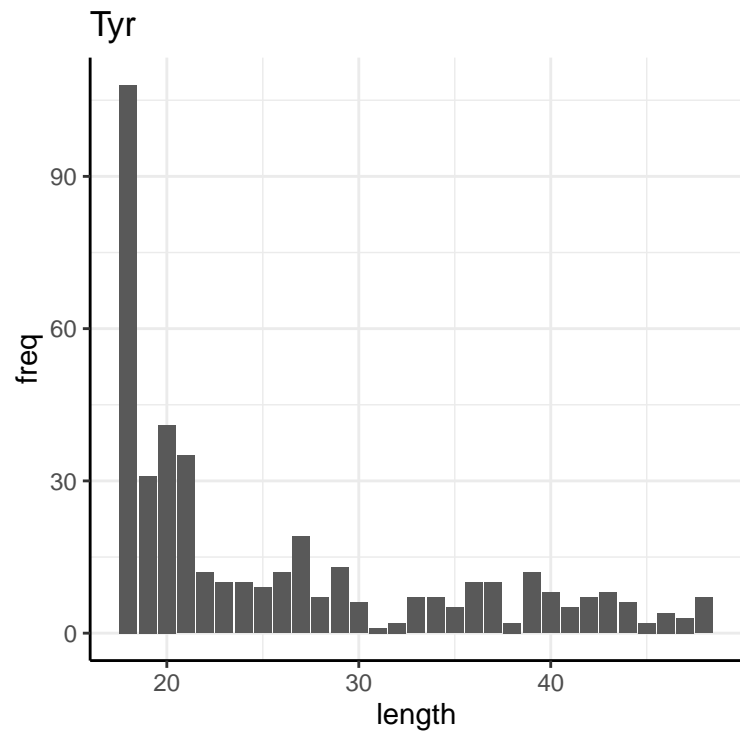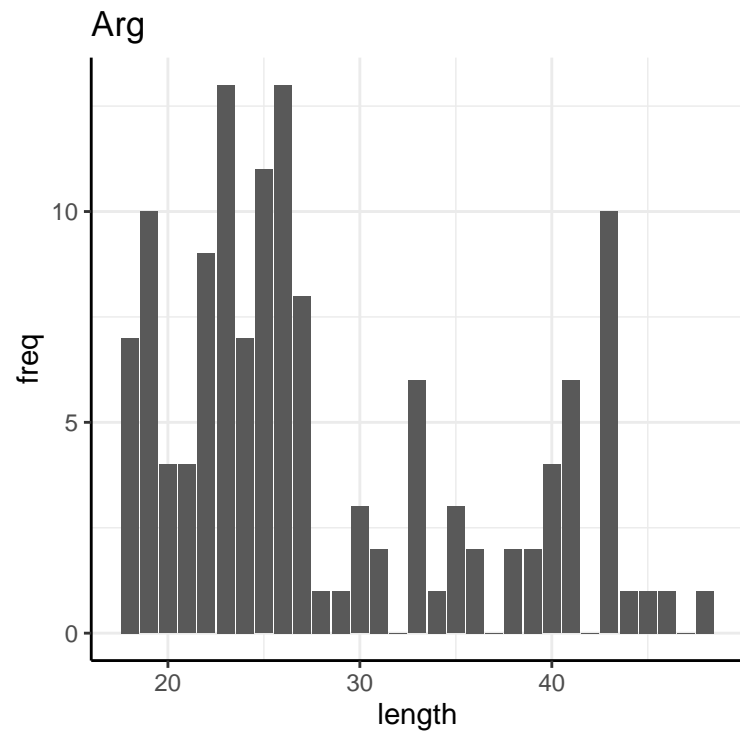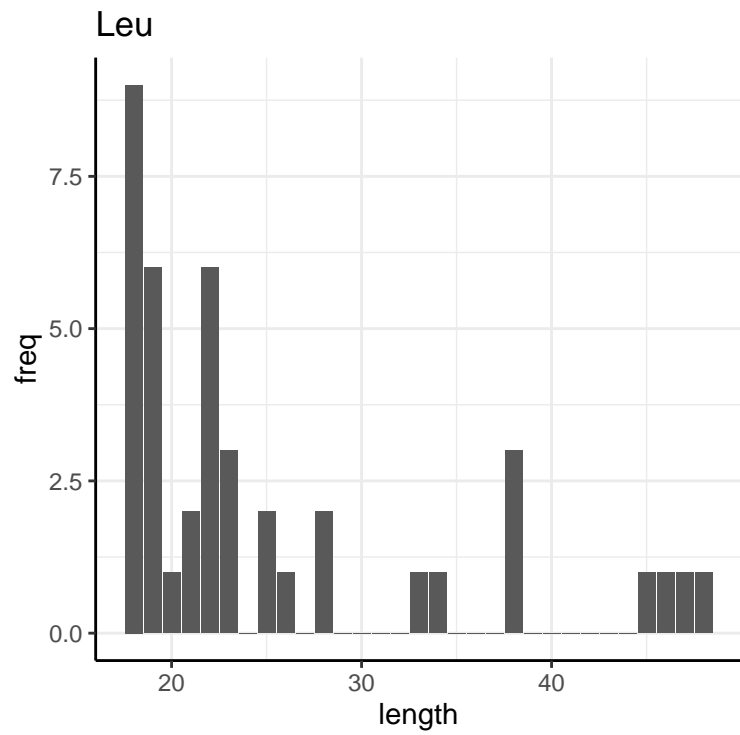

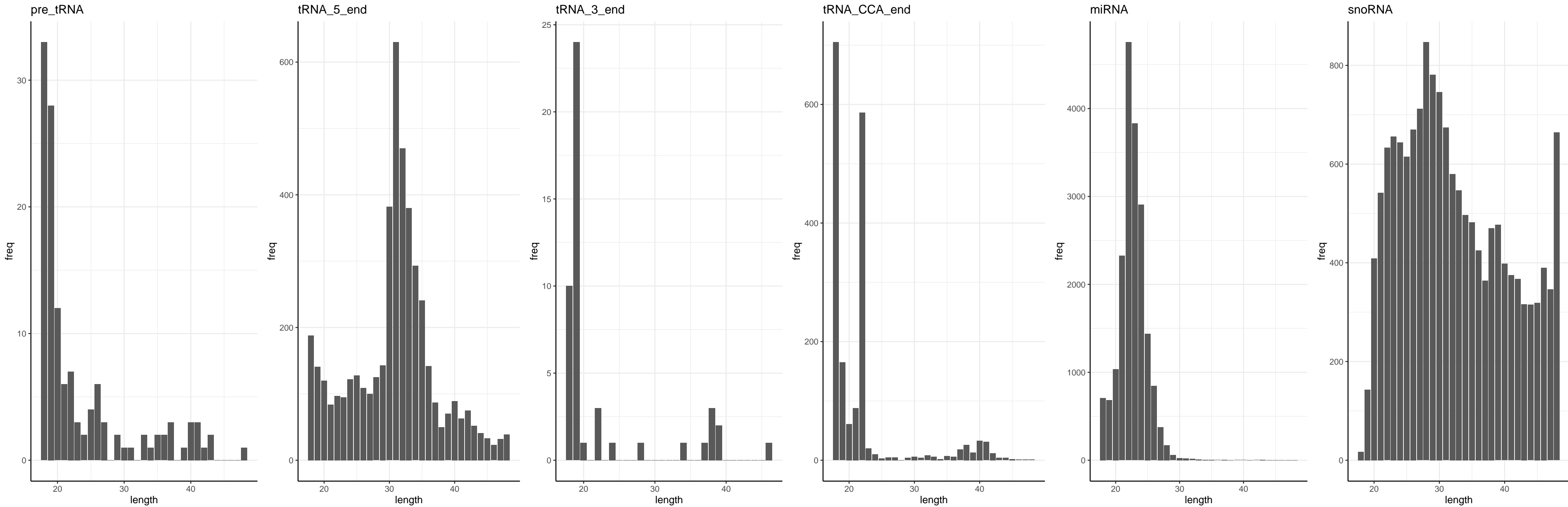

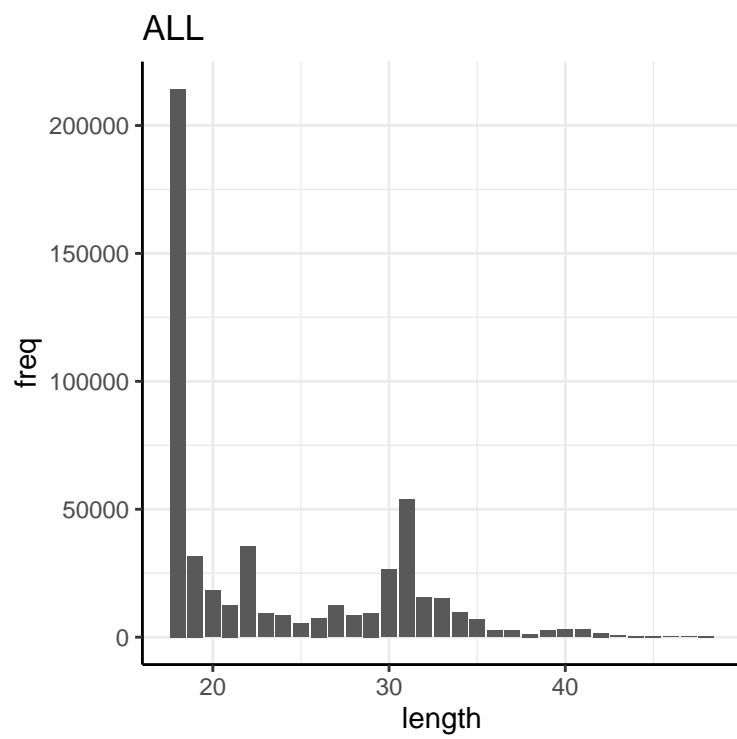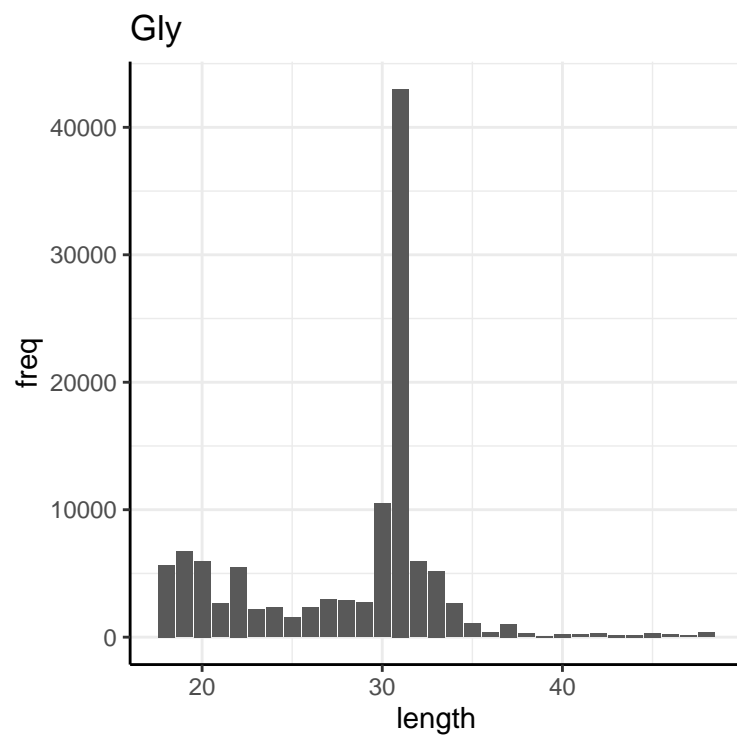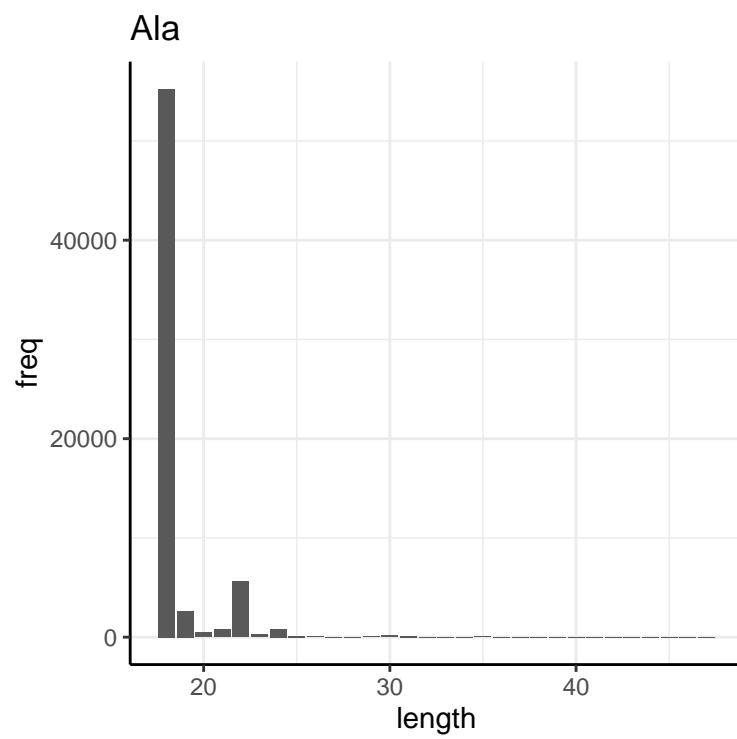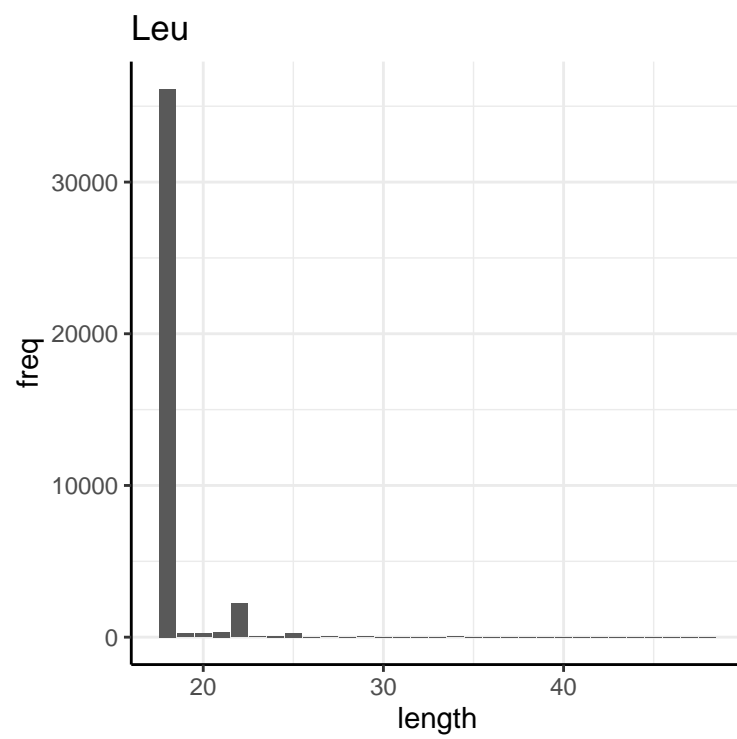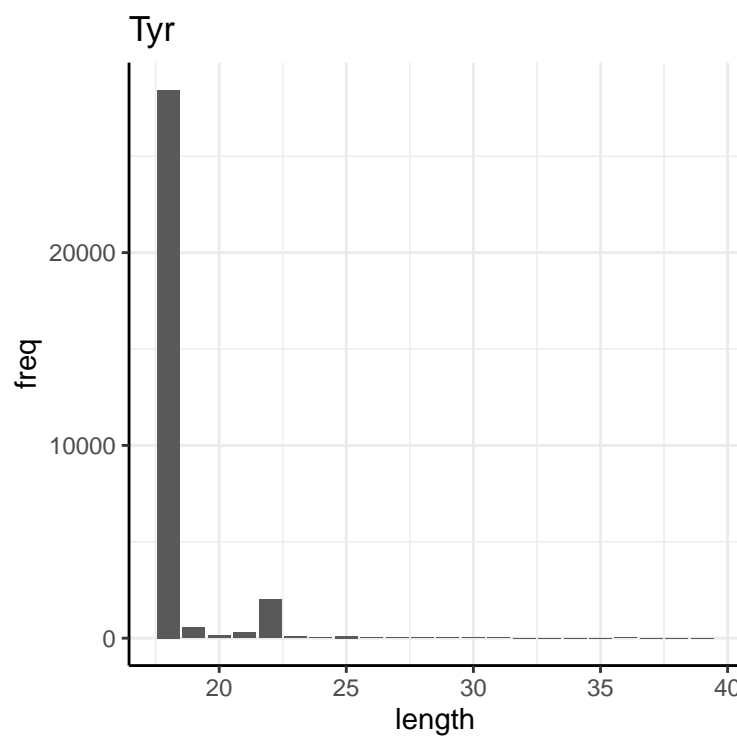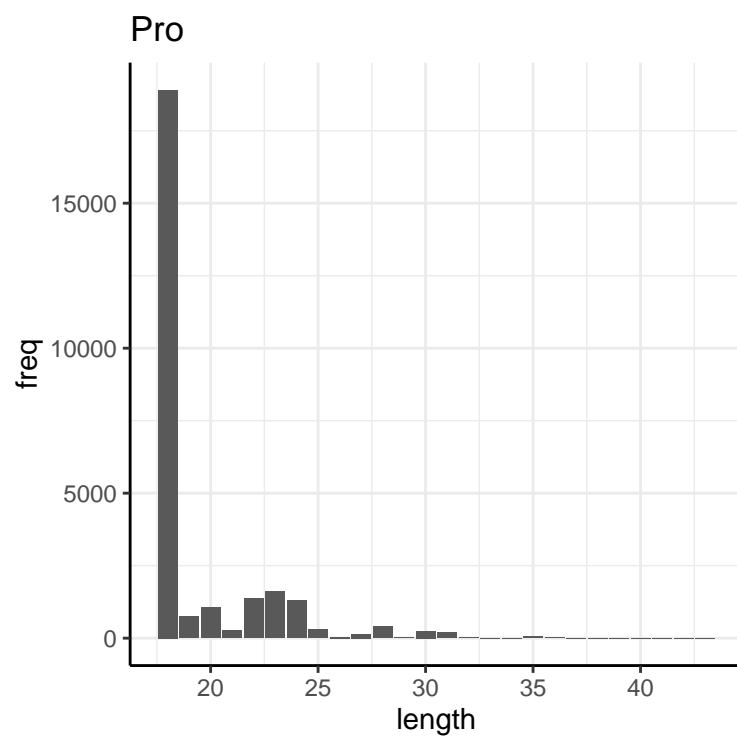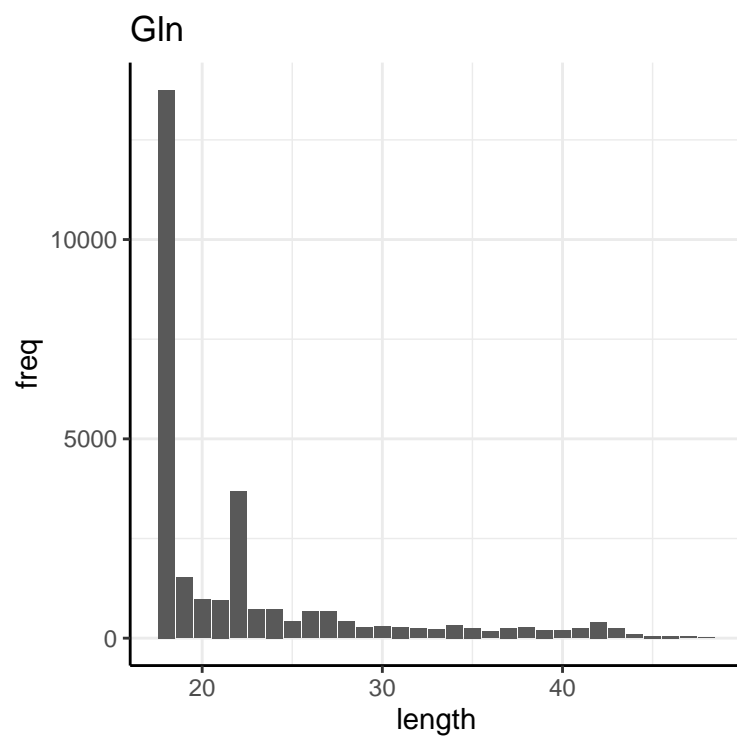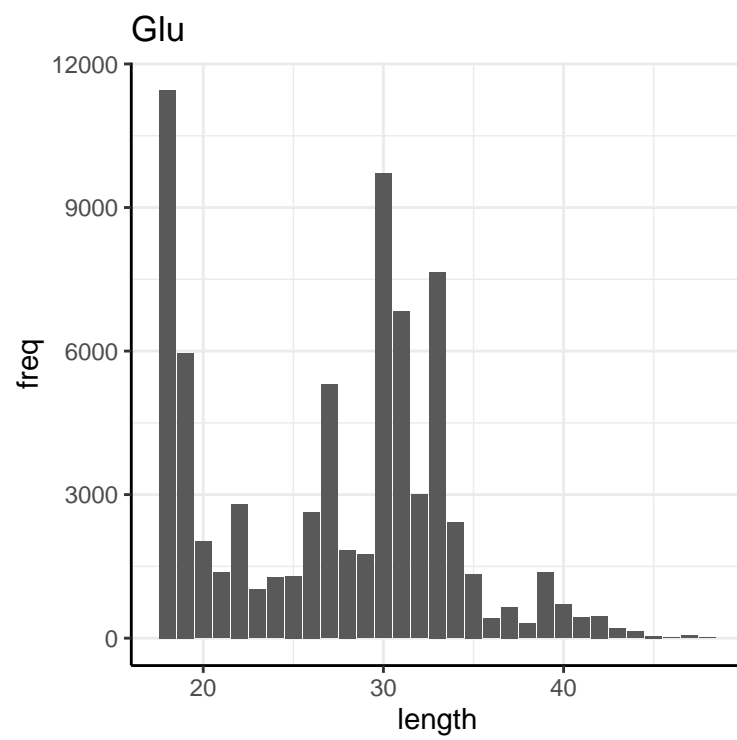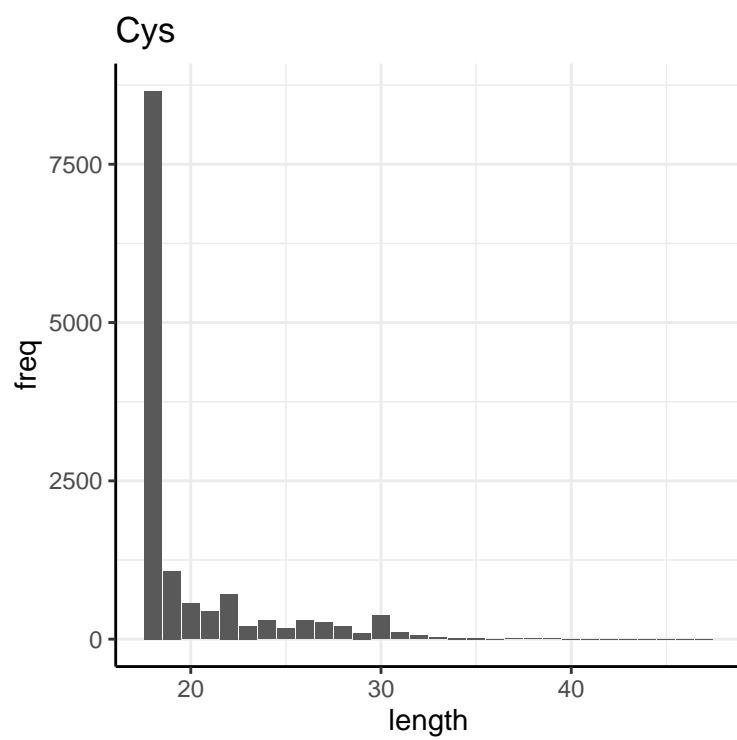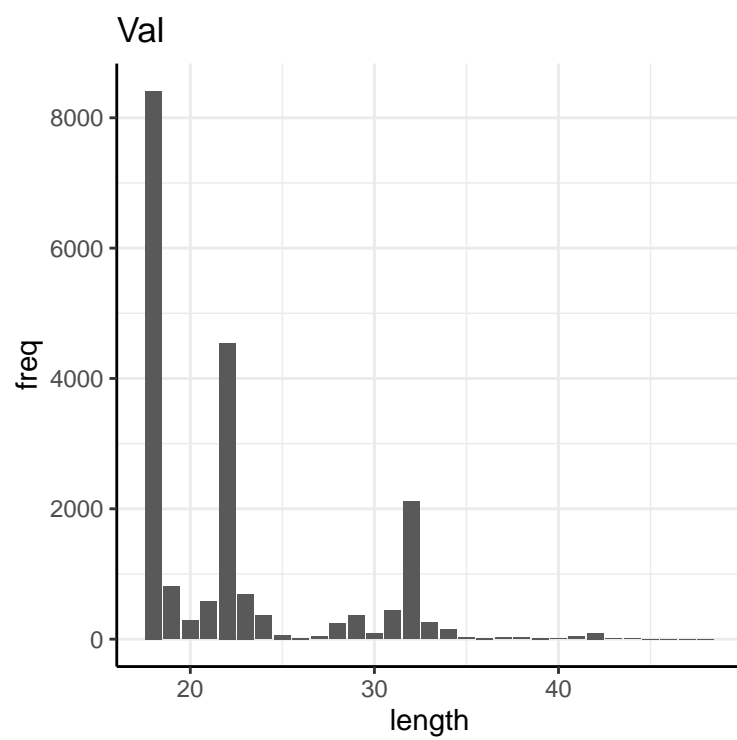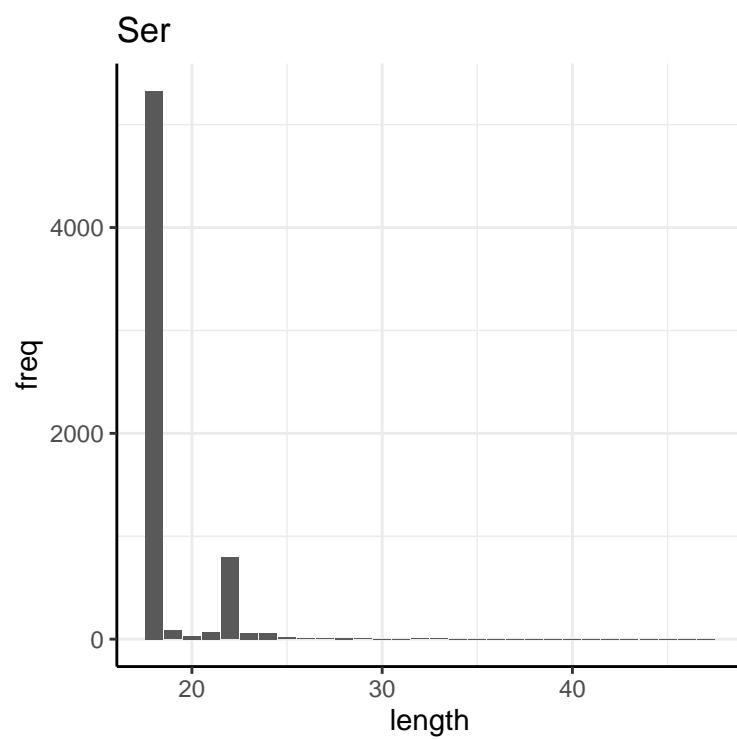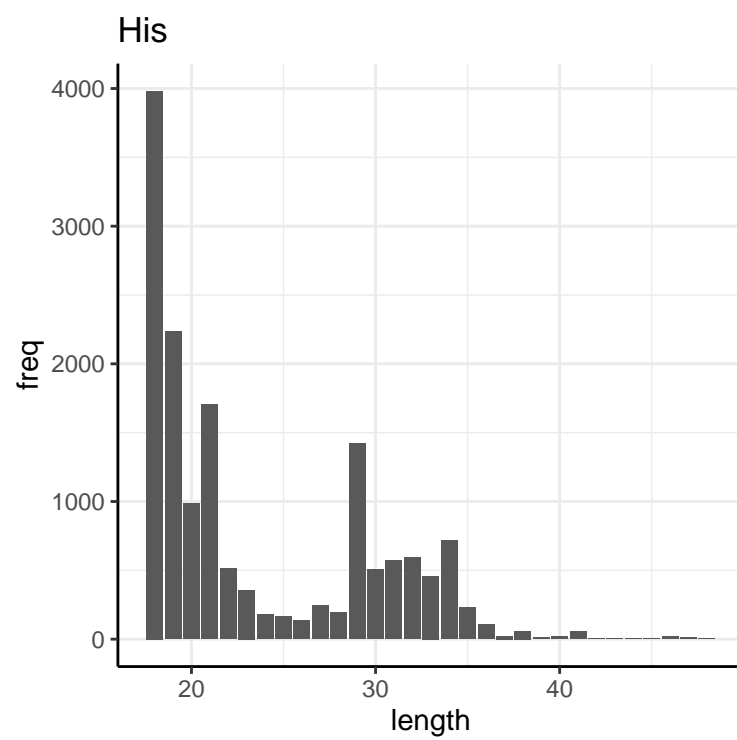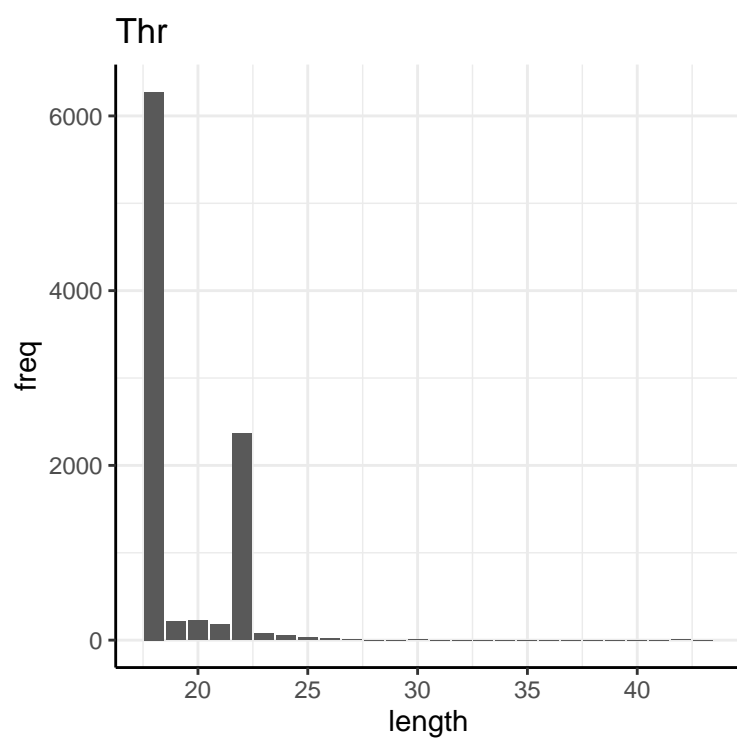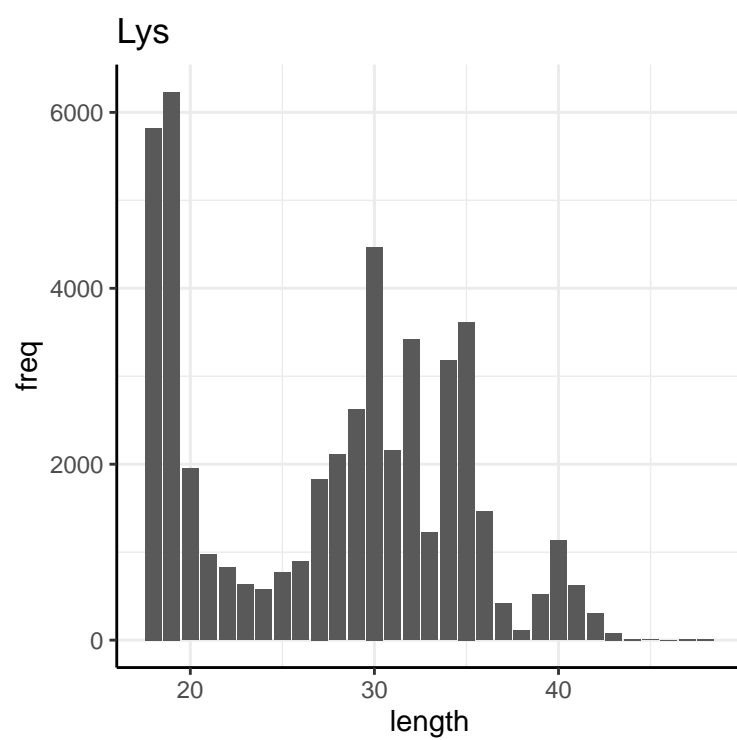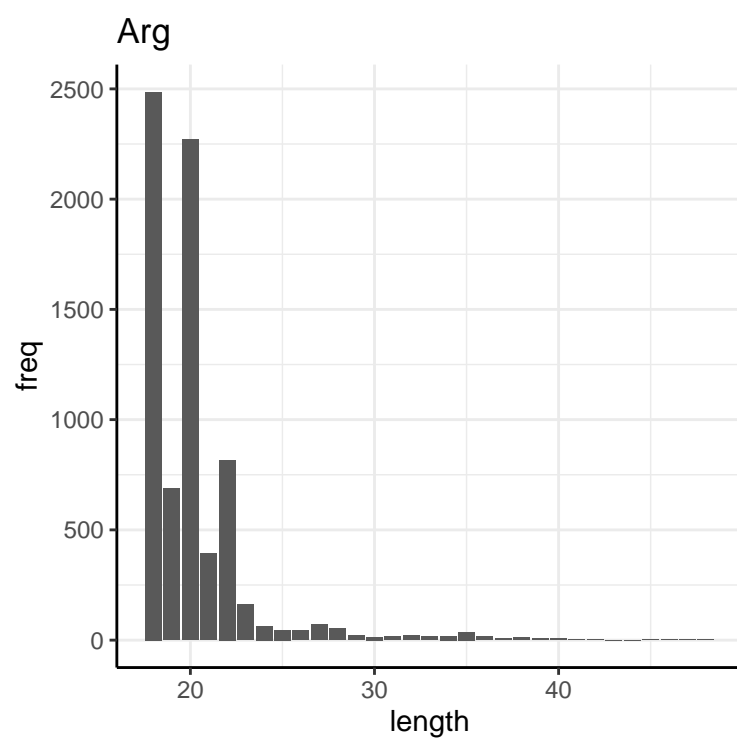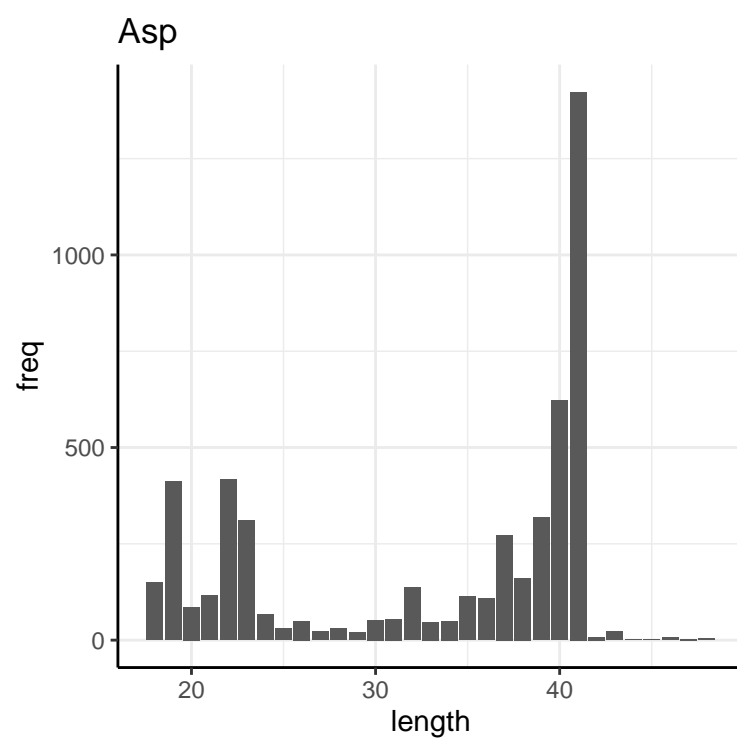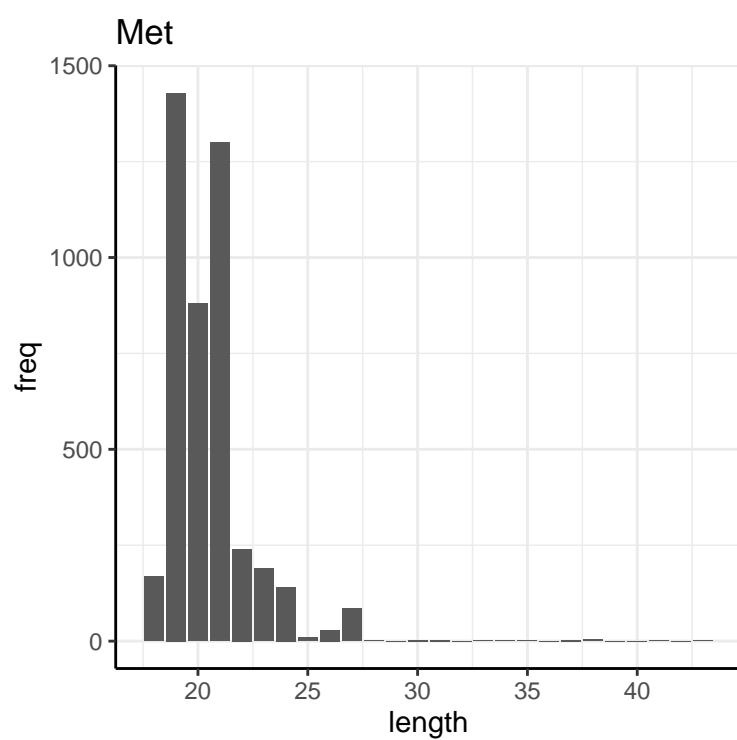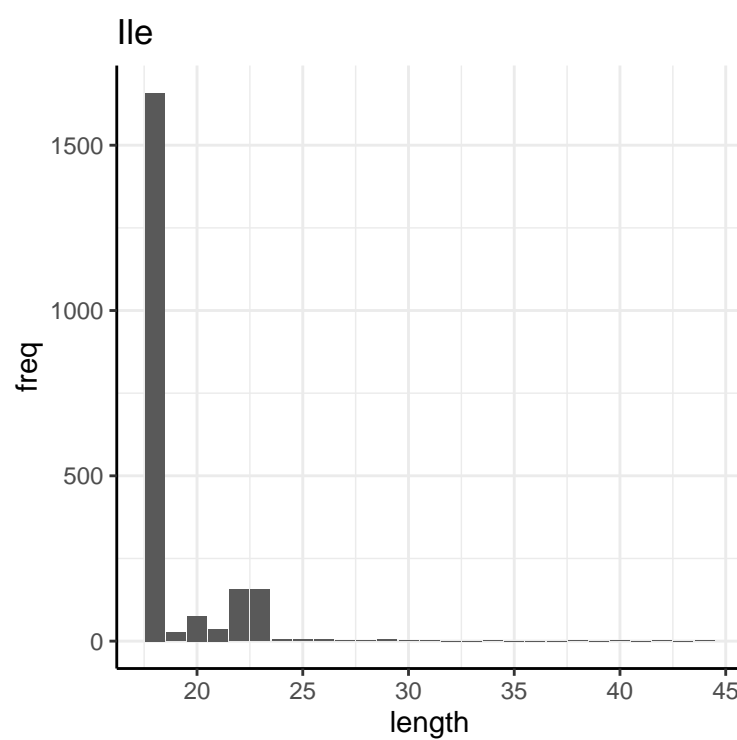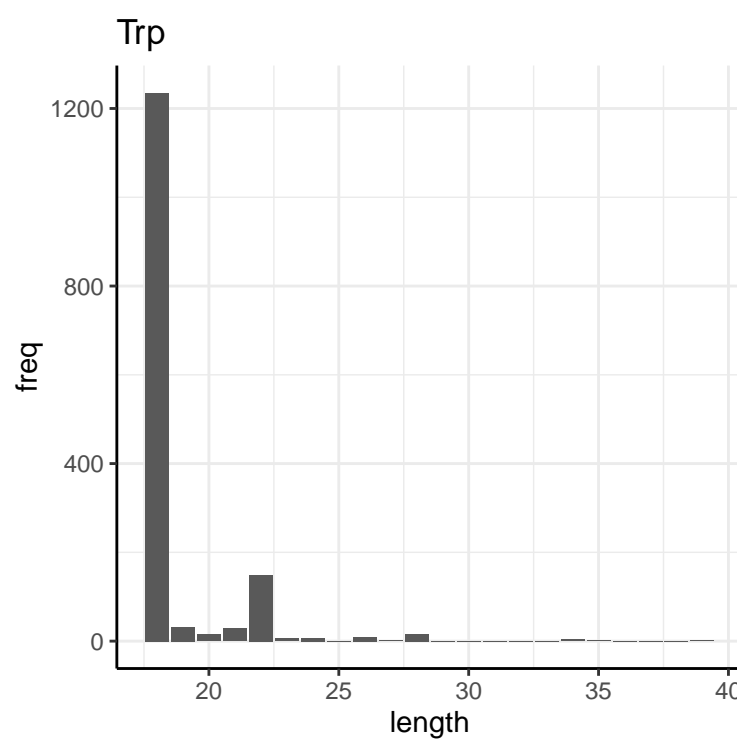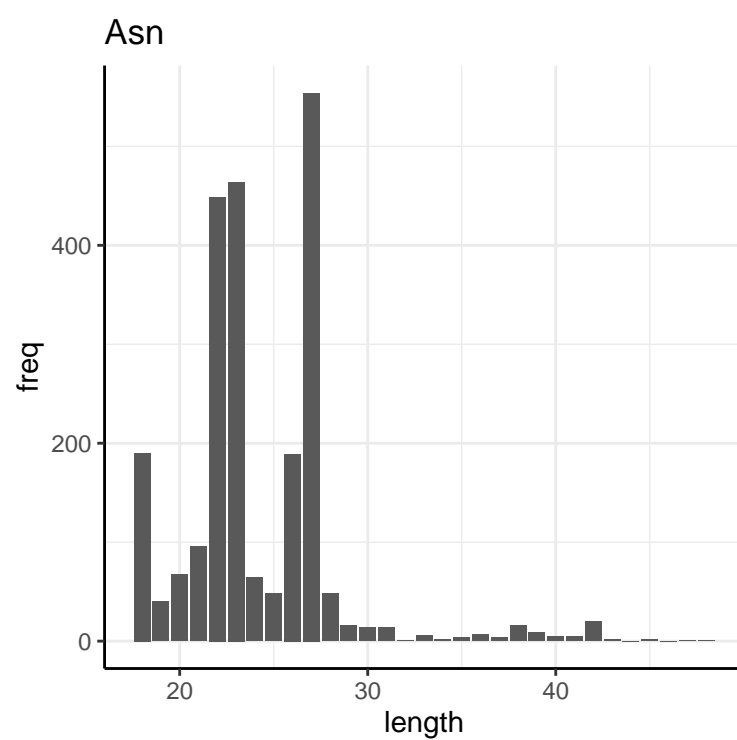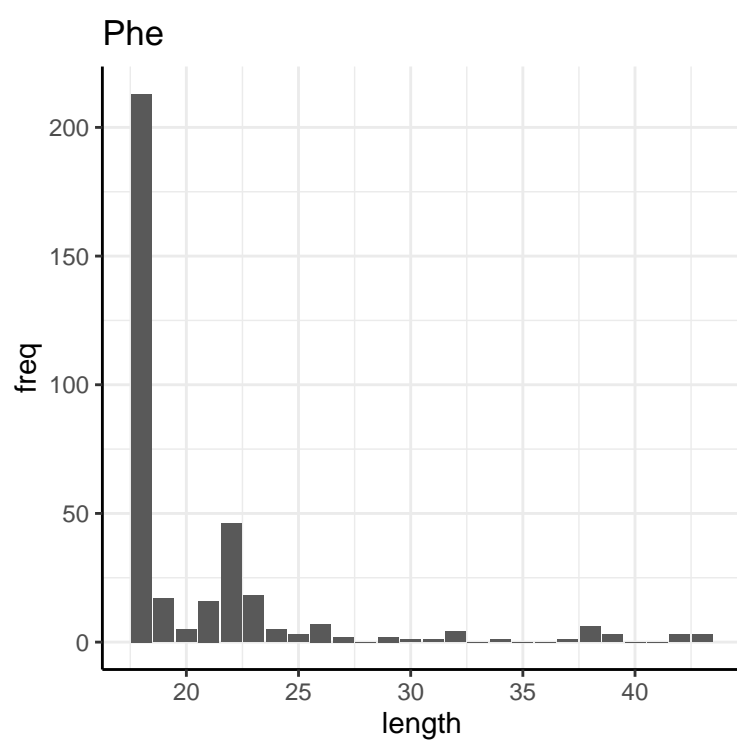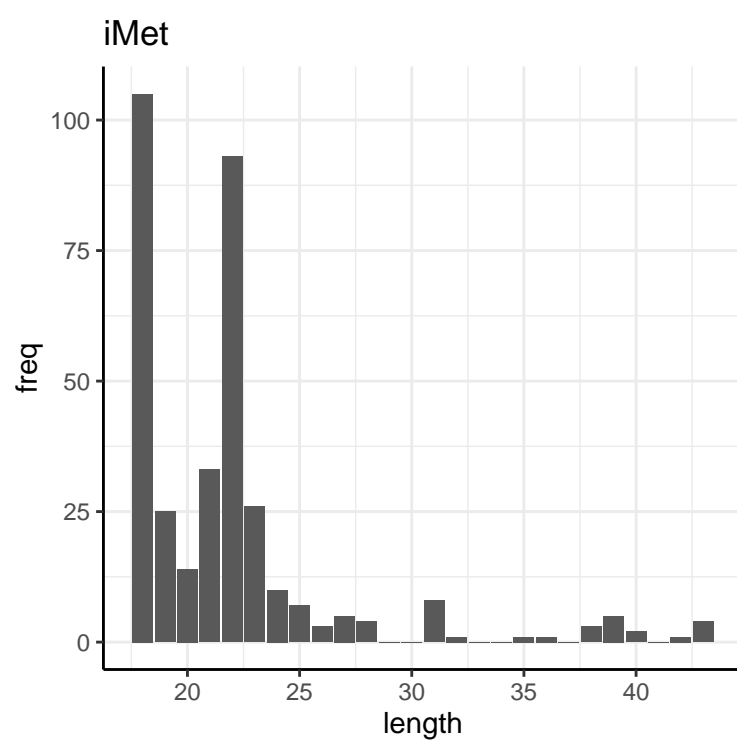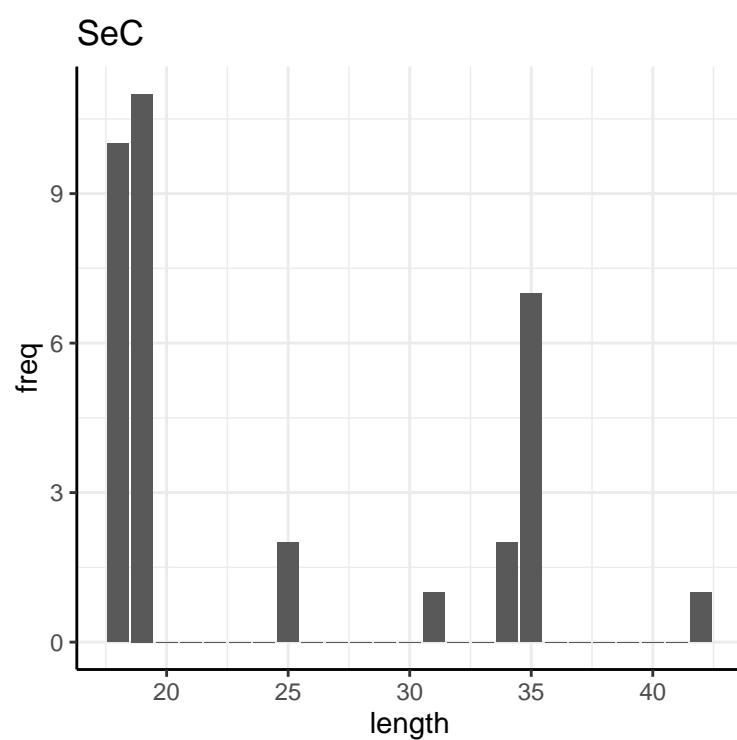

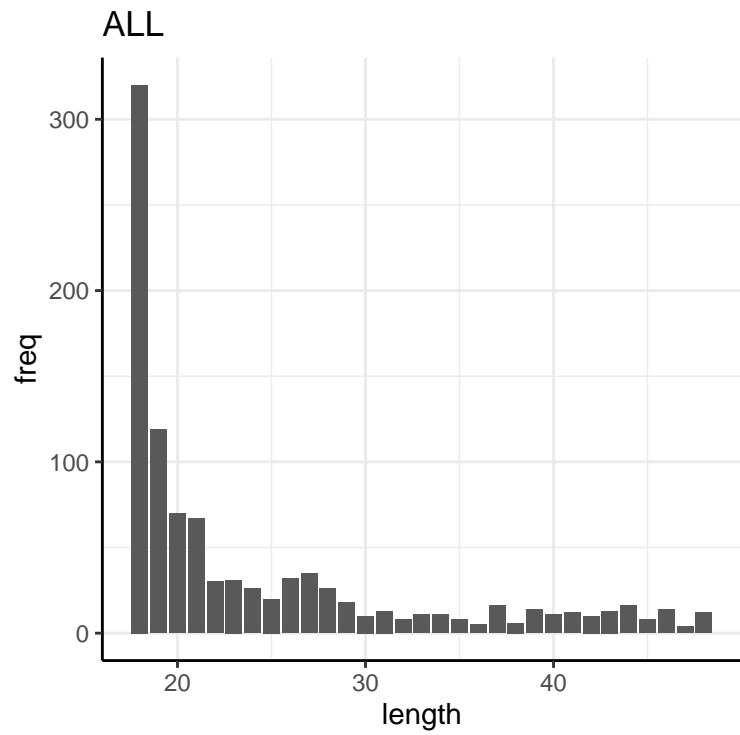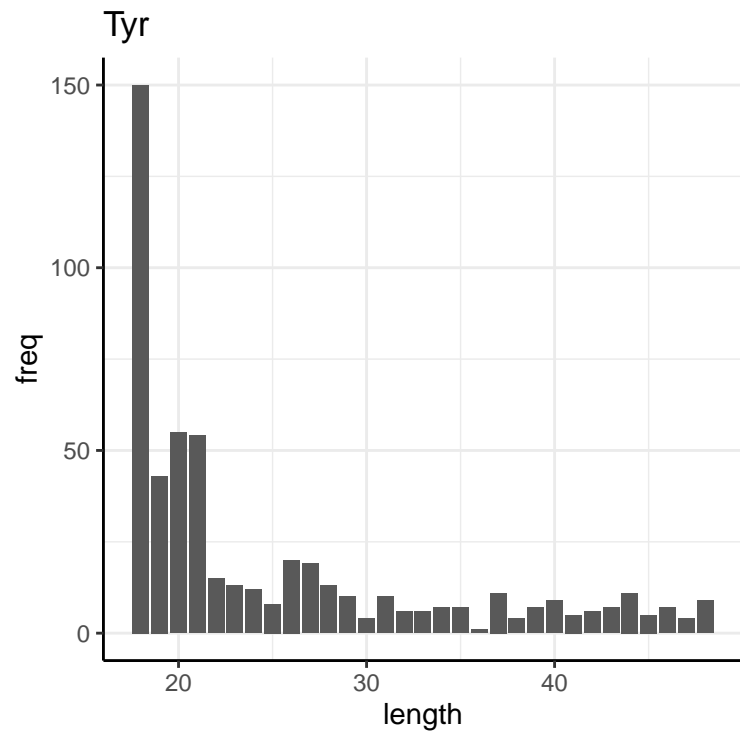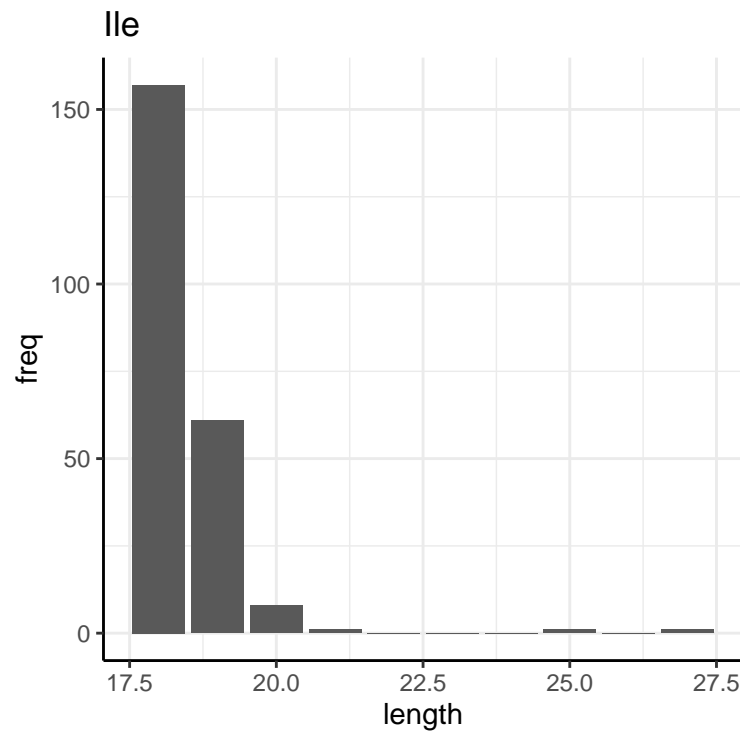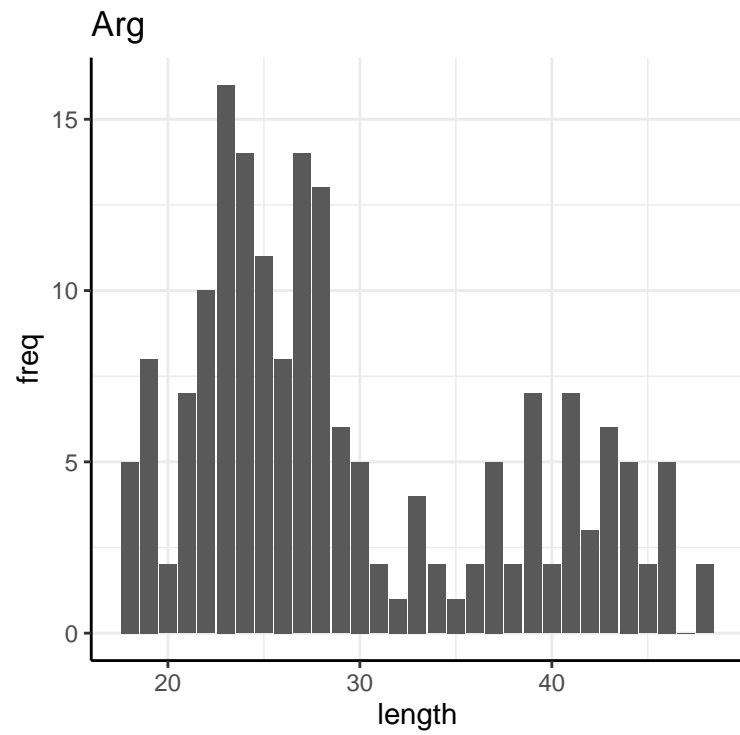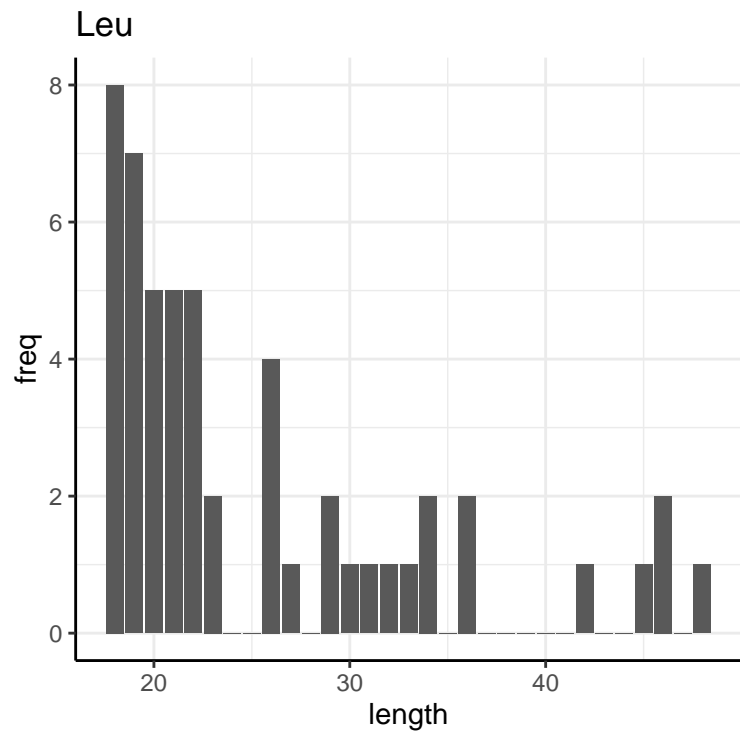

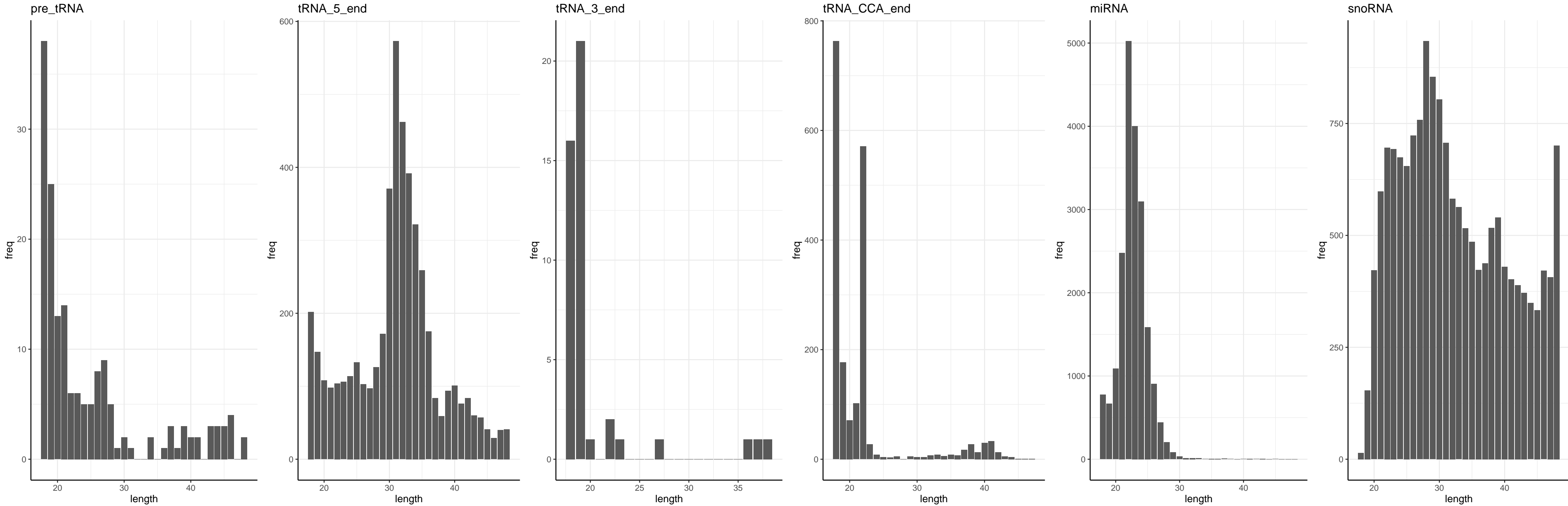

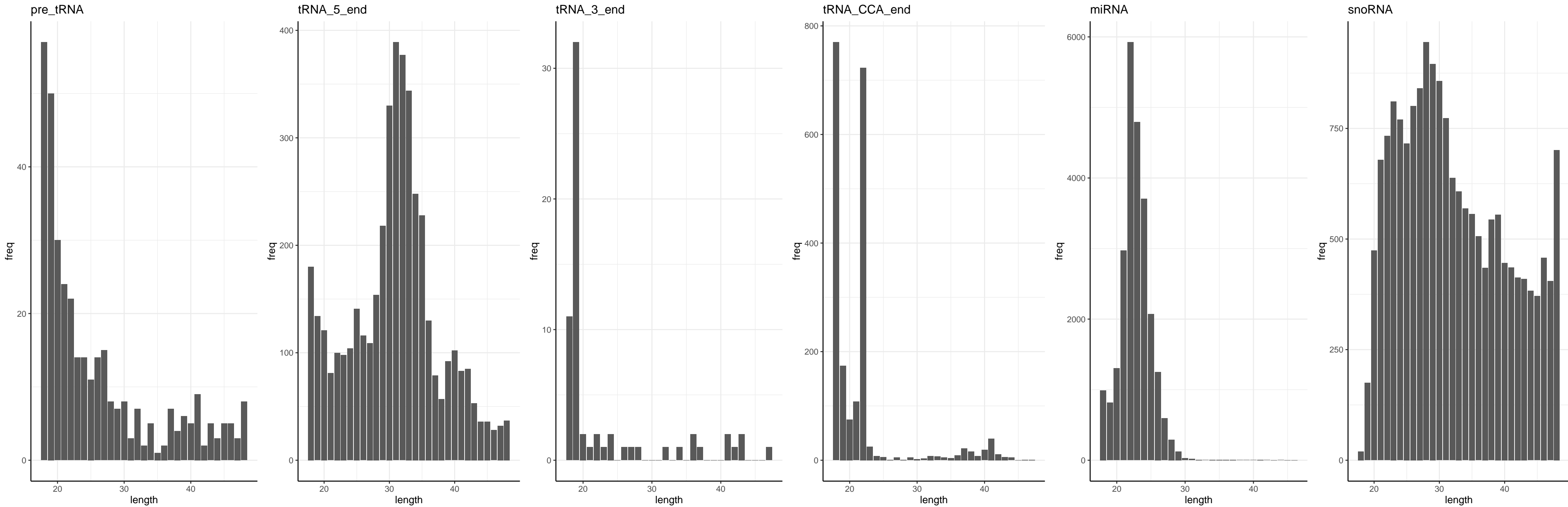

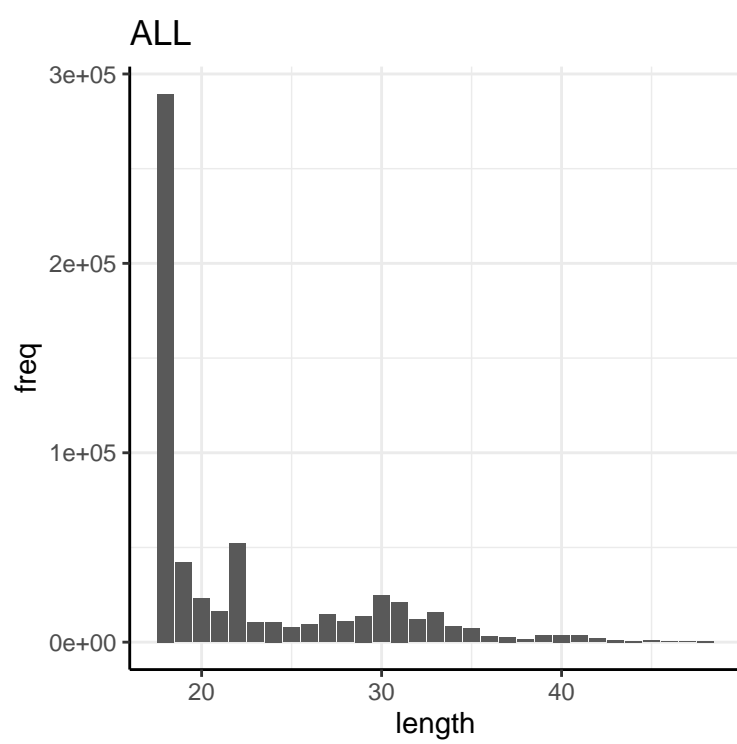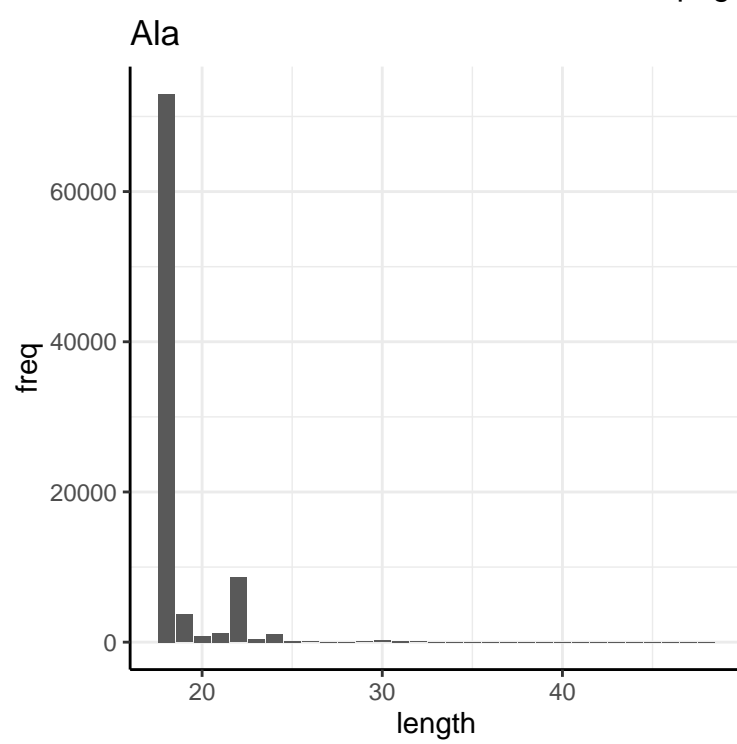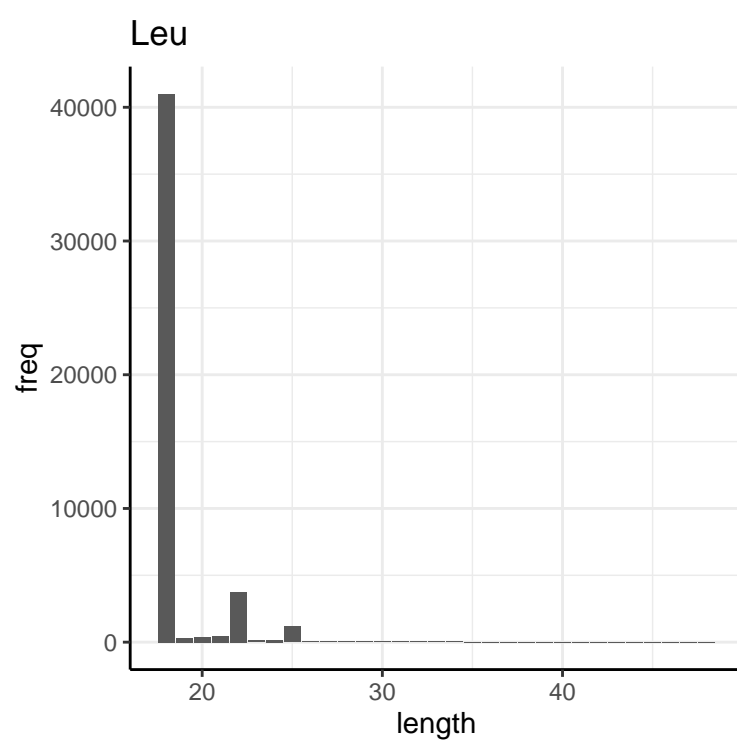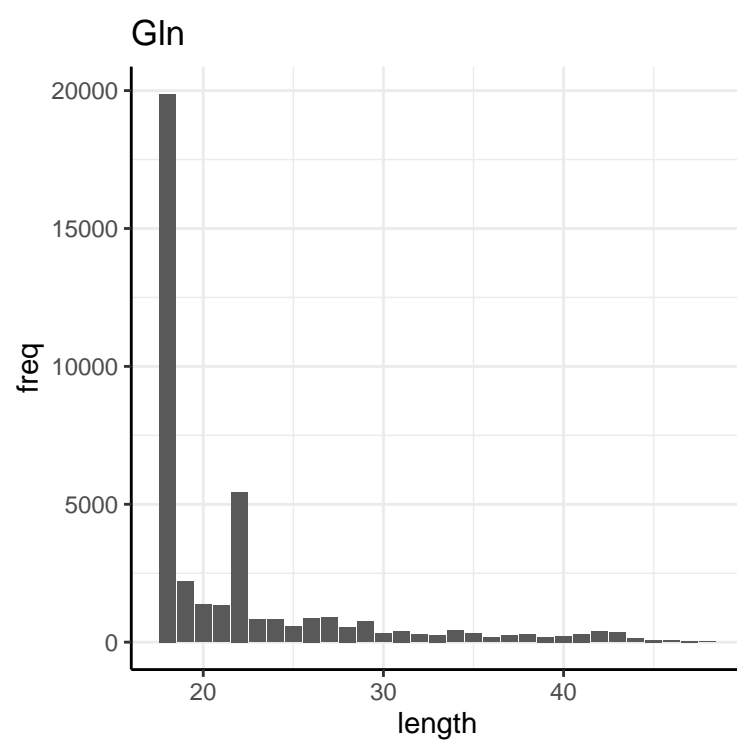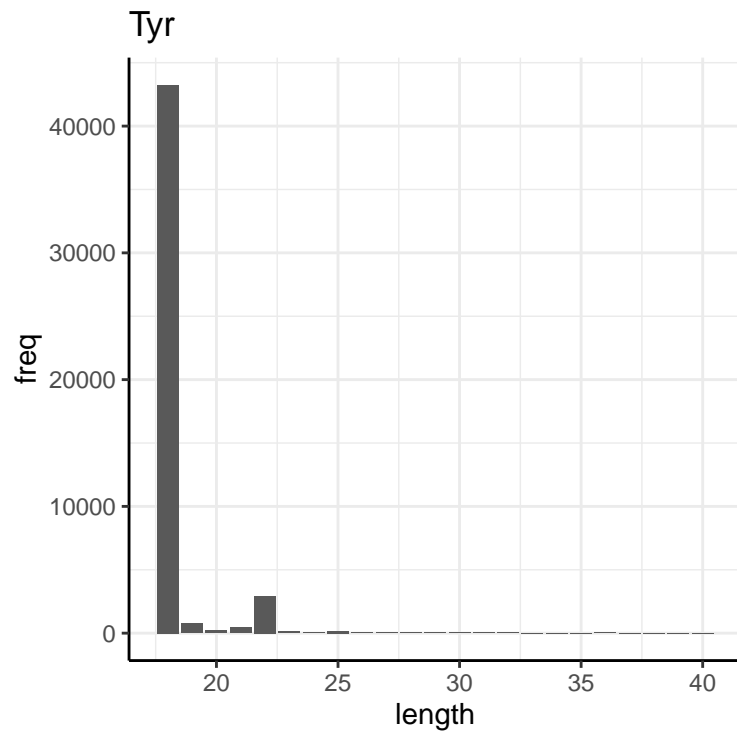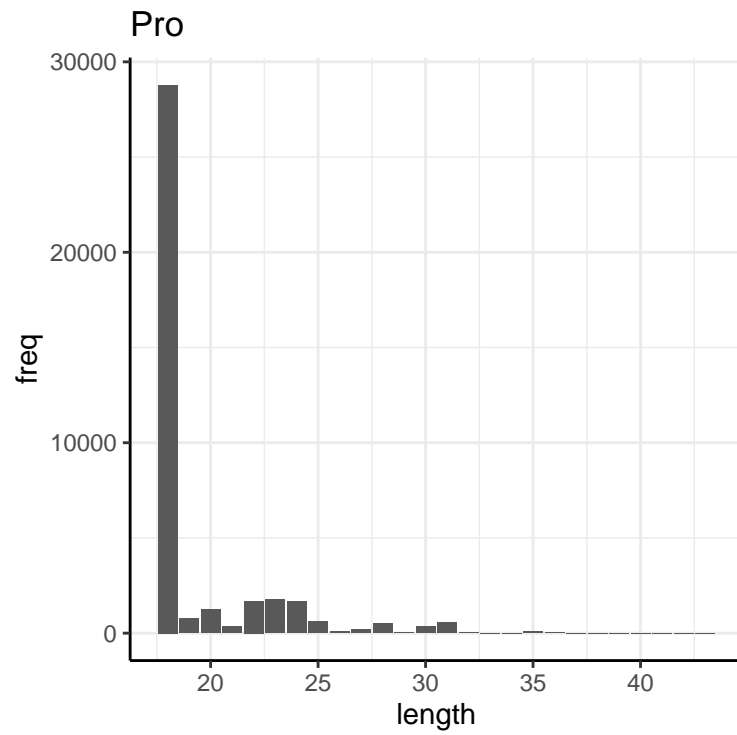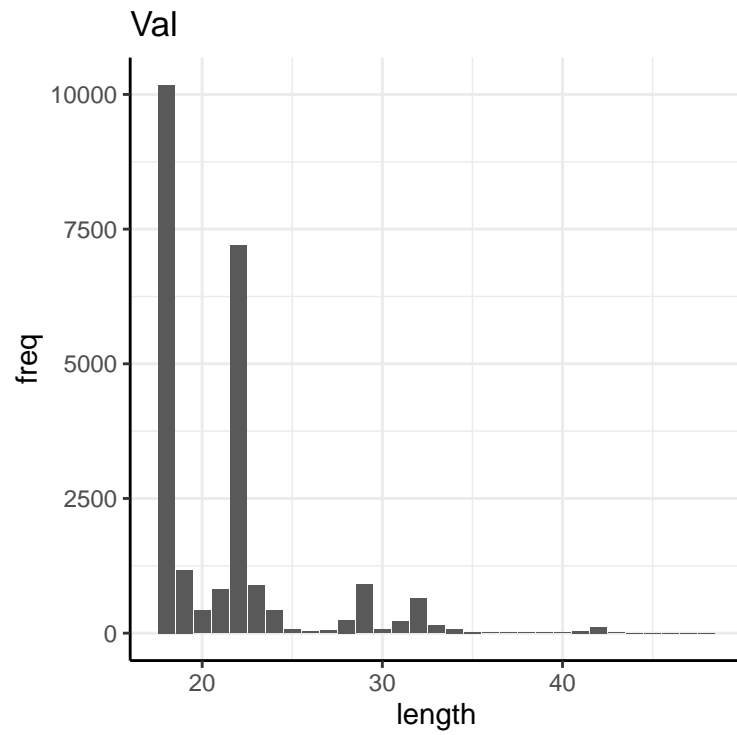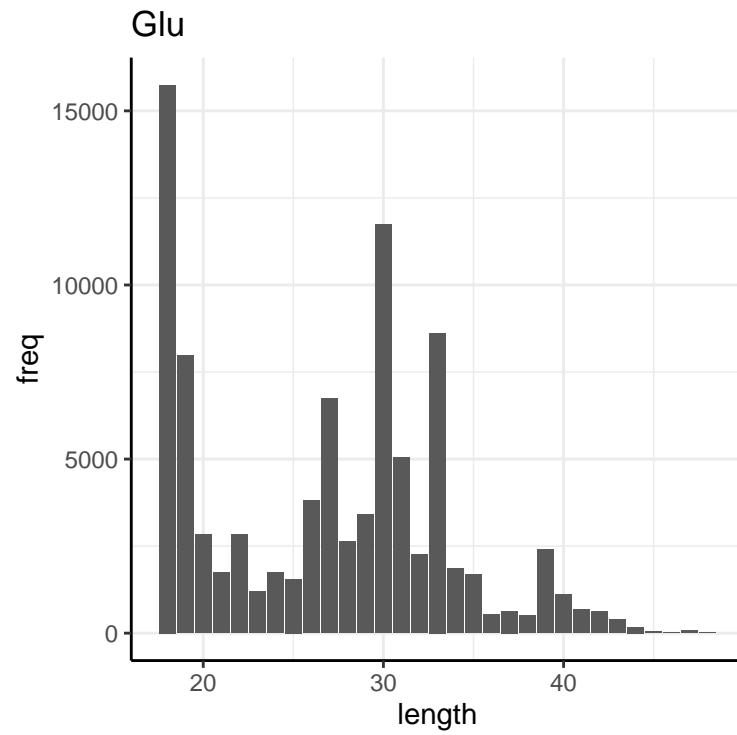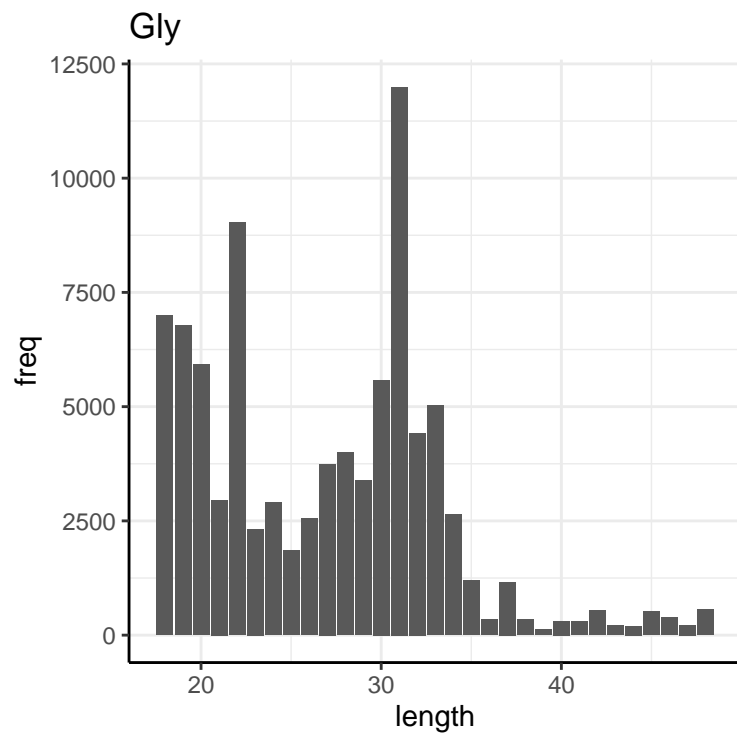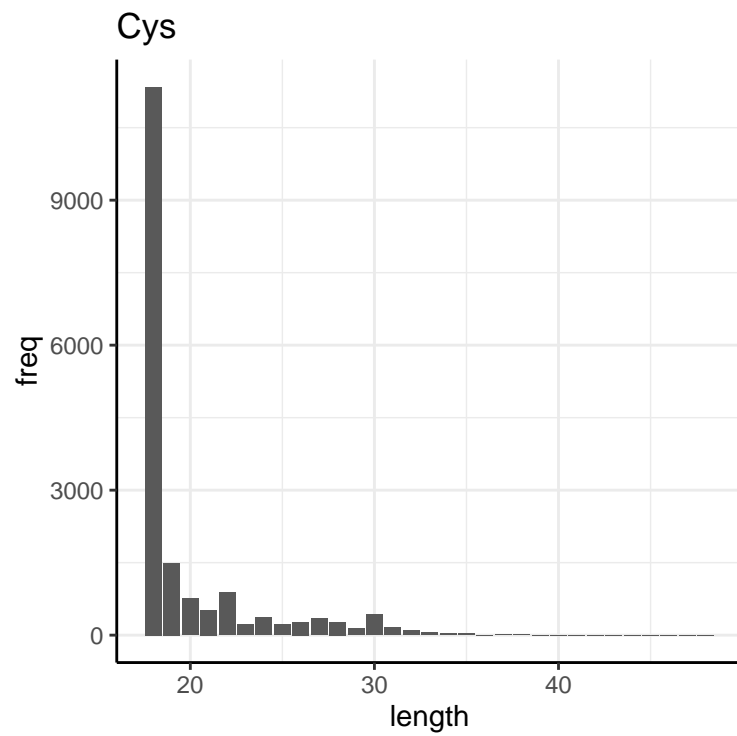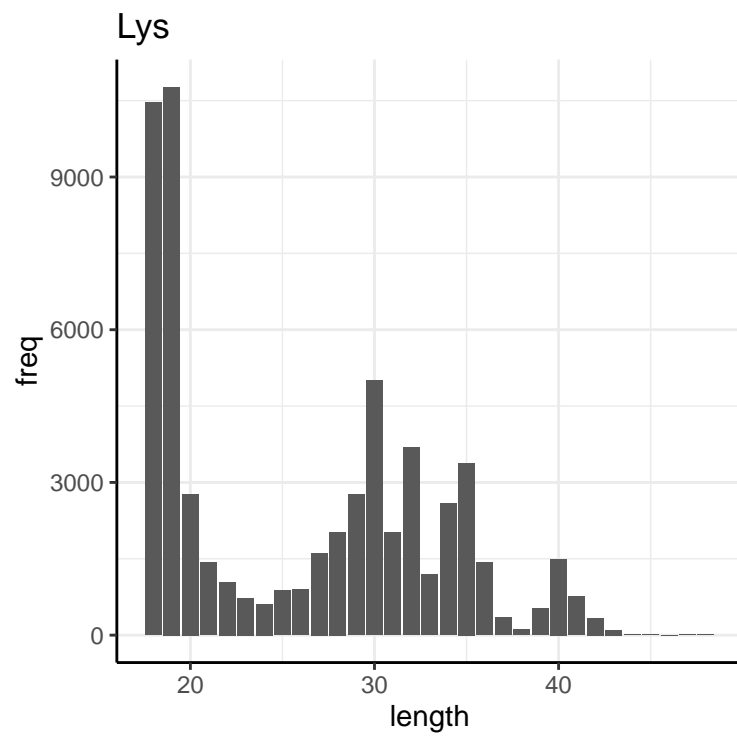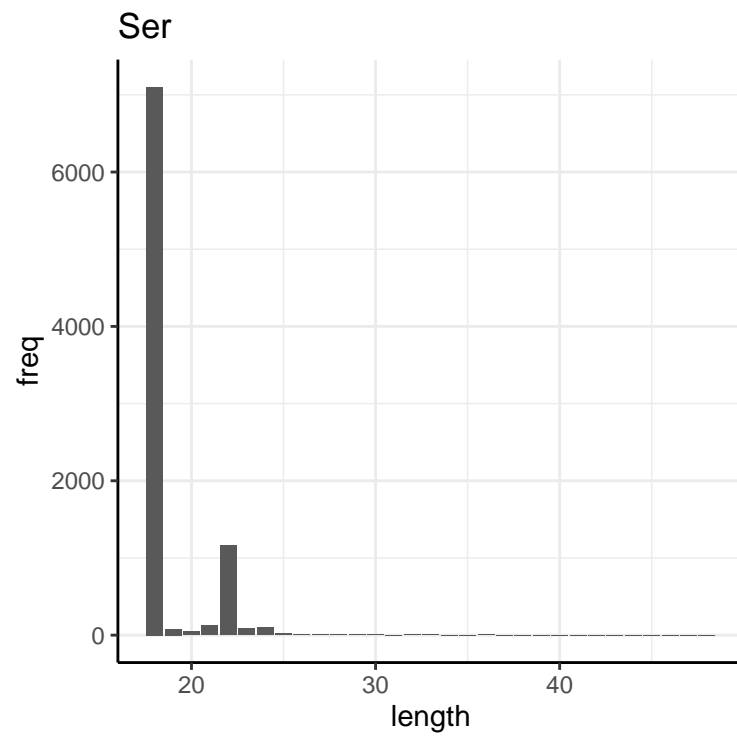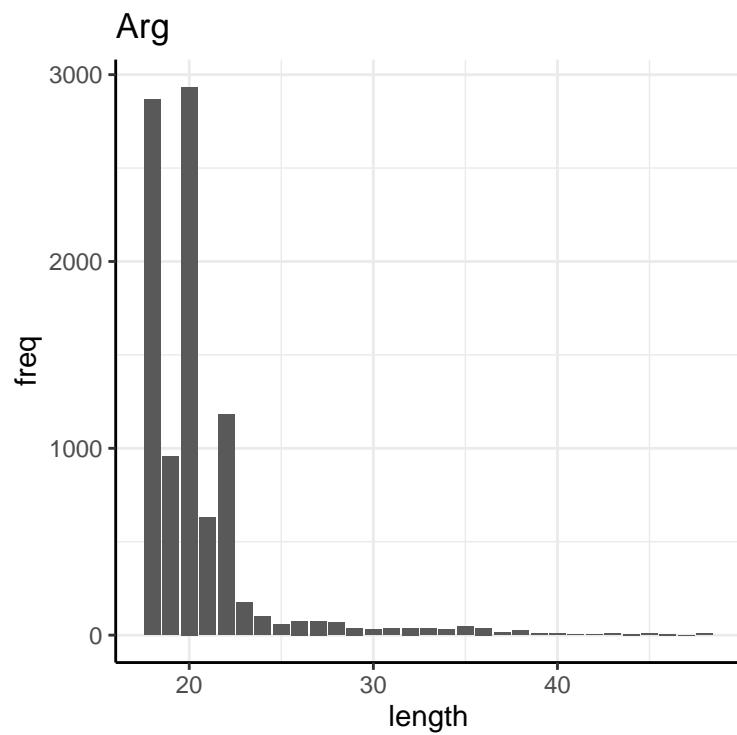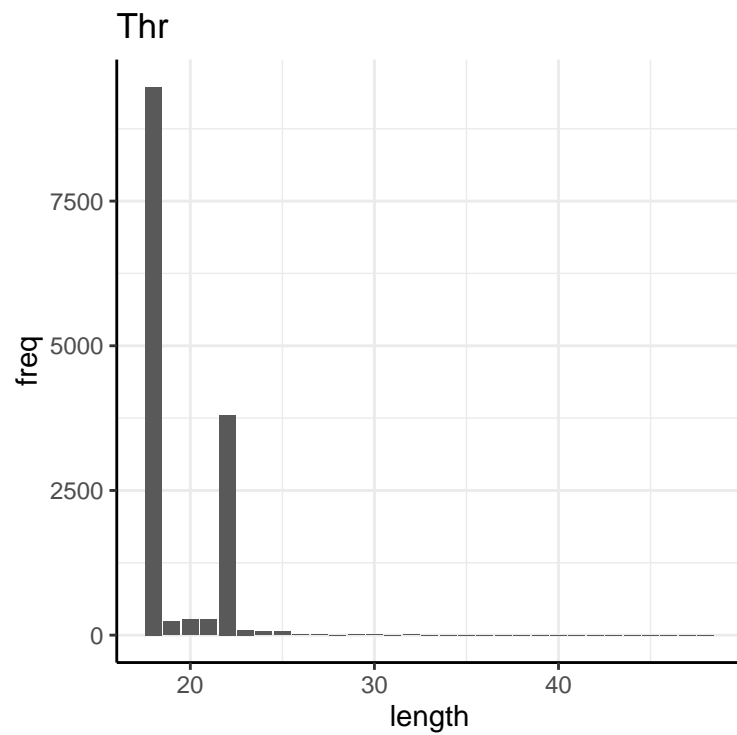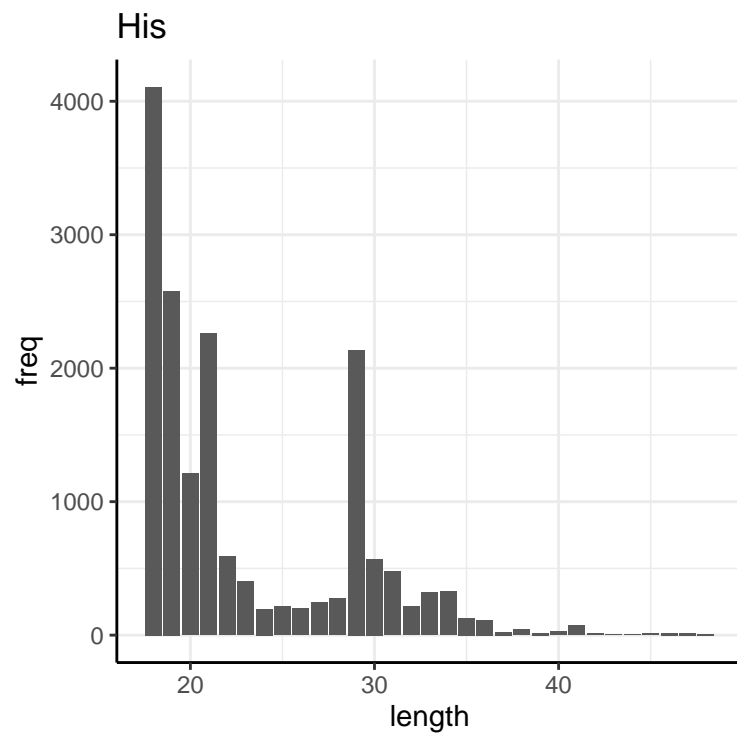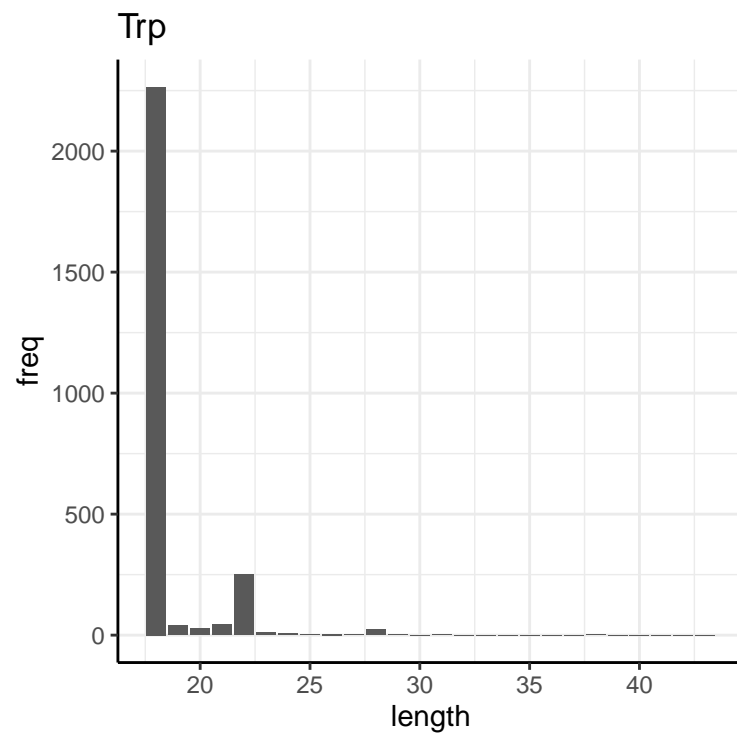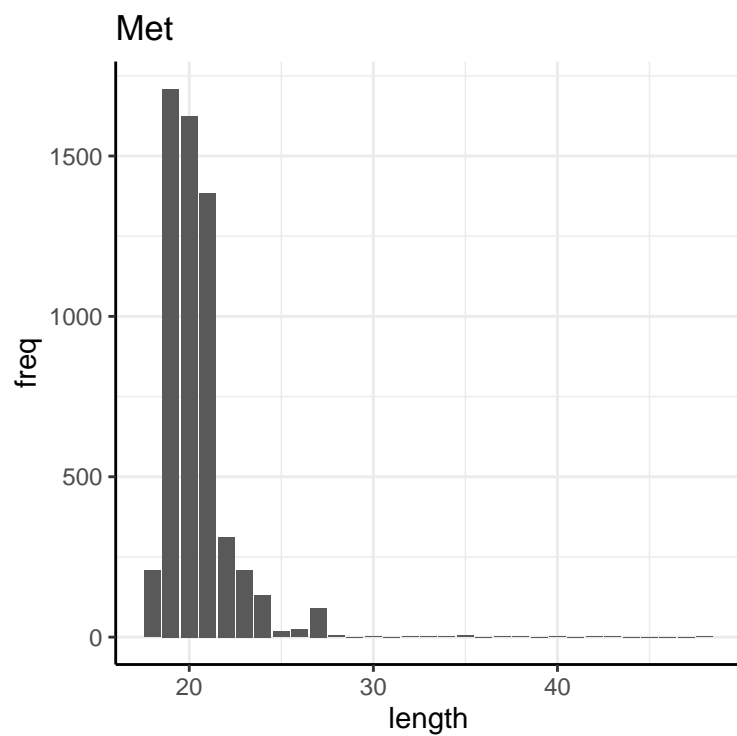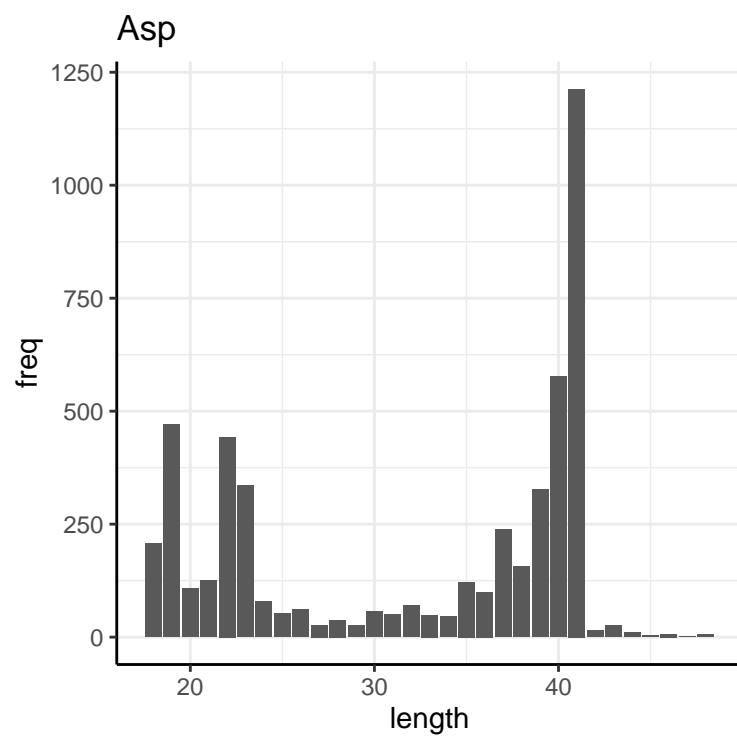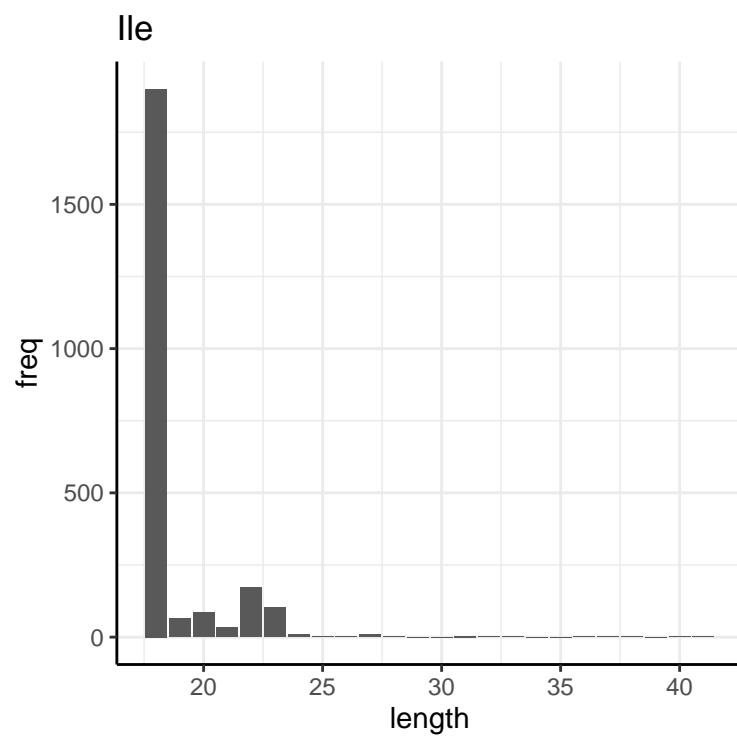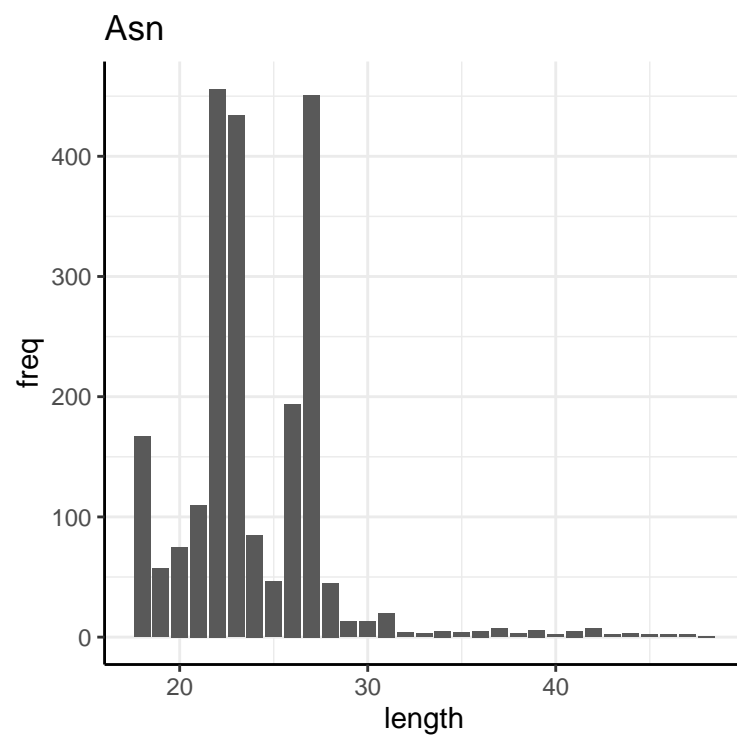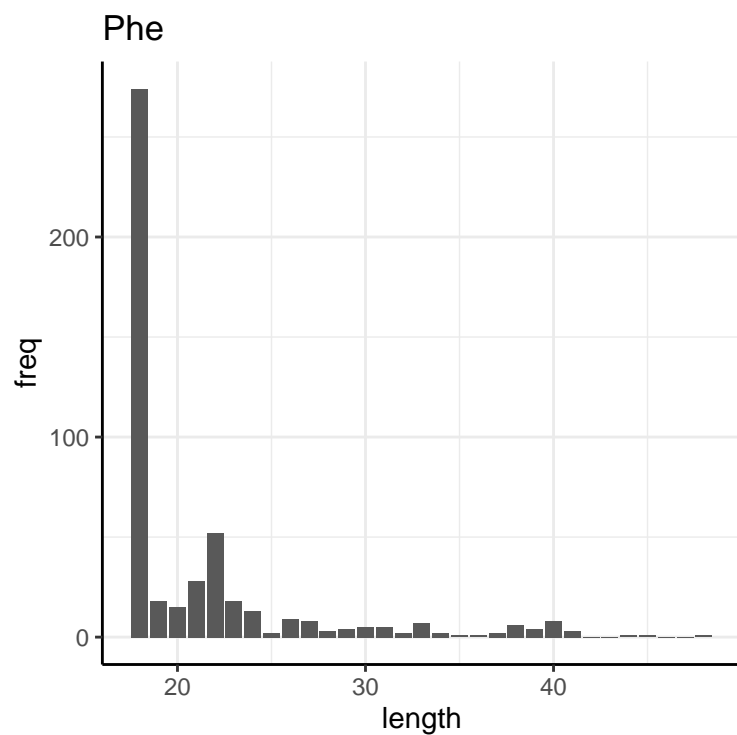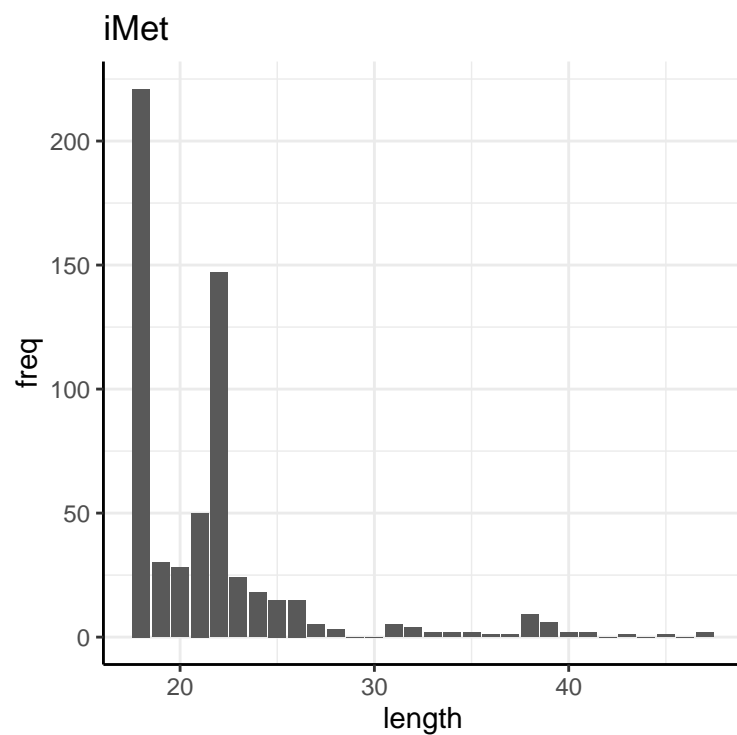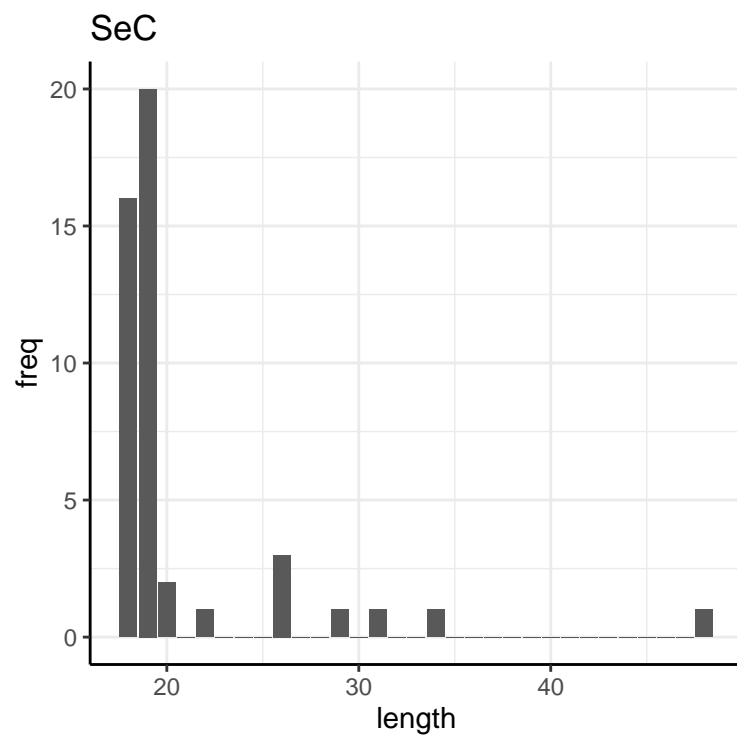

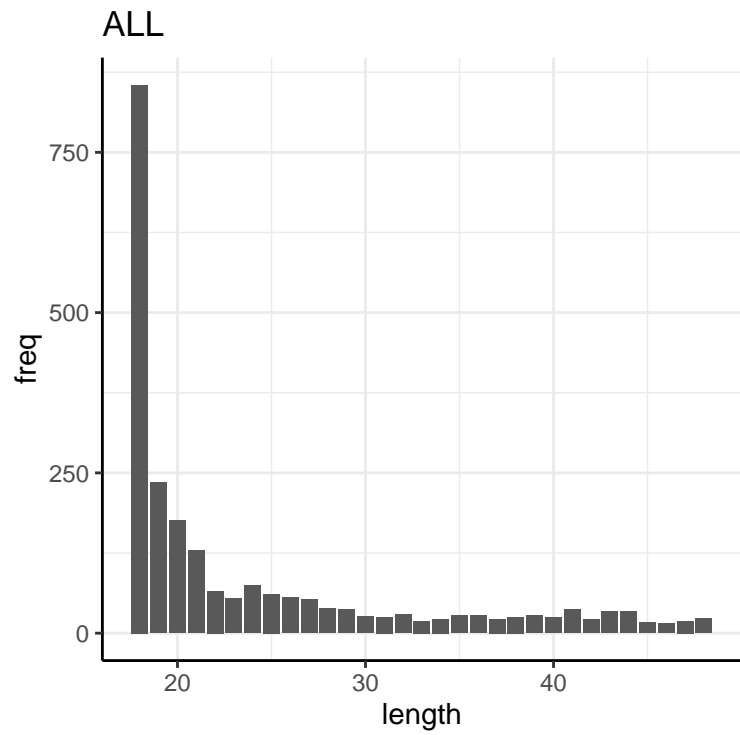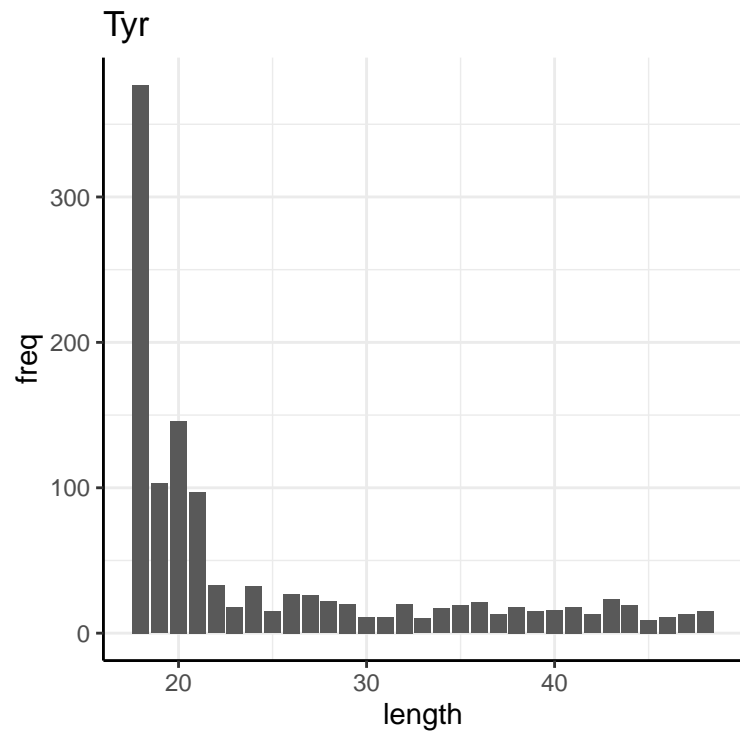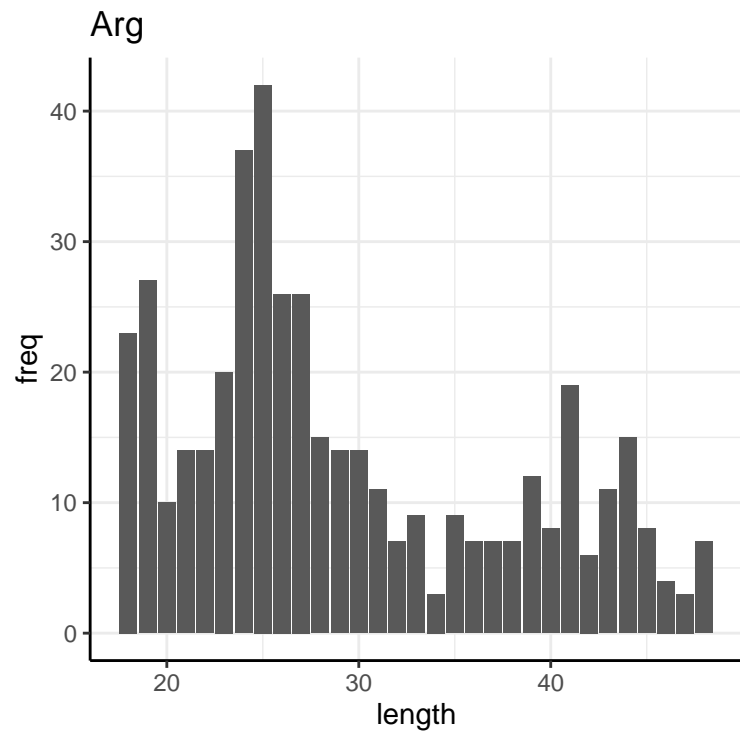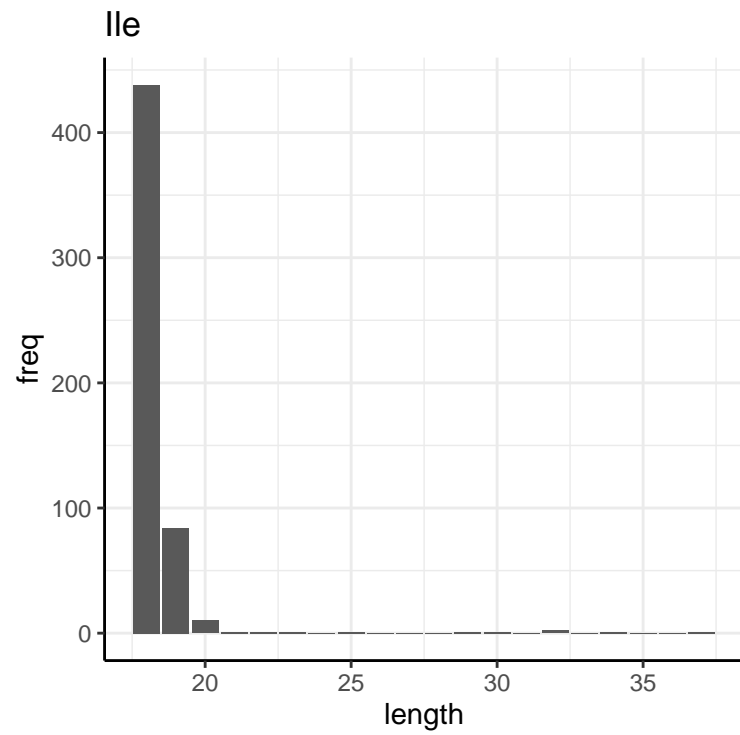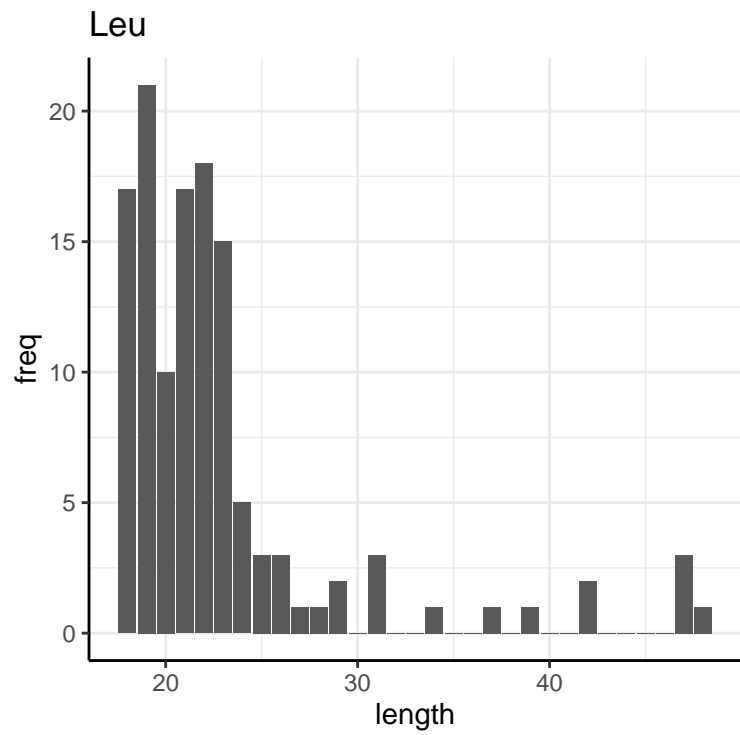

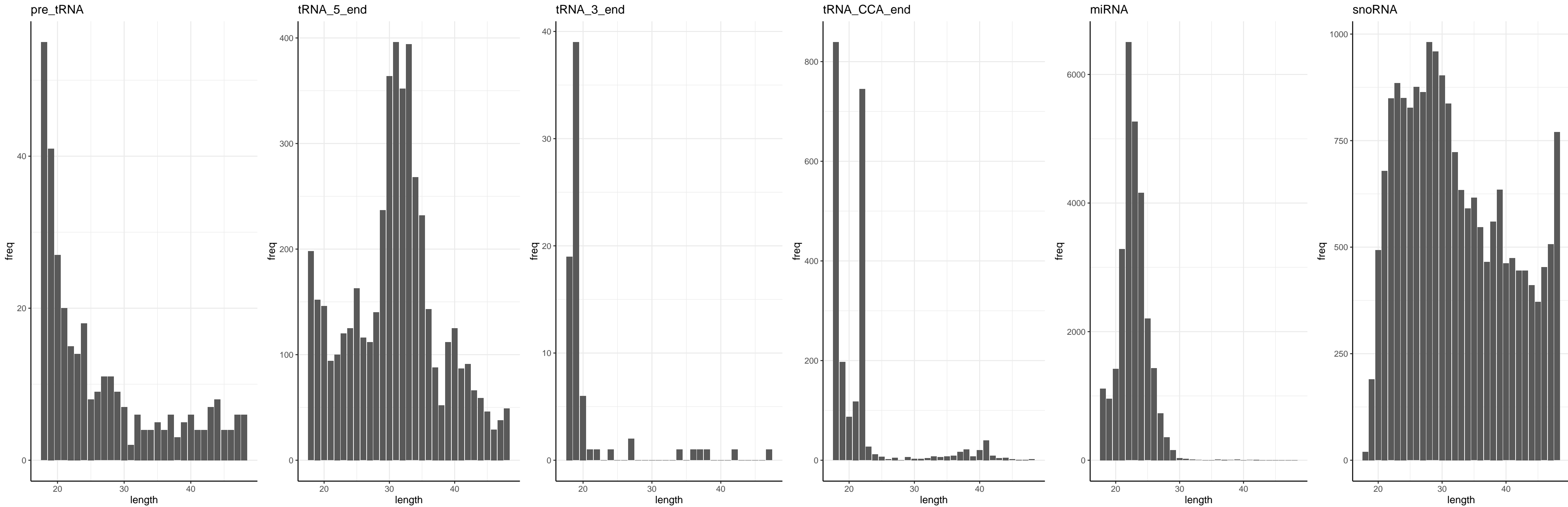

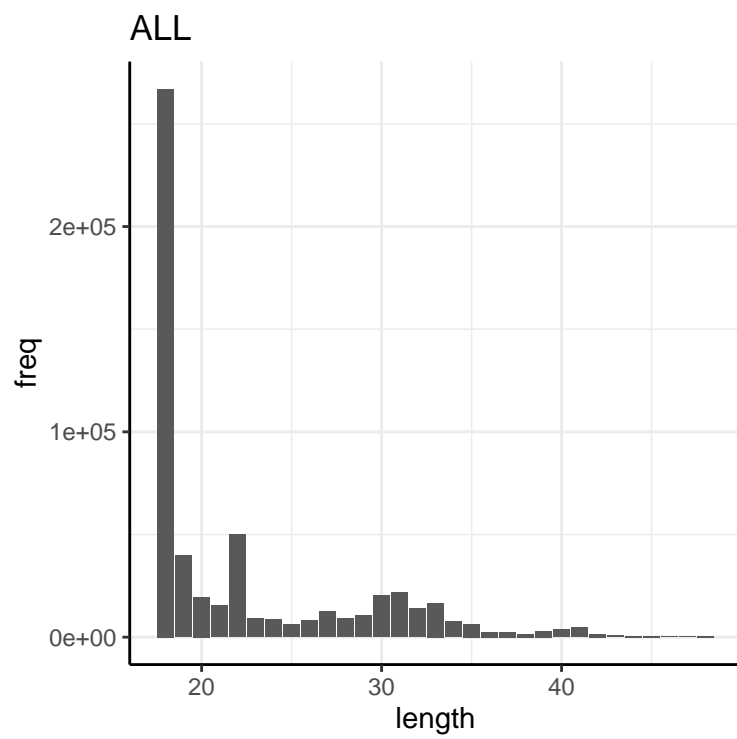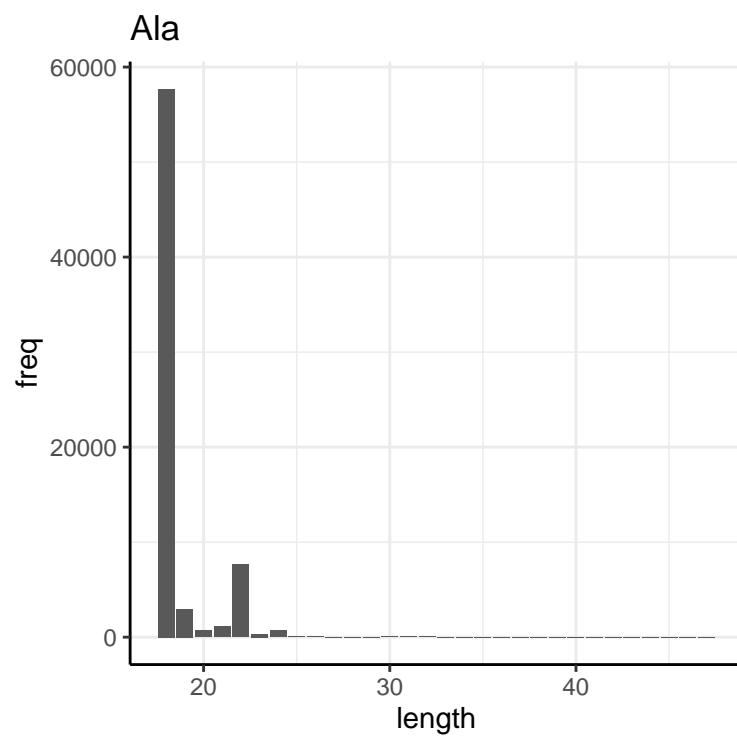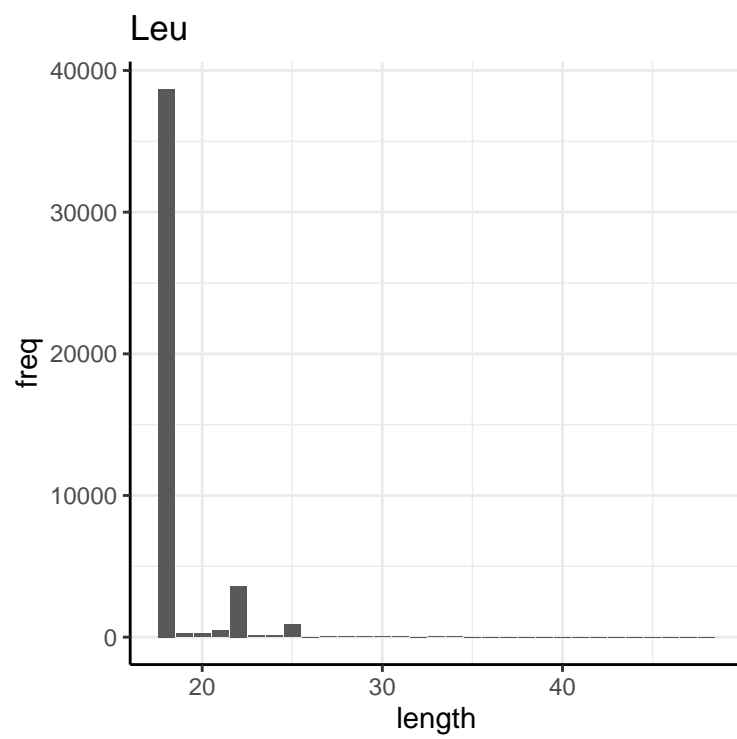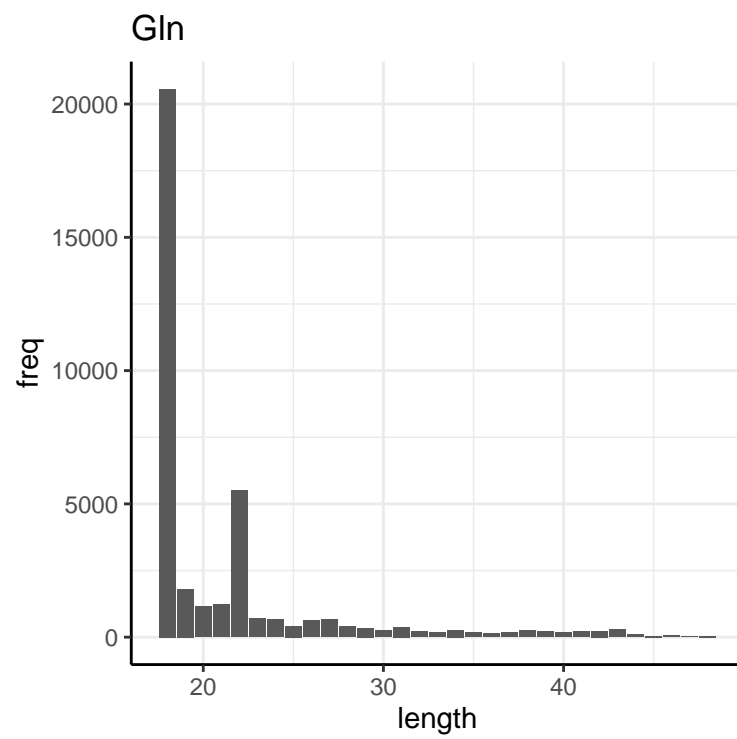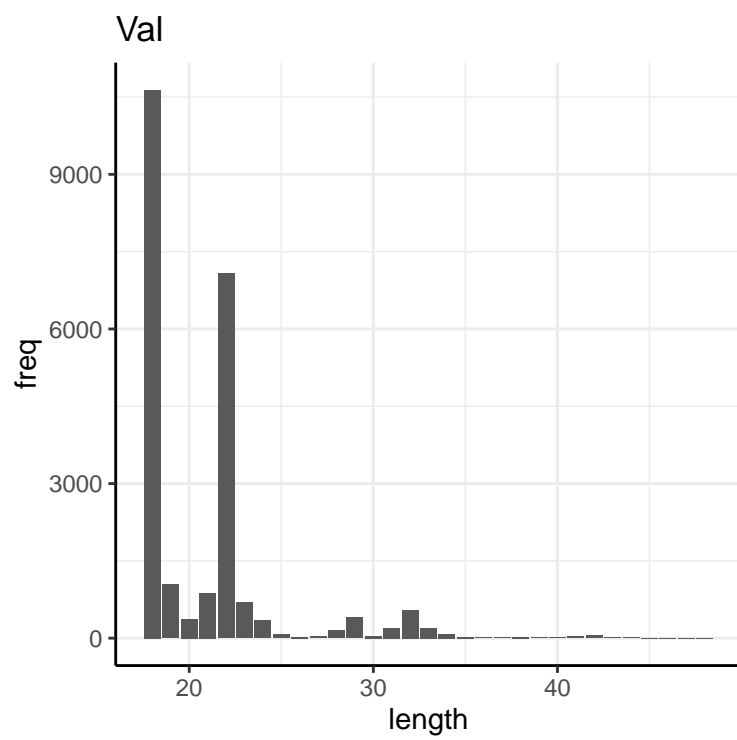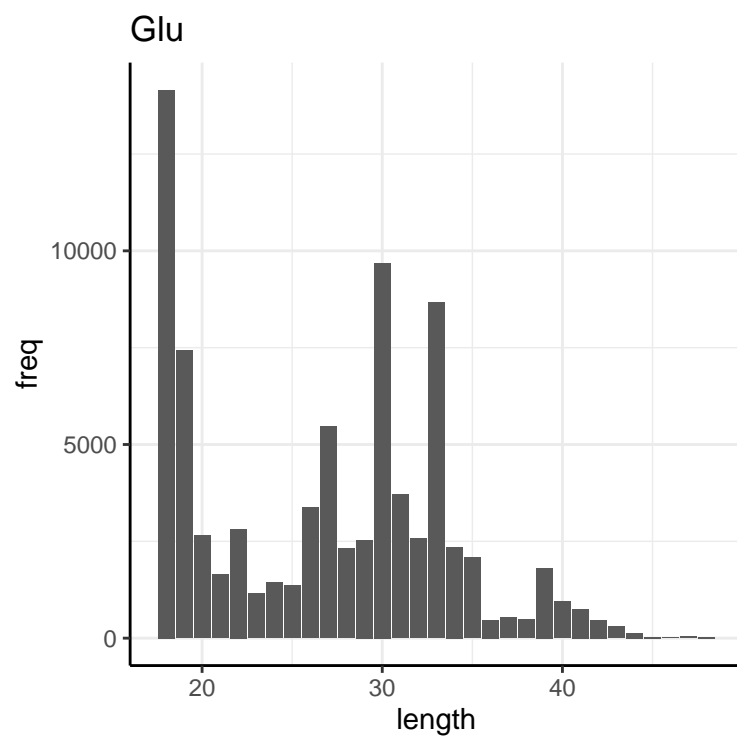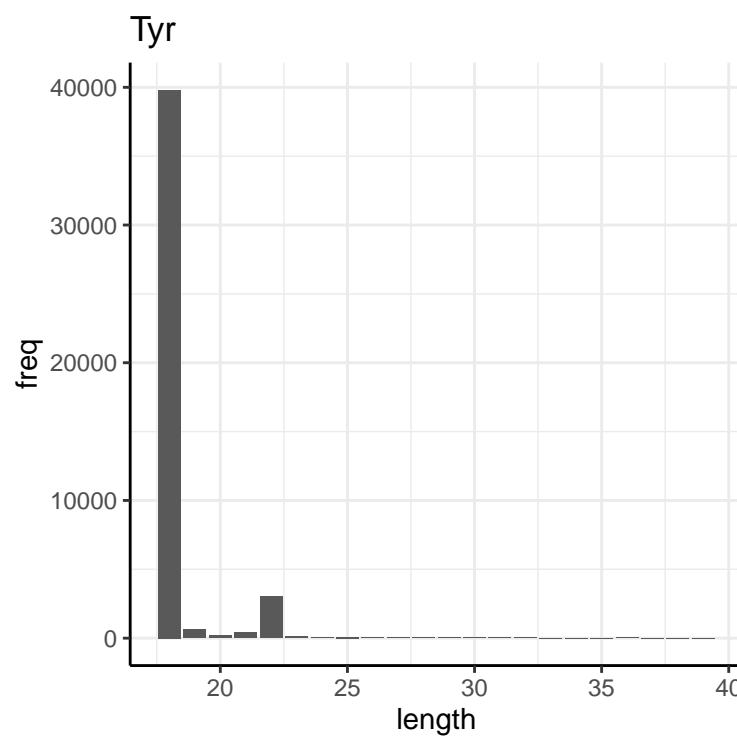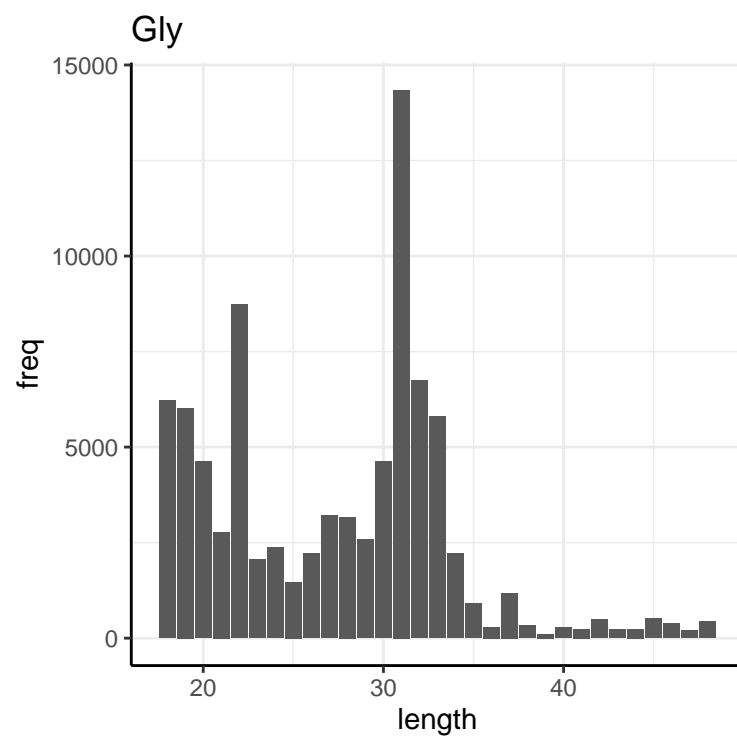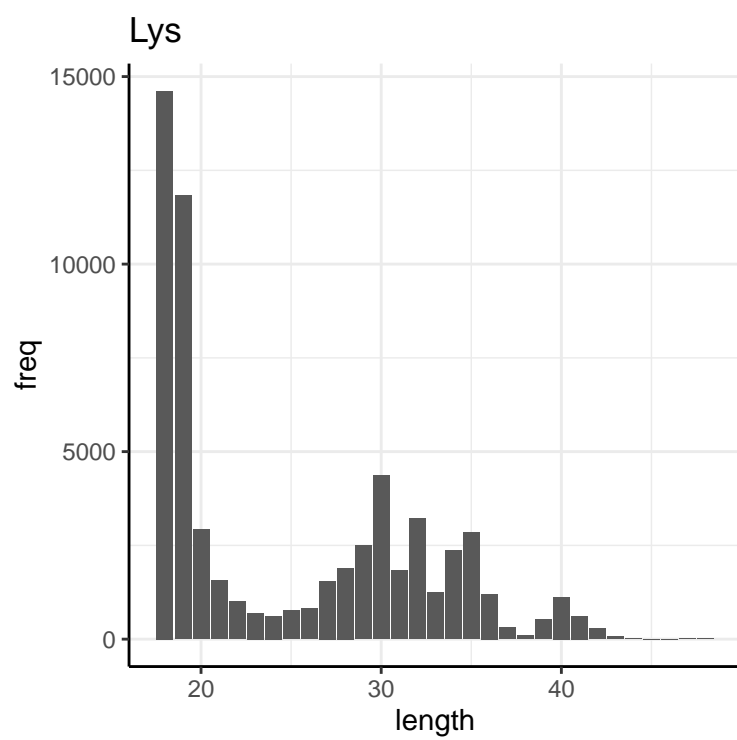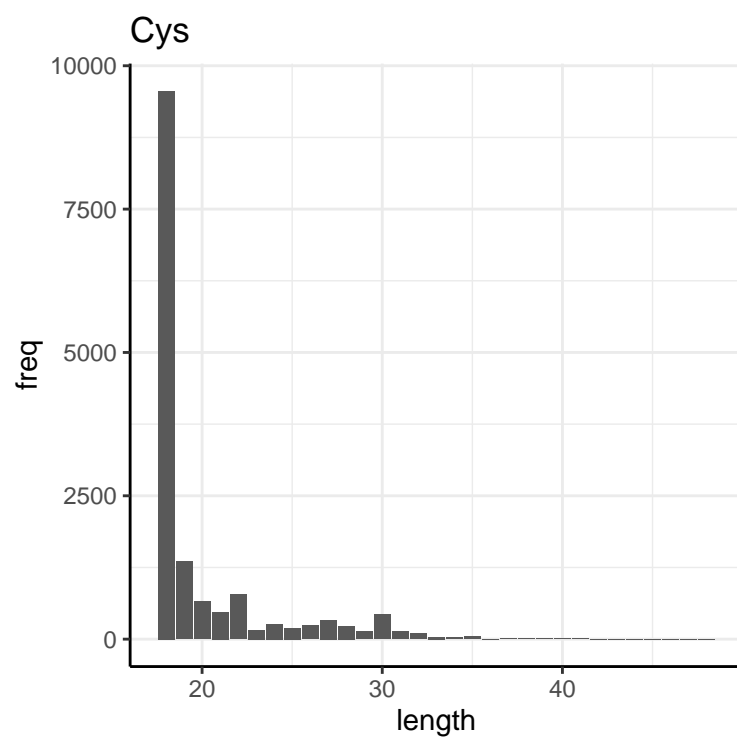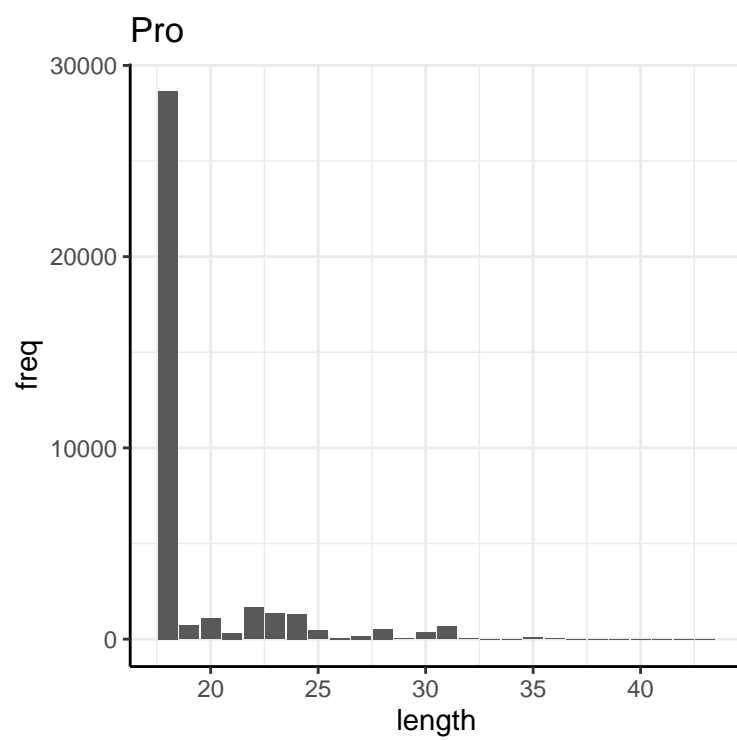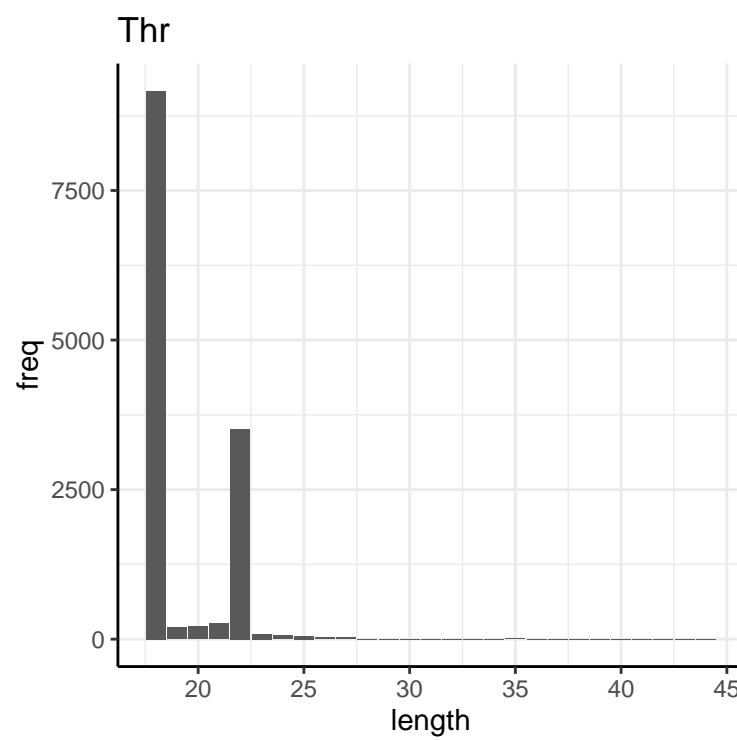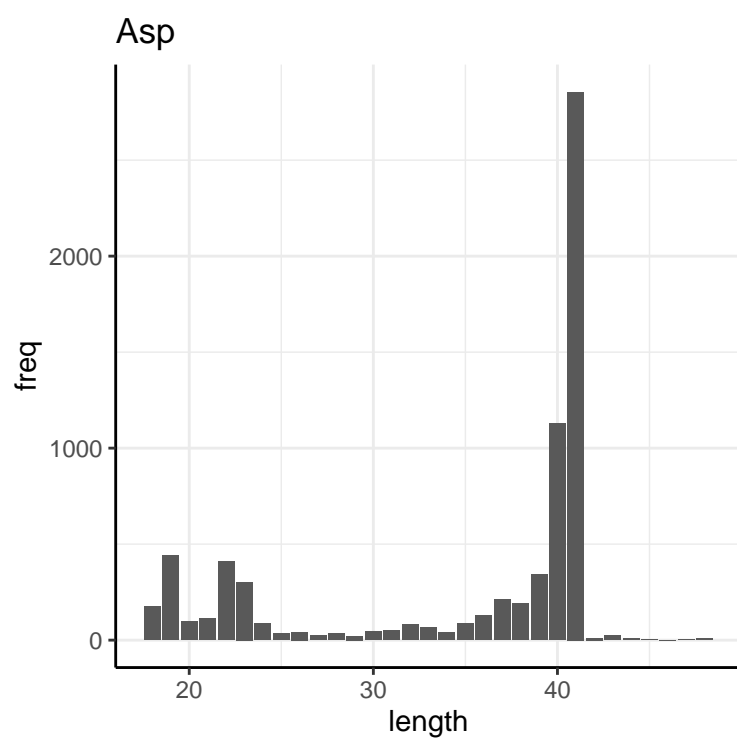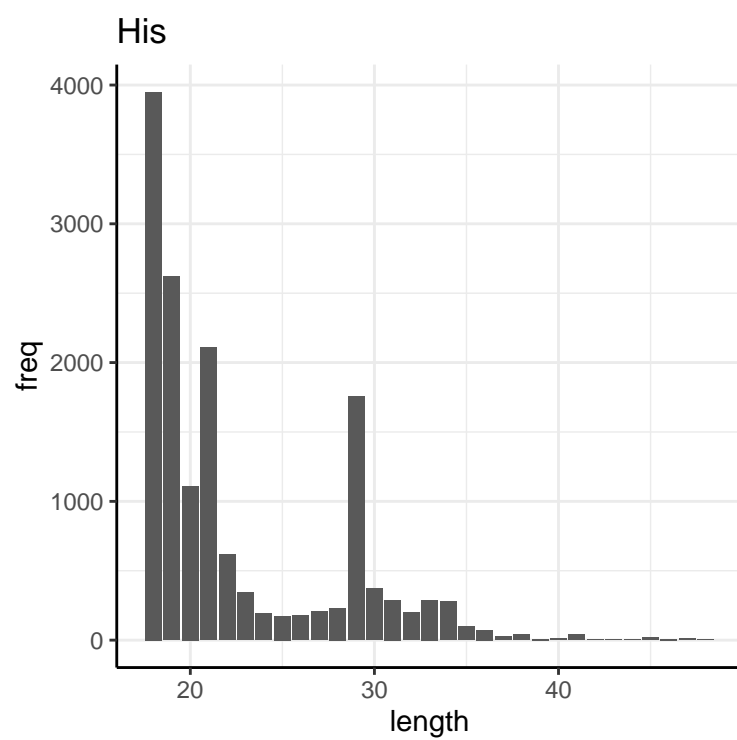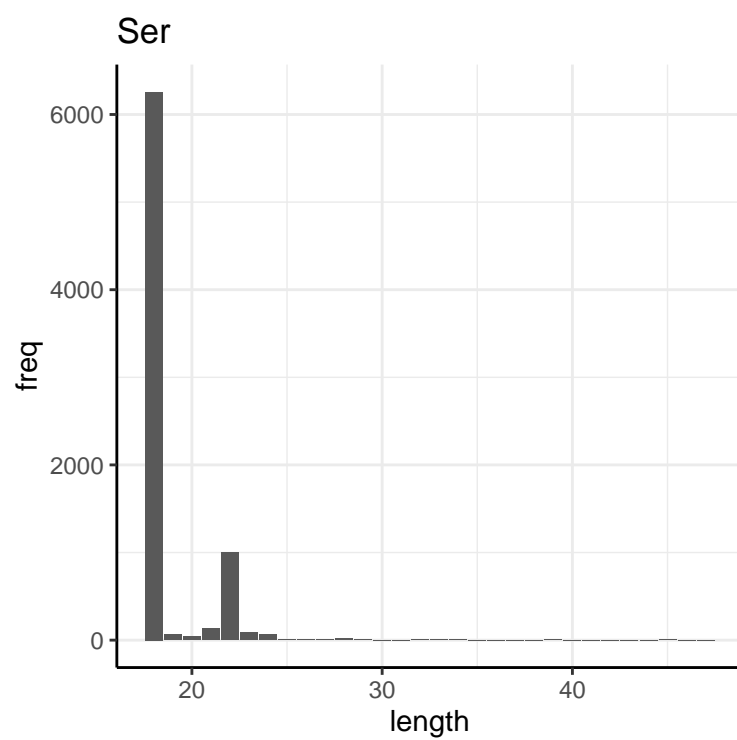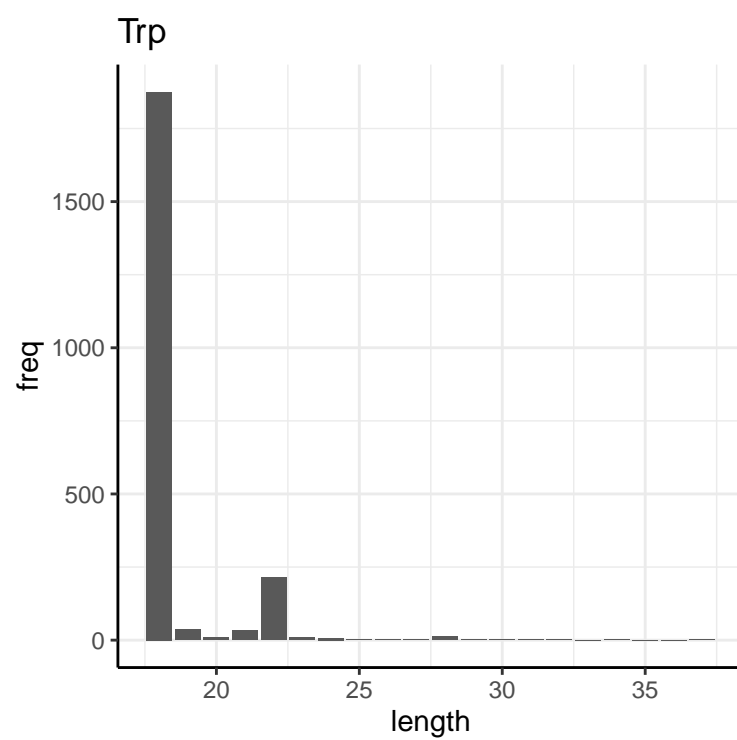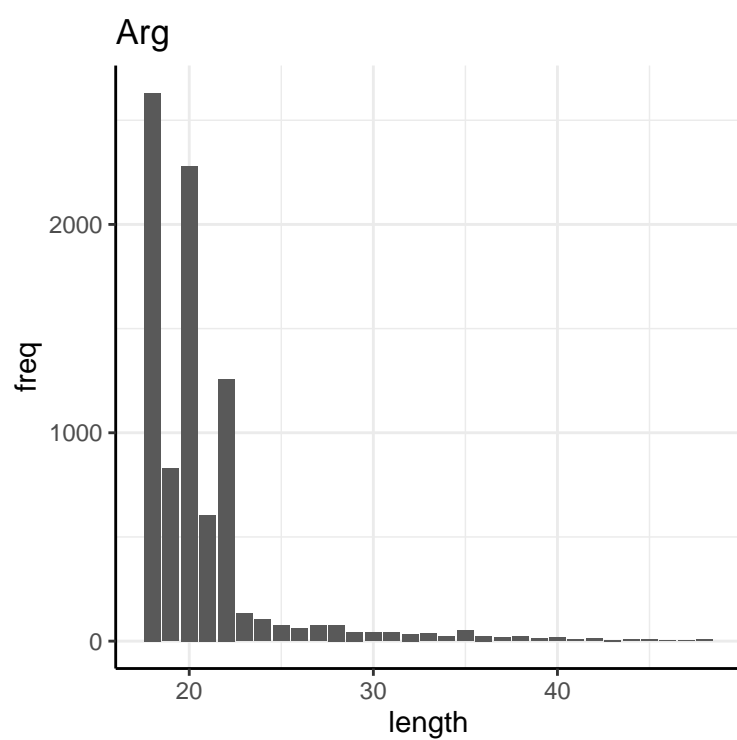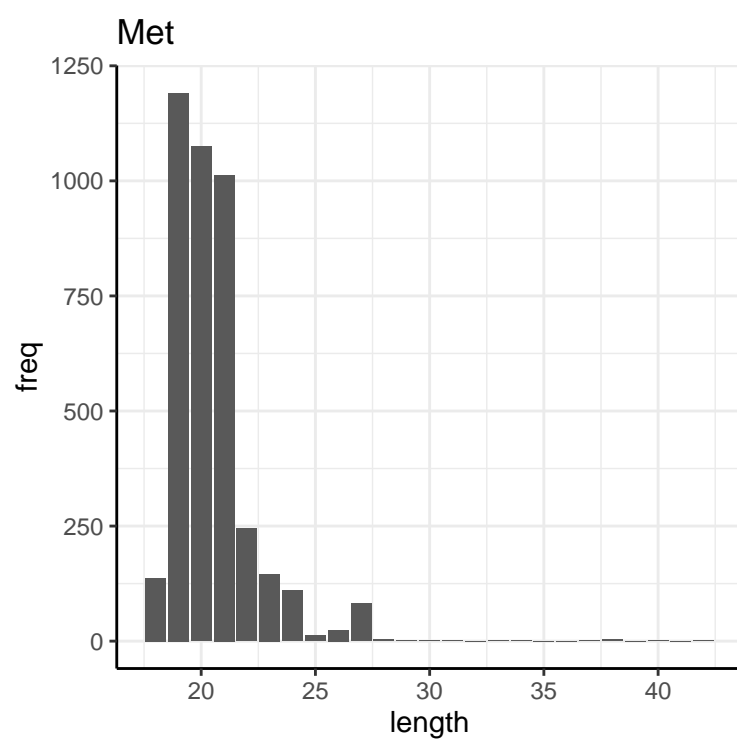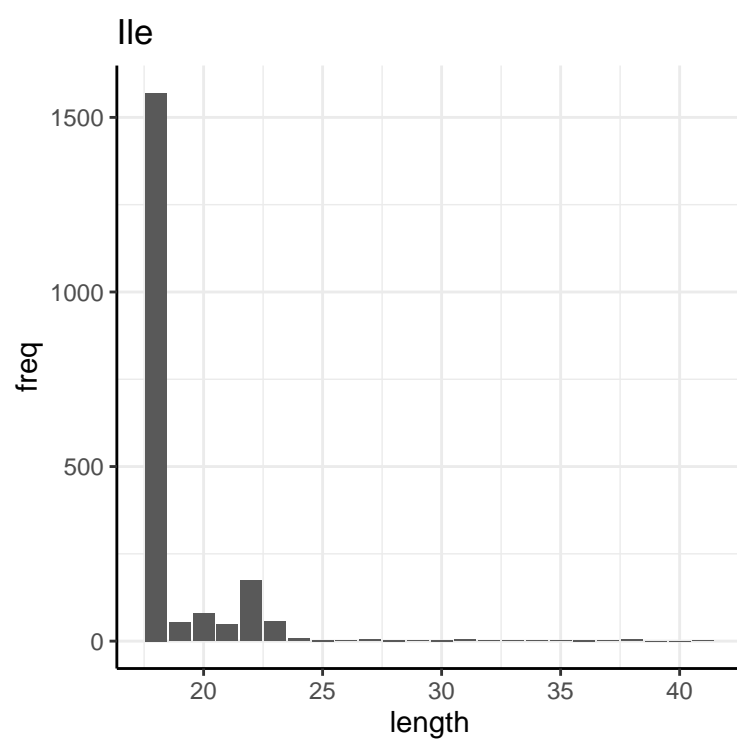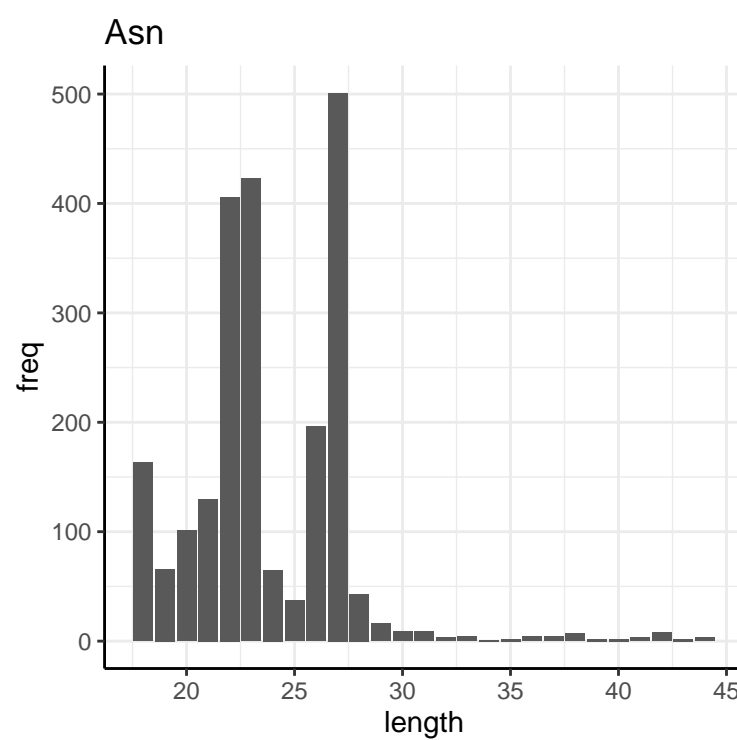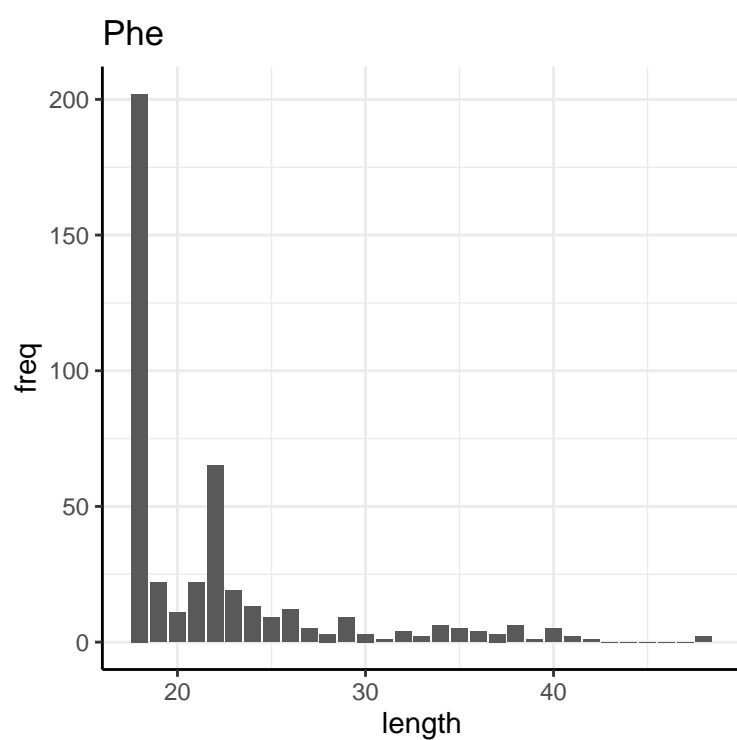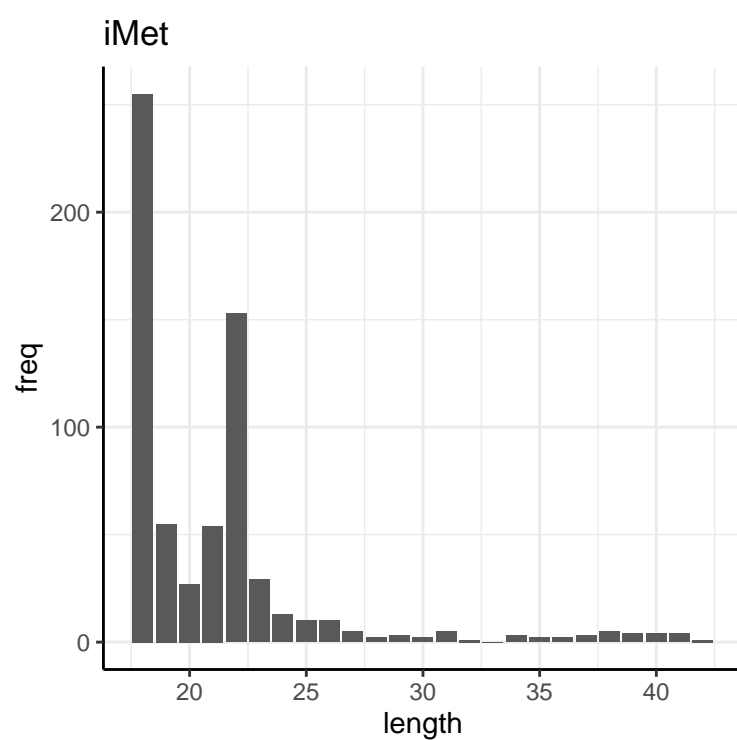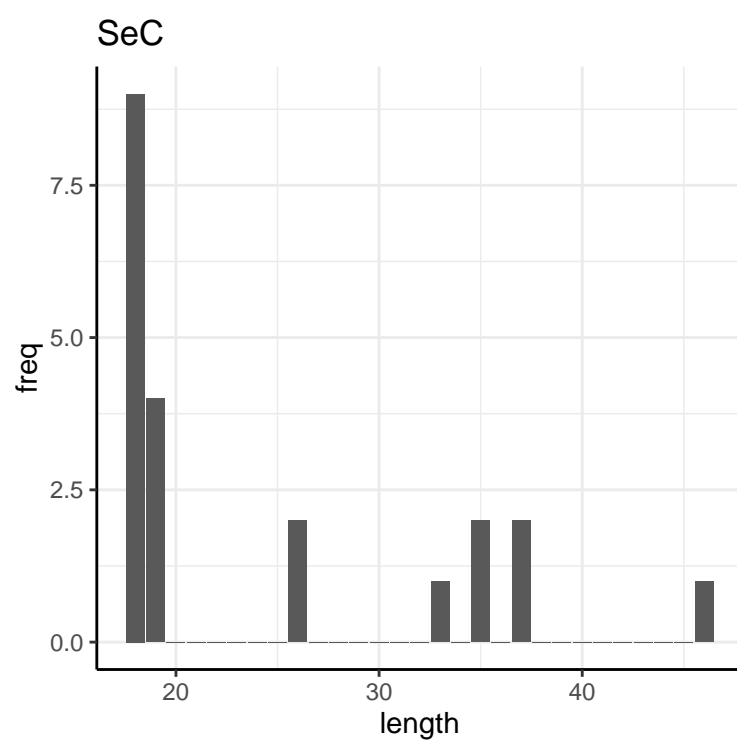

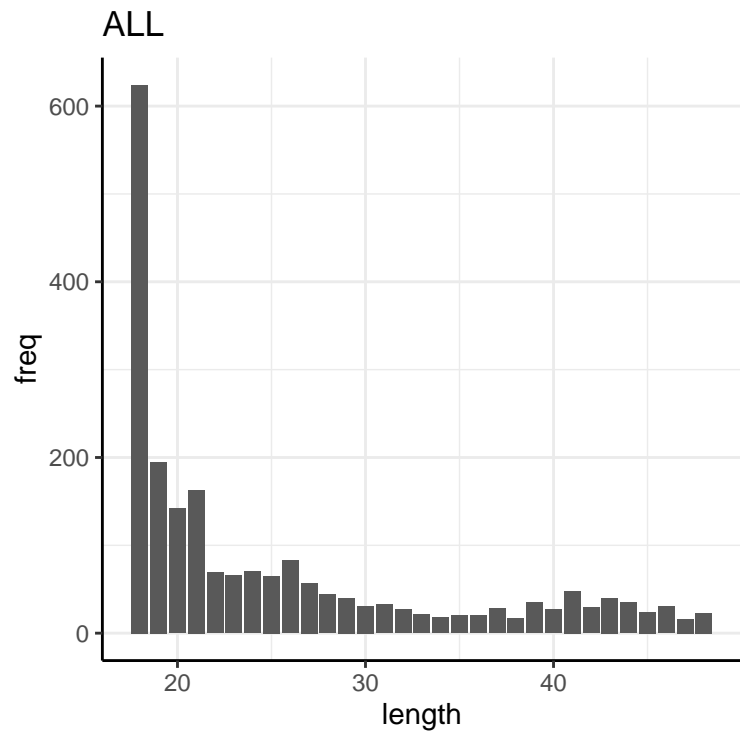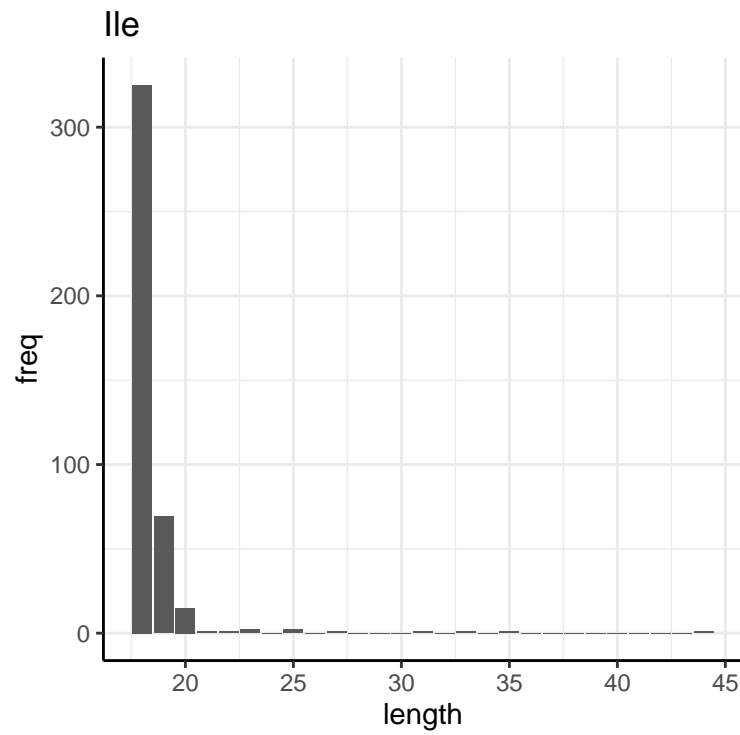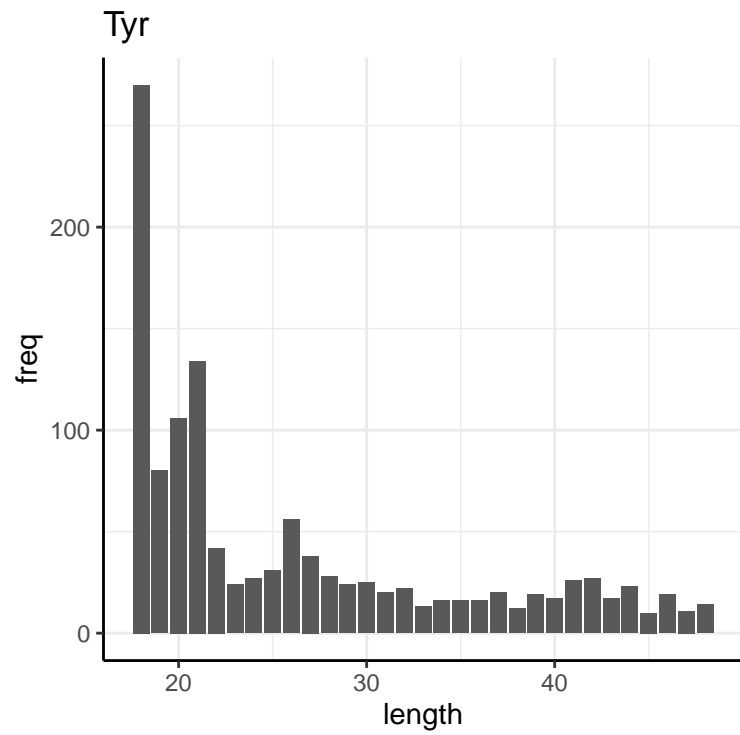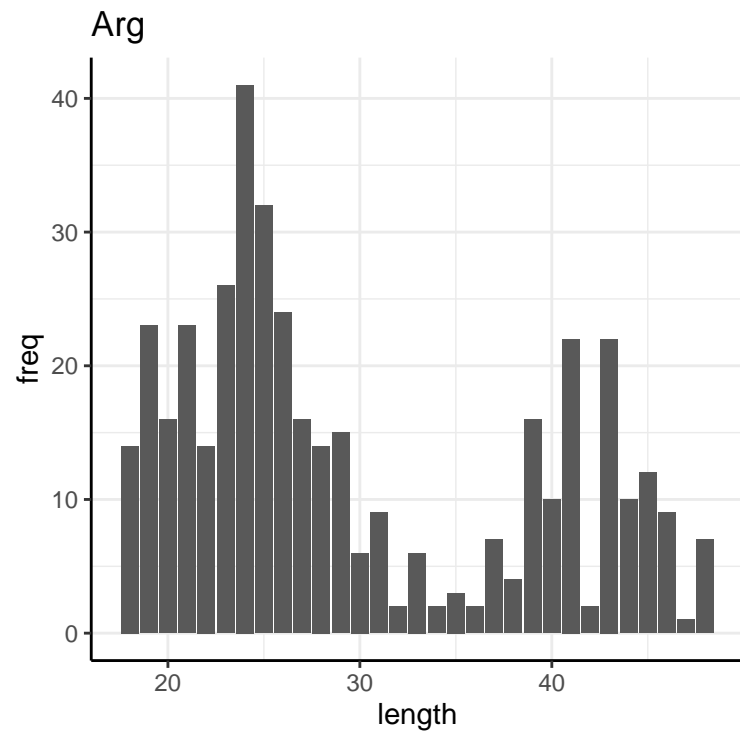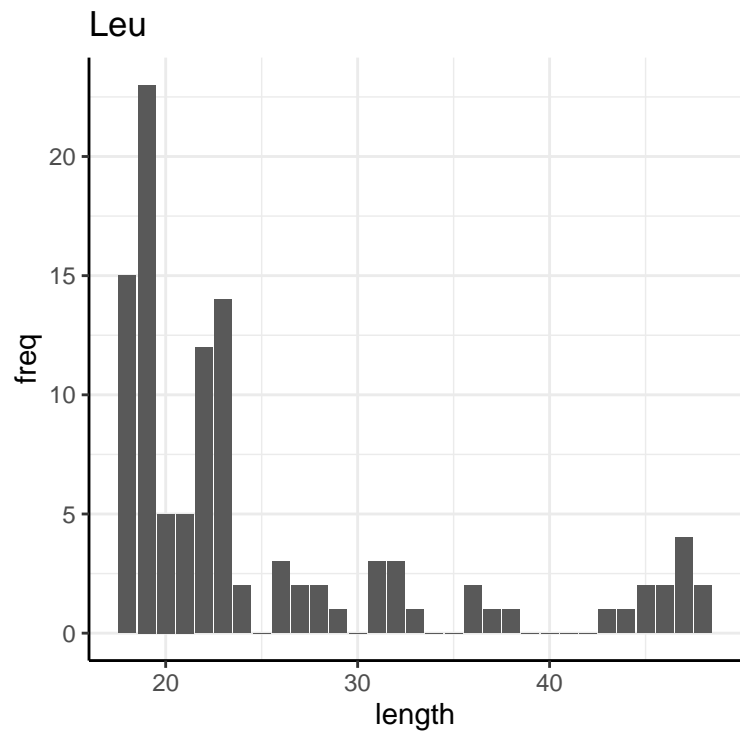

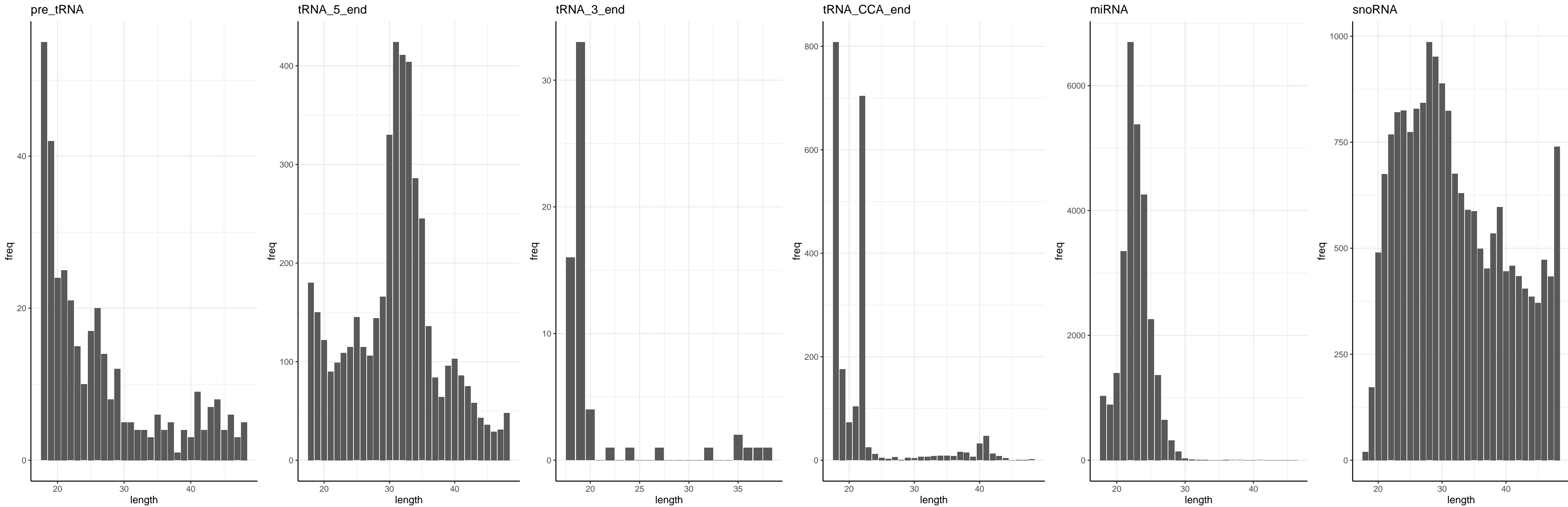

Supplement: gkac022_Supplemental_Files [file gkac022_supplemental_files.zip › Supplementary file 1.pdf]
